# Supplementary material for: Why big brains? A comparison of models for both primate and carnivore brain size evolution
Source: PLoS One. 2021 Dec 21;16(12):e0261185. doi: 10.1371/journal.pone.0261185 (PMC8691615; doi:10.1371/journal.pone.0261185)
Supplement: S1 File — This document includes information about the extra analyses conducted using different measures of brain size. (DOCX) [file pone.0261185.s001.docx]

**Supplementary analyses**

Additional to the analyses discussed in the main manuscript, further statistical analyses were conducted in order to test how uniform the results were when using different brain size measures.

**Supplementary methods**

**Statistical analyses**

**Brain transformations**

Although the use of residuals in ecological models has received criticism [1, 2], their continued use is still evident. Therefore, we use this method in the analyses for comparative purposes. Phylogenetic generalised least-squares regression analysis (PGLS) was used to regress log brain volume against log body mass, producing residual estimates of relative brain size after accounting for body mass. This was repeated for neocortex and cerebellum volumes. Encephalisation quotients (EQs) were calculated as a further measure of relative brain size. EQs provide a way to quantify the relationship between brain size and body mass, calculated as the ratio of observed brain size to expected brain size, which can used to compare a wide range of species of varying body mass [3]. We calculated EQs using the allometric formula E = kPα, where E = brain mass, P = body mass, k = y‐intercept (proportionality constant) and α = allometric exponent. For the primate data this made the final equation: brain volume / (0.073 x body mass^0.80^). For the carnivore data this made the final equation: brain volume / (0.145 x body mass^0.65^).

This allowed for a further four different brain calculation inputs: (1) relative brain size, (2) encephalisation quotients, (3) relative neocortex volume, and (4) relative cerebellum volume.

**PGLS analysis**

Phylogenetic generalised least-squares regression (PGLS) analysis was used to identify those variables influencing whole and regional brain size, using various measures of brain size.

**Model comparisons**

The same model comparison procedure outlined and discussed in the main manuscript was followed using the four additional inputs.

**Results**

**Primates**

The results of the additional PGLS analysis on the primate data is shown in Table S1. Most models were significant. For whole brain and neocortex models, λ was close to one; however, within the cerebellum models λ was equal to zero. Combined models were preferred when investigating both whole and regional brain volumes, with significantly improved BIC scores, except in the encephalisation quotient analyses, where ecological models were preferred, and in the relative cerebellum size analyses, where no significant difference was found between the ecological, social & ecological and combined models. When simply comparing the influence of ecology versus sociality, ecological models were found to be preferable to social models, evidenced by the presence of significantly improved BIC scores.

**Overall encephalisation**

The results of the PGLS analysis on relative brain size and encephalisation quotient data are presented in Table S1, with the ‘*best fit*’ models presented in Table S2. The variables which were indicated to be of importance and included within the ‘*best fit*’ relative brain size models were: dietary breadth and gestation length. Also present in the subset of ‘*best fit*’ models were: social cohesion, maximum longevity and weaning age. After accounting for phylogeny, dietary breadth was found to be positively associated with RBS (P <0.001). Gestation length, maximum longevity and weaning age were also found to be associated with RBS (P <0.01, P = <0.05, P = <0.01). Although, maximum longevity and weaning age failed to find significance in certain model iterations (P = 0.06). In terms of the social variables, social cohesion also failed to reach significance (P = 0.06). The variable which was indicated to be of importance and included within the ‘*best fit*’ encephalisation quotient models was dietary breadth. After accounting for phylogeny, dietary breadth was found to be significantly associated with EQ (P <0.01).

**Regional brain volumes**

The results of the PGLS analysis on the relative neocortex and cerebellum data are presented in Table S1, with the ‘*best fit*’ models presented in Table S2. The variables which were indicated to be of importance and included within the ‘*best fit*’ relative neocortex models were: diet and gestation length. Also present in the subset of ‘*best fit*’ models were: weaning age and fertility. After accounting for phylogeny, diet, specifically frugivory and omnivory, were found to be positively associated with relative neocortex volume (P <0.001). This is the result produced when a folivorous diet is used as the baseline category, therefore the dietary category results produced here only demonstrates differences between these dietary groups (frugivory and omnivory) and folivory. Three of the life-history variables were significantly associated with RNS, gestation length, weaning age and fertility (P <0.01), with fertility being negatively associated.

The variables which were indicated to be of importance and included within the ‘*best fit*’ relative cerebellum models were: diet, home range and fertility. Also present in the subset of ‘*best fit*’ models was: weaning age, age at first reproduction and social cohesion. After accounting for phylogeny, diet, specifically frugivory and omnivory, were found to be positively associated with RCS (P <0.01, P <0.001). As above, this results when folivorous diet is used as the baseline category. In addition to diet, home range was also found to be associated with RCS (P <0.001), but this association failed to remain significant in certain model iterations (P = 0.05, P = 0.2, P = 0.3). In terms of the life-history variables, fertility was significantly negatively associated with RCS (P <0.05), however, both weaning age and age at first reproduction failed to reach significance (P = 0.07, P = 0.09). Similarly, social cohesion also failed to reach significance (P = 0.13).

**Carnivores**

The results of the additional PGLS analysis on the carnivore data is shown in Table S3. When using different body size transformation methods, only certain models were significant. Lambda was not consistent between the models, ranging from one to zero across the dataset. In terms of the ‘*best fit*’ models, in the whole brain analyses, there was no significant difference between the ecological, life history and combined models, and thus the results of all these models are discussed below. In the regional brain analyses, the life history model was preferred when investigating the neocortex, whereas the social model was preferred when looking at the cerebellum. When simply comparing the influence of ecology versus sociality, there was typically no significant difference between social and ecology models, aside from the cerebellum analyses where social models produced significantly lower BIC scores.

**Overall encephalisation**

The results of PGLS analysis on relative brain size and encephalisation quotient data are presented in Table S3, with the ‘*best fit*’ models presented in Table S4. The variables which were indicated to be of importance and included within the ‘*best fit*’ relative brain size models were: dietary breadth and fertility. Also present in the subset of ‘*best fit*’ models was: weaning age. After accounting for phylogeny, fertility was found to be negatively associated with RBS (P <0.05). Dietary breadth was close to significance, with the relationship being negatively directed (P = 0.07). Weaning age also failed to reach significance (P = 0.12). The variables which were indicated to be of importance and included in the ‘*best fit*’ encephalisation quotient models were: fertility and weaning age. After accounting for phylogeny, both fertility and weaning age were found to be significantly associated with EQ, with this relationship being negatively directed (P <0.05).

**Regional brain volumes**

The results of the PGLS analysis on the relative neocortex and cerebellum data are presented in Table S3, with the ‘*best fit*’ models presented in Table S4. The variables which were indicated to be of importance and included within the ‘best fit’ relative neocortex models were fertility and weaning age. Also present in the subset of ‘*best fit*’ models were age at first reproduction and different iterations of the previously mentioned variables. After accounting for phylogeny, all three life history variables, fertility, weaning age and age at first reproduction, were significantly negatively associated with RNS (P <0.001, P <0.01, P <0.05). Although both weaning age and age at first reproduction failed to find significance in certain model iterations (P = 0.06, P = 0.14).

The variable which was indicated to be of importance and included within the ‘*best fit*’ relative cerebellum models was: social cohesion. Also present in the subset of ‘*best fit*’ models was: group size. After accounting for phylogeny, both social cohesion and group size were found to be significantly negatively associated with RCS (P <0.05).

**Discussion**

**Primates**

Similar to the results discussed in the main manuscript, here, we find evidence for consistent associations between ecological variables and brain size. This further reinforces the suggestion that diet influences brain size, especially in terms of how food variety, including spatial and temporal availability, can be important in driving changes in brain size [4-11]. However, in contrast with the regional brain volume results produced, the correlations found between relative neocortex, cerebellum and dietary categories were positively correlated. These results are more consistent with the literature (see [12]); however, this does not mean they necessarily are ‘*true*’. We think it points towards the methods used for measuring regional brain volume warranting greater investigation. In terms of the other ecological variables, the home range associations which were previously uncovered and thought to reflect the cognitive demands imposed by larger home range sizes [6, 13, 14], also remain present when using these additional methods, reinforcing support for the suggestion that larger home range sizes may influence brain size.

In terms of the social associations which were found (see discussion in the main manuscript), these failed to remain significant when using different brain transformation methods. That seemingly important link would have been missed if choosing to use these techniques, which perhaps explains the reason for associations between brain size and sociality being missed in previous studies (see [15, 16]). Instead, a potential link was found between relative cerebellum size and social cohesion, which was not present when using the ‘rest of brain’ technique, as seen in the main manuscript. This association is consistent with current research suggesting the cerebellum is important in social intelligence [17]. However, these associations, also failed to find significance.

Consistent with the previously discussed life history results, here, we find support for correlations between life history variables and brain size. Additionally, negative correlations are found between fertility and regional brain volumes. These relationships were not present in the main results, and yet, they are consistent with the literature, in suggesting that a reduction in energy allocation, ergo reproductive output, is necessary in order to meet the costs of increased brain size [18].

**Carnivores**

In contrast to the ecological results discussed in the main manuscript, specifically the link between cerebellum volume and home range size, here, we fail to find support for this association when using different measures of brain size. Thus, if these brain size measures had been used, this association would have been missed, further supporting the notion that residuals may be inappropriate for use in ecological models (see [1, 2]). In terms of other ecological variables, the negative relationship between brain size and dietary breadth remained present when using these different techniques, albeit still not significant, again suggesting greater dietary breadth is actually associated with smaller brain size in carnivores.

Further reinforcing our main results which show no support for the social brain hypothesis in carnivores, we additionally find no evidence for a link between brain size and sociality when using different measures of brain size. Interestingly, we in fact, uncover a negative correlation between relative cerebellum size, social cohesion and group size. This further confirms the idea that sociality appears to be limited to a select few taxa in carnivores (see [19]) and suggests sociality may not hold the same importance in this group, especially when compared to primates.

In terms of the life history variables, similar results were produced when using different measures of brain size, however notable differences were also present. Both gestation length and maximum lifespan associations which were previously identified, failed to remain present during further analyses. These associations are particularly important in terms of confirming how an increase in developmental periods as well as an extension in reproductive lifespans, are both necessary in order to counterbalance the costs associated with increased brain size [18, 20-22]. Interestingly, an association is still found between age at first reproduction and relative neocortex size, however the relationship is now negatively correlated, whereas it was positively directed in the main results discussed in the manuscript. This raises concerns in terms of the correct way to measure regional brain volumes, and, specifically, which correlation is accurately describing the relationship between life history variables and brain volumes. An additional association is also found when using different brain size measures: weaning age was found to be positively associated with both encephalisation quotient and relative neocortex size. These correlations were not present in our main results, and thus, may be missed when using only those methods or may not be ‘*true*’ correlates of brain size but rather the result of inaccurate body size correction factors.

**Brain size confusion**

Choosing the suitable body size correction factor for use in studies of brain evolution has been highlighted as a complex problem [15, 23]. The disparity in results of comparative analyses of brain evolution could be in part the result of the use of different correction measures. For example, earlier studies used residuals from regression of brain size on body mass, whereas more recent studies are often scaled using residuals from regression of brain size on another brain structure or are taken from ratios [24]. Whilst the body size correction methods used here produced similar outputs in certain instances, differences were also present, which raises questions regarding the use of different body size correction factors. For example, in primates, the associations between dietary categories and regional brain volumes were found to be negative directed when using the ‘rest of brain’ technique, whereas these associations were positively driven when using residuals as relative neocortex or cerebellum size. Whilst the ‘rest of brain’ technique is thought to be a superior method for measuring regional brain volume (as seen in [12]), the results produced using residuals are more consistent with the literature, and coincide with the suggestion that certain dietary categories are associated with increased encephalisation due to the cognitive foraging demands imposed by certain diets such as frugivory and omnivory [12]. However, just because the results are more consistent it does not necessary mean they are ‘true’. It calls into question the techniques used to measure regional brain volumes and specifically, whether the ‘rest of brain’ technique is sufficiently accounting for changes in body size.

Another example is the association between cerebellum volume and home range size, found in carnivores, which fails to remain present when using different techniques. This highlights concerns. First, without the inclusion of this method (which in this instance is the ‘rest of brain’ technique), the association would have been missed. Second, it raises questions regarding the validity of the association. Without clarity regarding the most suitable correction measure, it is difficult to ascertain which associations are ‘*true*’ correlates and which do not actually invoke influential change in brain evolution but are rather the consequence of inaccurate correction methods. This confusion regarding correction measures needs addressing, especially in terms of measuring regional brain volumes, with the aim of determining the superior method, allowing greater clarity on past and current research whilst guiding future comparative analyses. For example, the fact that most carnivore models using residuals or other statistical calculations failed to be significant is noteworthy. Residuals appear to fail to appropriately account for body size in carnivores, or rather, as previously mentioned, brain estimates appear to be a poor representation of carnivoran brains due to the fact that carnivore brain size shows a lag relative to body size over evolutionary time [15]. Thus, inputting body size into a model as a covariate, rather than using any other brain estimate, appears most appropriate when designing comparative analyses of carnivoran brain evolution.

**Conclusion**

To conclude, the additional analyses conducted here provide evidence that different body size correction factors produce disparate results. This hinders modern research, as without clarity regarding the most appropriate methods, it is difficult to determine which are the ‘*true*’ correlates of brain size, in comparison to those which are produced using inaccurate methods. The use of residuals and encephalisation quotient scores as relative brain size, appears to produce inaccurate results, and thus, as highlighted by the literature, should be discouraged from further use. Greater clarity is needed regarding the best method to estimate regional brain volumes, as the two methods used here produced disparate results, especially in terms of the direction of the relationship between primate neocortex, cerebellum and dietary categories.

**References**

1. Freckleton RP. On the misuse of residuals in ecology: regression of residuals vs. multiple regression. J Anim Ecol. 2002;71(3): 542-545.

2. Freckleton RP. The seven deadly sins of comparative analysis. J Evol Biol. 2009;22(7): 1367-1375.

3. Boddy AM, McGowen MR, Sherwood CC, Grossman LI, Goodman M, Wildman DE. Comparative analysis of encephalization in mammals reveals relaxed constraints on anthropoid primate and cetacean brain scaling. J Evol Biol. 2012;25(5): 981-994.

4. Parker ST, Gibson KR. Object manipulation, tool use and sensorimotor intelligence as feeding adaptations in cebus monkeys and great apes. J Hum Evol. 1977;6(7): 623-641.

5. Harvey PH, Clutton-Brock TH, Mace GM. Brain size and ecology in small mammals and primates. PNAS. 1980;77(7): 4387-4389.

6. Clutton-Brock TH, Harvey PH. Primates, brains and ecology. J Zool. 1980;190(3): 309-323.

7. Milton K. Distribution patterns of tropical plant foods as an evolutionary stimulus to primate mental development. Am Anthropol. 1981;83(3): 534-548.

8. Mace GM, Harvey PH, Clutton-Brock TH. Brain size and ecology in small mammals. J Zool. 1981;193(3): 333-354.

9. Barton RA, Purvis A, Harvey PH. Evolutionary radiation of visual and olfactory brain systems in primates, bats and insectivores. Philos Trans R Soc B. 1995;348(1326): 381-392.

10. Barton RA. Primate brain evolution: cognitive demands of foraging or of social life? In: Boinski S, Garber PA, editors. On the move: how and why animals travel in groups. London: The University of Chicago Press; 2000. pp. 204-237.

11. van Woerden JT, van Schaik CP, Isler K. Effects of seasonality on brain size evolution: evidence from strepsirrhine primates. Am Nat. 2010;176(6): 758-767.

12. DeCasien AR, Williams SA, Higham JP. Primate brain size is predicted by diet but not sociality. Nat Ecol Evol. 2017;1(5): 0112.

13. Parker ST. Re-evaluating the extractive foraging hypothesis. New Ideas Psychol. 2015;37: 1-12.

14. Powell LE, Isler K, Barton RA. Re-evaluating the link between brain size and behavioural ecology in primates. Proc R Soc B. 2017;284(1865): 20171765.

15. Swanson EM, Holekamp KE, Lundrigan BL, Arsznov BM, Sakai ST. Multiple determinants of whole and regional brain volume among terrestrial carnivorans. PLoS One. 2012;7(6): e38447.

16. MacLean EL, Hare B, Nunn CL, Addessi E, Amici F, Anderson RC, et al. The evolution of self-control. PNAS. 2014;111(20): E2140-2148.

17. Barton RA. Embodied cognitive evolution and the cerebellum. Philos Trans R Soc B. 2012;367(1599): 2097-2107.

18. Isler K, van Schaik CP. The expensive brain: a framework for explaining evolutionary changes in brain size. J Hum Evol. 2009;57(4): 392-400.

19. Sakai ST, Arsznov BM. Carnivoran brains: effects of sociality on inter- and intraspecific comparisons of regional brain volumes. In: Kaas JH, editor. Evolutionary neuroscience. 2nd ed. London: Academic Press; 2020. pp. 463-479.

20. Martin RD. Scaling of the mammalian brain: the maternal energy hypothesis. Physiology. 1996;11(4): 149-156.

21. Barton RA, Capellini I. Maternal investment, life histories, and the costs of brain growth in mammals. PNAS. 2011;108(15): 6169-6174.

22. Heldstab S, Isler K. Environmental seasonality and mammalian brain size evolution: Wiley Online Library; 2019.

23. van Schaik CP, Triki Z, Bshary R, Heldstab SA. A farewell to EQ: A new brain size measure for comparative primate cognition. bioRxiv. 2021: 2021.2002.2015.431238.

24. Deaner RO, Nunn CL, van Schaik CP. Comparative tests of primate cognition: different scaling methods produce different results. Brain Behav Evol. 2000;55(1): 44-52.
