# Supplementary material for: Why big brains? A comparison of models for both primate and carnivore brain size evolution
Source: PLoS One. 2021 Dec 21;16(12):e0261185. doi: 10.1371/journal.pone.0261185 (PMC8691615; doi:10.1371/journal.pone.0261185)
Supplement: S2 File — This document includes all the supplementary results tables associated with the supplementary analyses. (DOCX) [file pone.0261185.s002.docx]

Table S1. Phylogenetic generalised least-squares (PGLS) regression analyses examining the effects of social, ecological and life-history variables* on primate whole and regional brain volumes. Preferred models represent the ‘best fit’ model (with the lowest BIC score) of the overall model category (i.e., social or ecological). The combined models represent the ‘best fit’ model after running all combinations of the previous ‘best fit’ models (models one to four). Boldness indicates the model(s) with the lowest BIC score across all models (dBIC<2).

| Brain input | Overall model | Preferred model | BIC score | *P*-value | λ | Adj. r^2^ | Sample size (n) |
| --- | --- | --- | --- | --- | --- | --- | --- |
| Relative brain size | Social | RBS ~ GS | -188.3866 | <0.05 | 1 | 0.03544 | 83 |
|  | Ecological | RBS ~ DB | -195.2419 | <0.01 | 1 | 0.1119 | 83 |
|  | Social & Ecological | RBS ~ SC + DB | -196.1428 | <0.001 | 1 | 0.1566 | 83 |
|  | Life History | RBS ~ ML + WA | -193.7418 | <0.01 | 1 | 0.1319 | 83 |
|  | **Combined** | **RBS ~ DB + GL** | **-201.3805** | **<0.001** | **1** | **0.2082** | **83** |
|  | All | RBS ~ GS + SC + D + DB + HV + HR + GL + ML + F + FR + WA | -178.0563 | <0.001 | 1 | 0.2962 | 83 |
| Encephalisation quotient | Social | EQ ~ GS | -19.20934 | 0.213 | 1 | 0.006972 | 83 |
|  | **Ecological** | **EQ ~ DB** | **-27.13662** | **<0.01** | **1** | **0.09743** | **83** |
|  | Social & Ecological | EQ ~ GS + DB | -23.93969 | <0.01 | 0.997 | 0.1028 | 83 |
|  | Life History | EQ ~ ML | -18.12871 | 0.4782 | 1 | -0.006041 | 83 |
|  | Combined | EQ ~ DB + F | -23.27784 | <0.01 | 1 | 0.09229 | 83 |
|  | All | EQ ~ GS + SC + D + DB + HV + HR + GL + ML + F + FR + WA | 3.168861 | <0.001 | 0.988 | 0.1767 | 83 |
| Relative neocortex size | Social | RNS ~ GS | -60.58831 | 0.1376 | 1 | 0.02443 | 52 |
|  | Ecological | RNS ~ D + HR | -65.45603 | <0.01 | 0.982 | 0.2196 | 52 |
|  | Social & Ecological | RNS ~ SC + D | -64.1511 | <0.01 | 0.971 | 0.209 | 52 |
|  | Life History | RNS ~ F | -61.55354 | 0.08 | 1 | 0.04237 | 52 |
|  | **Combined** | **RNS ~ D + GL** | **-70.76861** | **<0.001** | **0.985** | **0.2933** | **52** |
|  | All | RNS ~ GS + SC + D + DB + HV + HR + GL + ML + F + FR + WA | -46.15279 | <0.01 | 0.889 | 0.3548 | 52 |
| Relative cerebellum size | Social | RCS ~ GS | -63.57364 | <0.05 | 0 | 0.09919 | 49 |
|  | **Ecological** | **RCS ~ D + HR** | **-72.63367** | **<0.001** | **0** | **0.3328** | **49** |
|  | **Social & Ecological** | **RCS ~ SC + D + HR** | **-71.11005** | **<0.001** | **0** | **0.3499** | **49** |
|  | Life History | RCS ~ ML | -61.71095 | 0.13 | 0.122 | 0.02784 | 49 |
|  | **Combined** | **RCS ~ D + HR + F** | **-73.04722** | **<0.001** | **0** | **0.3751** | **49** |
|  | All | RCS ~ GS + SC + D + DB + HV + HR + GL + ML + F + FR + WA | -48.89316 | <0.01 | 0 | 0.3376 | 49 |

*GS = Group size, SC = Social cohesion, D = Diet, DB = Dietary breadth, HV = Habitat variability, HR = Home range, GL = Gestation length, ML = Maximum longevity, F = Fertility, FR = Age at first reproduction, WA = Weaning age.

Table S2. Phylogenetic generalised least-squares (PGLS) regression analyses examining the effects of social, ecological and life-history variables* on primate whole and regional brain volumes. Preferred models represent all the ‘best fit’ models for each brain input, which in most cases represents a subset of models (any model within dBIC<2 of the lowest model). This can include any category of model (i.e., social or combined), and is dependent on the BIC score produced. Boldness indicates <0.05.

| Brain input | Preferred model | BIC score | Predictor | Estimate | *t*-value | *P*-value |
| --- | --- | --- | --- | --- | --- | --- |
| Relative brain size | RBS ~ DB + GL | -201.3805 | Intercept | -1.0521 | -3.4291 | **<0.001** |
|  |  |  | DB | 0.0262 | 3.5657 | **<0.001** |
|  |  |  | LogGL | 0.4458 | 3.2941 | **<0.01** |
|  | RBS ~ DB + GL + ML | <2 | Intercept | -1.0978 | -3.6253 | **<0.001** |
|  |  |  | DB | 0.0253 | 3.4949 | **<0.001** |
|  |  |  | LogGL | 0.4035 | 2.9895 | **<0.01** |
|  |  |  | LogML | 0.1004 | 1.9084 | 0.06 |
|  | RBS ~ SC + DB + GL | <2 | Intercept | -1.0005 | -3.2992 | **<0.01** |
|  |  |  | SC | 0.0205 | 1.8867 | 0.06 |
|  |  |  | DB | 0.0270 | 3.7258 | **<0.001** |
|  |  |  | LogGL | 0.4038 | 2.9895 | **<0.01** |
|  | RBS ~ DB + GL + WA | <2 | Intercept | -0.9287 | -3.0039 | **<0.01** |
|  |  |  | DB | 0.0256 | 3.5398 | **<0.001** |
|  |  |  | LogGL | 0.3182 | 2.1266 | **<0.05** |
|  |  |  | LogWA | 0.0704 | 1.8757 | 0.06 |
|  | RBS ~ DB + ML + WA | <2 | Intercept | -0.4434 | -3.6483 | **<0.001** |
|  |  |  | DB | 0.0244 | 3.3668 | **<0.01** |
|  |  |  | LogML | 0.1096 | 2.0964 | **<0.05** |
|  |  |  | LogWA | 0.0990 | 2.9447 | **<0.01** |
| Encephalisation quotient | EQ ~ DB | -27.13662 | Intercept | 0.7715 | 3.6781 | **<0.001** |
|  |  |  | DB | 0.0672 | 3.1387 | **<0.01** |
| Relative neocortex size | RNS ~ D + GL | -70.76861 | Intercept | -1.7125 | -3.3436 | **<0.01** |
|  |  |  | DFrug | 0.1771 | 3.7870 | **<0.001** |
|  |  |  | DOmni | 0.2393 | 4.2629 | **<0.001** |
|  |  |  | LogGL | 0.6938 | 3.1143 | **<0.01** |
|  | RNS ~ D + WA | <2 | Intercept | -0.6222 | -3.5326 | **<0.001** |
|  |  |  | DFrug | 0.1696 | 3.7202 | **<0.001** |
|  |  |  | DOmni | 0.2359 | 4.3113 | **<0.001** |
|  |  |  | LogWA | 0.1951 | 3.1903 | **<0.01** |
|  | RNS ~ D + F | <2 | Intercept | -0.1842 | -1.7952 | 0.08 |
|  |  |  | DFrug | 0.1526 | 3.2438 | **<0.01** |
|  |  |  | DOmni | 0.2291 | 4.0440 | **<0.001** |
|  |  |  | LogF | -0.1854 | -2.7862 | **<0.01** |
| Relative cerebellum size | RCS ~ D + HR + F | -73.04722 | Intercept | -0.2051 | -4.8400 | **<0.001** |
|  |  |  | DFrug | 0.1366 | 3.3198 | **<0.01** |
|  |  |  | DOmni | 0.2101 | 4.4732 | **<0.001** |
|  |  |  | LogHR | 0.0352 | 1.9870 | 0.05 |
|  |  |  | LogF | -0.1169 | -2.0102 | 0.05 |
|  | RCS ~ D + HR | <2 | Intercept | -0.2022 | -4.6207 | **<0.001** |
|  |  |  | DFrug | 0.1259 | 2.9867 | **<0.01** |
|  |  |  | DOmni | 0.1621 | 3.8792 | **<0.001** |
|  |  |  | LogHR | 0.0547 | 3.5800 | **<0.001** |
|  | RCS ~ D + HR + WA | <2 | Intercept | -0.4264 | -3.3552 | **<0.01** |
|  |  |  | DFrug | 0.1418 | 3.3849 | **<0.01** |
|  |  |  | DOmni | 0.2079 | 4.3802 | **<0.001** |
|  |  |  | LogHR | 0.0272 | 1.3027 | 0.2 |
|  |  |  | LogWA | 0.1045 | 1.8723 | 0.07 |
|  | RCS ~ D + HR + FR | <2 | Intercept | -0.2400 | -4.9827 | **<0.001** |
|  |  |  | DFrug | 0.1229 | 2.9761 | **<0.01** |
|  |  |  | DOmni | 0.1871 | 4.3089 | **<0.001** |
|  |  |  | LogHR | 0.0432 | 2.6365 | **<0.05** |
|  |  |  | LogFR | 0.0786 | 1.7174 | 0.09 |
|  | RCS ~ SC + D + HR + F | <2 | Intercept | -0.2518 | -4.9077 | **<0.001** |
|  |  |  | SC | 0.0230 | 1.5617 | 0.13 |
|  |  |  | DFrug | 0.1388 | 3.4259 | **<0.01** |
|  |  |  | DOmni | 0.2310 | 4.8006 | **<0.001** |
|  |  |  | LogHR | 0.0206 | 1.0417 | 0.3 |
|  |  |  | LogF | -0.1183 | -2.0667 | **<0.05** |

*GS = Group size, SC = Social cohesion, D = Diet, DB = Dietary breadth, HV = Habitat variability, HR = Home range, GL = Gestation length, ML = Maximum longevity, F = Fertility, FR = Age at first reproduction, WA = Weaning age.

Table S3. Phylogenetic generalised least-squares (PGLS) regression analyses examining the effects of social, ecological and life-history variables* on carnivoran whole and regional brain volumes. Preferred models represent the ‘best fit’ model (with the lowest BIC score) of the overall model category (i.e., social or ecological). The combined models represent the ‘best fit’ model after running all combinations of the previous ‘best fit’ models (models one to four). Boldness indicates the model(s) with the lowest BIC score across all models (dBIC<2).

| Brain input | Overall model | Preferred model | BIC score | *P*-value | λ | Adj. r^2^ | Sample size (n) |
| --- | --- | --- | --- | --- | --- | --- | --- |
| Relative brain size | Social | RBS ~ GS | -141.76 | 0.3837 | 0.769 | -0.00278 | 85 |
|  | **Ecological** | **RBS ~ HV** | **-143.2624** | **0.1302** | **0.806** | **0.01567** | **85** |
|  | Social & Ecological | RBS ~ GS + HV | -139.456 | 0.2415 | 0.795 | 0.01051 | 85 |
|  | **Life History** | **RBS ~ F** | **-144.6612** | **0.05** | **0.718** | **0.03371** | **85** |
|  | **Combined** | **RBS ~ DB + F** | **-143.6012** | **<0.05** | **0.750** | **0.0426** | **85** |
|  | All | RBS ~ GS + SC + D + DB + HV + HR + GL + ML + F + FR + WA | -109.2036 | 0.1679 | 0.638 | 0.05925 | 85 |
| Encephalisation quotient | Social | EQ ~ GS | 6.953438 | 0.7669 | 0.510 | -0.01097 | 85 |
|  | **Ecological** | **EQ ~ HV** | **4.703785** | **0.1318** | **0.516** | **0.01543** | **85** |
|  | Social & Ecological | EQ ~ GS + HV | 9.072394 | 0.3121 | 0.511 | 0.004293 | 85 |
|  | **Life History** | **EQ ~ F + WA** | **4.20457** | **<0.05** | **0.514** | **0.05971** | **85** |
|  | Combined | EQ ~ HV + WA | 7.177263 | 0.12 | 0.569 | 0.0274 | 85 |
|  | All | EQ ~ GS + SC + D + DB + HV + HR + GL + ML + F + FR + WA | 41.24089 | 0.3089 | 0.366 | 0.02603 | 85 |
| Relative neocortex size | Social | RNS ~ SC | -43.79829 | 0.8653 | 0.509 | -0.0231 | 44 |
|  | Ecological | RNS ~ HV | -44.95599 | 0.2756 | 0.425 | 0.00509 | 44 |
|  | Social & Ecological | RNS ~ SC + HV | -41.18976 | 0.5493 | 0.420 | -0.01858 | 44 |
|  | **Life History** | **RNS ~ F + WA** | **-50.77517** | **<0.001** | **1** | **0.3381** | **44** |
|  | Combined | RNS ~ HR + F + FR + WA | -48.43799 | <0.001 | 1 | 0.3822 | 44 |
|  | All | RNS ~ GS + SC + D + DB + HV + HR + GL + ML + F + FR + WA | -24.18441 | <0.05 | 0 | 0.254 | 44 |
| Relative cerebellum size | **Social** | **RCS ~ SC** | **-24.45923** | **<0.05** | **0** | **0.1038** | **38** |
|  | Ecological | RCS ~ HV | -19.92905 | 0.4263 | 0 | -0.009617 | 38 |
|  | Social & Ecological | RCS ~ GS + HV | -21.51544 | 0.07 | 0 | 0.09492 | 38 |
|  | Life History | RCS ~ F | -19.84599 | 0.4561 | 0 | -0.01183 | 38 |
|  | Combined | RCS ~ GS + ML | -21.33204 | 0.07 | 0 | 0.09054 | 38 |
|  | All | RCS ~ GS + SC + D + DB + HV + HR + GL + ML + F + FR + WA | 11.89669 | 0.8622 | 0 | -0.172 | 38 |

*GS = Group size, SC = Social cohesion, D = Diet, DB = Dietary breadth, HV = Habitat variability, HR = Home range, GL = Gestation length, ML = Maximum longevity, F = Fertility, FR = Age at first reproduction, WA = Weaning age.

Table S4. Phylogenetic generalised least-squares (PGLS) regression analyses examining the effects of social, ecological and life-history variables* on carnivoran whole and regional brain volumes. Preferred models represent all the ‘best fit’ models for each brain input, which in most cases represents a subset of models (any model within dBIC<2 of the lowest model). This can include any category of model (i.e., social or combined), and is dependent on the BIC score produced. Boldness indicates <0.05.

| Brain input | Preferred model | BIC score | Predictor | Estimate | *t*-value | *P*-value |
| --- | --- | --- | --- | --- | --- | --- |
| Relative brain size | RBS ~ DB + F | -143.6012 | Intercept | 0.0939 | 1.6254 | 0.11 |
|  |  |  | DB | -0.0143 | -1.8291 | 0.07 |
|  |  |  | LogF | -0.1043 | -2.2709 | **<0.05** |
|  | RBS ~ F + WA | <2 | Intercept | 0.2242 | 1.7415 | 0.09 |
|  |  |  | LogF | -0.1287 | -2.4498 | **<0.05** |
|  |  |  | LogWA | -0.0823 | -1.5813 | 0.12 |
|  | RBS ~ DB + F + WA | <2 | Intercept | 0.2754 | 2.1233 | **<0.05** |
|  |  |  | DB | -0.0143 | -1.8465 | 0.07 |
|  |  |  | LogF | -0.1425 | -2.7295 | **<0.01** |
|  |  |  | LogWA | -0.0817 | -1.5901 | 0.12 |
| Encephalisation quotient | EQ ~ F + WA | 4.20457 | Intercept | 1.6963 | 5.6764 | **<0.001** |
|  |  |  | LogF | -0.2717 | -2.1851 | **<0.05** |
|  |  |  | LogWA | -0.3146 | -2.5326 | **<0.05** |
| Relative neocortex size | RNS ~ F + WA | -50.77517 | Intercept | 0.4984 | 2.6472 | **<0.05** |
|  |  |  | LogF | -0.3592 | -4.7586 | **<0.001** |
|  |  |  | LogWA | -0.1817 | -2.7503 | **<0.01** |
|  | RNS ~ F + FR + WA | <2 | Intercept | 0.4538 | 2.4146 | **<0.05** |
|  |  |  | LogF | -0.3764 | -5.0007 | **<0.001** |
|  |  |  | LogFR | -0.1479 | -1.4910 | 0.14 |
|  |  |  | LogWA | -0.1368 | -1.9057 | 0.06 |
|  | RNS ~ F + FR | <2 | Intercept | 0.1678 | 1.4377 | 0.16 |
|  |  |  | LogF | -0.3404 | -4.5288 | **<0.001** |
|  |  |  | LogFR | -0.2273 | -2.4487 | **<0.05** |
|  | RNS ~ F | <2 | Intercept | 0.0605 | 0.7930 | 0.43 |
|  |  |  | LogF | -0.2100 | -2.4896 | **<0.05** |
| Relative cerebellum size | RCS ~ SC | -24.45923 | Intercept | 0.0939 | 1.9304 | 0.06 |
|  |  |  | SC | -0.0476 | -2.2995 | **<0.05** |
|  | RCS ~ GS | <2 | Intercept | 0.0397 | 1.2497 | 0.22 |
|  |  |  | LogGS | -0.1314 | -2.2642 | **<0.05** |

*GS = Group size, SC = Social cohesion, D = Diet, DB = Dietary breadth, HV = Habitat variability, HR = Home range, GL = Gestation length, ML = Maximum longevity, F = Fertility, FR = Age at first reproduction, WA = Weaning age.
