# Supplementary material for: Why big brains? A comparison of models for both primate and carnivore brain size evolution
Source: PLoS One. 2021 Dec 21;16(12):e0261185. doi: 10.1371/journal.pone.0261185 (PMC8691615; doi:10.1371/journal.pone.0261185)
Supplement: S6 File — This document includes all the VIF score results. (DOCX) [file pone.0261185.s006.docx]

**Supplementary Results Tables**

**Variance Inflation Factor (VIF) Results**

**Primates**

Table 1. Variance Inflation Factor (VIF) analysis examining the presence of multicollinearity in the endocranial volume best fit model.

| Brain input | Preferred model | VIF score | | | | | |
| --- | --- | --- | --- | --- | --- | --- | --- |
|  |  | LogMass | GS | DB | LogGL | LogML | LogWA |
| ECV | ECV ~ Mass + GS + DB + GL + ML + WA | 6.489816 | 1.726200 | 1.397262 | 2.416859 | 1.807539 | 6.025752 |

Table 2. Variance Inflation Factor (VIF) analysis examining the presence of multicollinearity in the neocortex best fit model.

| Brain input | Preferred model | VIF score | | | | |
| --- | --- | --- | --- | --- | --- | --- |
|  |  | DFrug | DOmni | LogHR | LogML | LogWA |
| Neo | Neo ~ D + HR + ML + WA | 2.405917 | 2.720020 | 2.224089 | 1.785968 | 2.937396 |

Table 3. Variance Inflation Factor (VIF) analysis examining the presence of multicollinearity in the cerebellum best fit model.

| Brain input | Preferred model | VIF score | | | | |
| --- | --- | --- | --- | --- | --- | --- |
|  |  | DFrug | DOmni | LogHR | LogML | LogWA |
| Cere | Cere ~ D + HR + ML + WA | 2.631917 | 2.964985 | 2.277109 | 1.819821 | 3.005958 |

Table 4. Variance Inflation Factor (VIF) analysis examining the presence of multicollinearity in the relative brain size best fit model.

| Brain input | Preferred model | VIF score | |  |
| --- | --- | --- | --- | --- |
|  |  | SC | DB | LogGL |
| RBS | RBS ~ SC + DB + GL | 1.206182 | 1.027200 | 1.188975 |

Table 5. Variance Inflation Factor (VIF) analysis examining the presence of multicollinearity in the encephalisation quotient best fit model.

| Brain input | Preferred model | VIF score |
| --- | --- | --- |
|  |  | DB |
| EQ | EQ ~ DB | ---- |

Table 6. Variance Inflation Factor (VIF) analysis examining the presence of multicollinearity in the relative neocortex size best fit model.

| Brain input | Preferred model | VIF score | | |
| --- | --- | --- | --- | --- |
|  |  | DFrug | DOmni | LogGL |
| RNS | RNS ~ D + GL | 1.853653 | 2.031636 | 1.142482 |

Table 7. Variance Inflation Factor (VIF) analysis examining the presence of multicollinearity in the relative cerebellum size best fit model.

| Brain input | Preferred model | VIF score | | | |
| --- | --- | --- | --- | --- | --- |
|  |  | SC | DFrug | DOmni | LogWA |
| RCS | RCS ~ SC + D + WA | 1.506936 | 2.086203 | 2.702516 | 1.718597 |

**Carnivores**

Table 8. Variance Inflation Factor (VIF) analysis examining the presence of multicollinearity in the endocranial volume best fit model.

| Brain input | Preferred model | VIF score | |
| --- | --- | --- | --- |
|  |  | LogMass | LogF |
| ECV | ECV ~ Mass + F | 1.754442 | 1.754442 |

Table 9. Variance Inflation Factor (VIF) analysis examining the presence of multicollinearity in the neocortex best fit model.

| Brain input | Preferred model | VIF score | |
| --- | --- | --- | --- |
|  |  | LogML | LogFR |
| Neo | Neo ~ ML + FR | 2.641224 | 2.641224 |

Table 10. Variance Inflation Factor (VIF) analysis examining the presence of multicollinearity in the cerebellum best fit model.

| Brain input | Preferred model | VIF score | |  |  |  |
| --- | --- | --- | --- | --- | --- | --- |
|  |  | LogHR | LogGL | LogML | LogFR | |
| Cere | Cere ~ HR + GL + ML + FR | 1.879330 | 2.932130 | 3.031380 | 5.025514 |  |

Table 11. Variance Inflation Factor (VIF) analysis examining the presence of multicollinearity in the relative brain size best fit model.

| Brain input | Preferred model | VIF score | |
| --- | --- | --- | --- |
|  |  | DB | LogF |
| RBS | RBS ~ DB + F | 1.000158 | 1.000158 |

Table 12. Variance Inflation Factor (VIF) analysis examining the presence of multicollinearity in the encephalisation quotient best fit model.

| Brain input | Preferred model | VIF score | |
| --- | --- | --- | --- |
|  |  | LogF | LogWA |
| EQ | EQ ~ F + WA | 2.027192 | 2.027192 |

Table 13. Variance Inflation Factor (VIF) analysis examining the presence of multicollinearity in the relative neocortex size best fit model.

| Brain input | Preferred model | VIF score | |
| --- | --- | --- | --- |
|  |  | LogF | LogWA |
| RNS | RNS ~ F + WA | 2.006435 | 2.006435 |

Table 14. Variance Inflation Factor (VIF) analysis examining the presence of multicollinearity in the relative cerebellum size best fit model.

| Brain input | Preferred model | VIF score |
| --- | --- | --- |
|  |  | SC |
| RCS | RCS ~ SC | --- |
