# Supplementary material for: Why big brains? A comparison of models for both primate and carnivore brain size evolution
Source: PLoS One. 2021 Dec 21;16(12):e0261185. doi: 10.1371/journal.pone.0261185 (PMC8691615; doi:10.1371/journal.pone.0261185)
Supplement: S7 File — This document includes all the data collection sources. (DOCX) [file pone.0261185.s007.docx]

**Data collection sources**

**Primates**

**Body mass and endocranial volume:**

Powell, L.E., Isler, K., Barton, R.A. (2017). Re-evaluating the link between brain size and behavioural ecology in primates. *Proceedings of the Royal Society B: Biological Sciences, 284*(1865), 20171765. Doi: 10.1098/rspb.2017.1765.

Isler, K., van Schaik, C.P. (2012). Allomaternal care, life history and brain size evolution in mammals. *Journal of Human Evolution, 63*(1), 52-63. Doi: 10.1016/j.jhevol.2012.03.009.

**Neocortex and cerebellum volume:**

DeCasien, A.R., Higham, J.P. (2019). Primate mosaic brain evolution reflects selection on sensory and cognitive specialization. *Nature Ecology & Evolution, 3*(10), 1483-1493. Doi: 10.1038/s41559-019-0969-0.

**Social information:**

DeCasien, A.R., Higham, J.P. (2019). Primate mosaic brain evolution reflects selection on sensory and cognitive specialization. *Nature Ecology & Evolution, 3*(10), 1483-1493. Doi: 10.1038/s41559-019-0969-0.

**Diet:**

DeCasien, A.R., Higham, J.P. (2019). Primate mosaic brain evolution reflects selection on sensory and cognitive specialization. *Nature Ecology & Evolution, 3*(10), 1483-1493. Doi: 10.1038/s41559-019-0969-0.

Wilman, H., Belmarker, J., Simpson, J., de la Rosa, C., Rivadeneira, M.M., Jetz, W. (2014). EltonTraits 1.0: Species‐level foraging attributes of the world's birds and mammals. *Ecology, 95*(7), 2027. Doi: 10.1890/13-1917.1.

**Home range:**

Powell, L.E., Isler, K., Barton, R.A. (2017). Re-evaluating the link between brain size and behavioural ecology in primates. *Proceedings of the Royal Society B: Biological Sciences, 284*(1865), 20171765. Doi: 10.1098/rspb.2017.1765.

**Habitat variability:**

IUCN Red List. (2020). *The IUCN Red List of Threatened Species*. Retrieved from: https://www.iucnredlist.org/.

**Life history variables:**

Isler, K., van Schaik, C.P. (2012). Allomaternal care, life history and brain size evolution in mammals. *Journal of Human Evolution, 63*(1), 52-63. Doi: 10.1016/j.jhevol.2012.03.009.

Gonzalez‐Voyer, A., González‐Suárez, M., Vilà, C., Revilla, E. (2016). Larger brain size indirectly increases vulnerability to extinction in mammals. *Evolution, 70*(6), 1364-1375. Doi: 10.1111/evo.12943.

Lewitus, E., Kelava, I., Kalinka, A.T., Tomancak, P., Huttner, W.B. (2014). An Adaptive Threshold in Mammalian Neocortical Evolution. *PLoS Biology, 12*(11), e1002000. Doi: 10.1371/journal.pbio.1002000.

**Carnivores**

**Body mass and endocranial volume:**

Heldstab, S.A., Isler, K., van Schaik, C.P. (2018). Hibernation constrains brain size evolution in mammals. *Journal of Evolutionary Biology, 31*(10), 1582-1588. Doi: 10.1111/jeb.13353.

Finarelli, J.A., Flynn, J.J. (2009). Brain-size evolution and sociality in Carnivora. *PNAS, 106*(23), 9345-9349. Doi: 10.1073/pnas.0901780106.

**Neocortex and cerebellum volume:**

Dunbar, R.I.M., Bever, J. (1998). Neocortex Size Predicts Group Size in Carnivores and Some Insectivores. *Ethology, 104*(8), 695-708. Doi: 10.1111/j.1439-0310.1998.tb00103.x.

Swanson, E.M., Holekamp, K.E., Lundrigan, B.L., Arsznov, B.M., Sakai, S.T. (2012). Multiple Determinants of Whole and Regional Brain Volume among Terrestrial Carnivorans. *PLoS ONE, 7*(6), e38447. Doi: 10.1371/journal.pone.0038447.

Reep, R.L., Finlay, B.L., Darlington, R.B. (2007). The Limbic System in Mammalian Brain Evolution. *Brain, Behavior and Evolution, 70*(1), 57-70. Doi: 10.1159/000101491.

Sakai, S.T., Arsznov, B.M., Hristova, A.E., Yoon, E.J., Lundrigan, B.L. (2016). Big Cat Coalitions: A Comparative Analysis of Regional Brain Volumes in Felidae. *Frontiers in Neuroanatomy, 10*, 99. Doi: 10.3389/fnana.2016.00099.

Arsznov, B.M., Sakai, S.T. (2013). The procyonid social club: comparison of brain volumes in the coatimundi (Nasua nasua, N. narica), kinkajou (Potos flavus), and raccoon (Procyon lotor). *Brain, Behavior and Evolution, 82*(2), 129-145. Doi: 10.1159/000354639.

Smaers, J.B., Turner, A.H., Gomez-Robles, A., Sherwood, C.C. (2018). A cerebellar substrate for cognition evolved multiple times independently in mammals. *eLife, 7*, e35696. Doi: 10.7554/eLife.35696.

**Social information:**

Gittleman, J.L. (1989). Carnivore group-living: comparative trends. In J.L. Gittleman (Ed.), *Carnivore behavior, ecology, and evolution* (183-207). New York: Cornell University Press.

Nowak, R.M. (2005). *Walker’s carnivores of the world*. Baltimore: Johns Hopkins University Press.

Stankowich, T., Haverkamp, P.J., Caro, T. (2014). Ecological drivers of antipredator defences in carnivores. *Evolution, 68*(5), 1415-1425. Doi: 10.1111/evo.12356.

**Diet:**

Isler, K., van Schaik, C.P. (2012). Allomaternal care, life history and brain size evolution in mammals. *Journal of Human Evolution, 63*(1), 52-63. Doi: 10.1016/j.jhevol.2012.03.009.

Tucker, M.A., Ord, T.J., Rogers, T.L. (2014). Evolutionary predictors of mammalian home range size: body mass, diet and the environment. *Global Ecology and Biogeography, 23*(10), 1105-1114. Doi: 10.1111/geb.12194.

Law, C.J., Duran, E., Hung, N., Richards, E., Santillan, I., Mehta, R.S. (2018). Effects of diet on cranial morphology and biting ability in musteloid mammals. *Journal of Evolutionary Biology, 31*(12), 1918-1931. Doi: 10.1111/jeb.13385.

Wilman, H., Belmarker, J., Simpson, J., de la Rosa, C., Rivadeneira, M.M., Jetz, W. (2014). EltonTraits 1.0: Species‐level foraging attributes of the world's birds and mammals. *Ecology, 95*(7), 2027. Doi: 10.1890/13-1917.1.

**Home range:**

Gittleman, J.L., Harvey, P.H. (1982). Carnivore home-range size, metabolic needs and ecology. *Behavioral Ecology and Sociobiology, 10*, 57-63. Doi: 10.1007/BF00296396.

Heldstab, S.A., Isler, K., van Schaik, C.P. (2018). Hibernation constrains brain size evolution in mammals. *Journal of Evolutionary Biology, 31*(10), 1582-1588. Doi: 10.1111/jeb.13353.

**Habitat variability:**

IUCN Red List. (2020). *The IUCN Red List of Threatened Species*. Retrieved from: https://www.iucnredlist.org/.

**Life history variables:**

Isler, K., van Schaik, C.P. (2012). Allomaternal care, life history and brain size evolution in mammals. *Journal of Human Evolution, 63*(1), 52-63. Doi: 10.1016/j.jhevol.2012.03.009.

Gonzalez‐Voyer, A., González‐Suárez, M., Vilà, C., Revilla, E. (2016). Larger brain size indirectly increases vulnerability to extinction in mammals. *Evolution, 70*(6), 1364-1375. Doi: 10.1111/evo.12943.

Myhrvold, N.P., Baldridge, E., Chan, B., Sivan, D., Freeman, D.L., Ernest, S.K.M. (2015). An amniote life‐history database to perform comparative analyses with birds, mammals, and reptiles. *Ecology, 96*(11), 3109.

Noonan, M.J., Newman, C., Buesching, C.D., Macdonald, D.W. (2015). Evolution and function of fossoriality in the Carnivora: implications for group-living. *Frontiers in Ecology and Evolution, 3*, 116. Doi: 10.3389/fevo.2015.00116.
