# Supplementary material for: Why big brains? A comparison of models for both primate and carnivore brain size evolution
Source: PLoS One. 2021 Dec 21;16(12):e0261185. doi: 10.1371/journal.pone.0261185 (PMC8691615; doi:10.1371/journal.pone.0261185)
Supplement: Attachment — Submitted filename: Review_PONE-D-21-12399_R1_reviewer.pdf [file pone.0261185.s012.pdf]

PLOS ONE

Why big brains? A comparison of models for both primate and carnivore brain size evolution

--Manuscript Draft--

**Manuscript Number:**	PONE-D-21-12399R1
**Article Type:**	Research Article
**Full Title:**	Why big brains? A comparison of models for both primate and carnivore brain size evolution
**Short Title:**	Why big brains?
**Corresponding Author:**	Helen Rebecca Chambers<br>University of Salford<br>Salford, Greater Manchester UNITED KINGDOM
**Keywords:**	Brain size; primates; carnivores; sociality; diet; encephalisation
**Abstract:**	Despite decades of research, much uncertainty remains regarding the selection pressures responsible for brain size variation. Whilst the influential social brain hypothesis once garnered extensive support, more recent studies have failed to find support for a link between brain size and sociality. Instead, it appears there is now substantial evidence suggesting ecology better predicts brain size in both primates and carnivores. Here, different models of brain evolution were tested, and the relative importance of social, ecological, and life-history traits were assessed on both overall encephalisation and specific brain regions. In primates, evidence is found for consistent associations between brain size and ecological factors, particularly diet; however, evidence was also found advocating sociality as a selection pressure driving brain size. In carnivores, evidence suggests ecological variables, most notably home range size, are influencing brain size; whereas, no support is found for the social brain hypothesis, perhaps reflecting the fact sociality appears to be limited to a select few taxa. Life-history associations reveal complex selection mechanisms to be counterbalancing the costs associated with expensive brain tissue through extended developmental periods, reduced fertility, and extended maximum lifespan. Future studies should give careful consideration of the methods chosen for measuring brain size, investigate both whole brain and specific brain regions where possible, and look to integrate multiple variables, thus fully capturing all of the potential factors influencing brain size.
**Order of Authors:**	Helen Rebecca Chambers
	Sandra Andrea Heldstab
	Sean J O'Hara
**Response to Reviewers:**	Reviewer #1<br>1) I am not sure why this manuscript deals with just primates and carnivores. Why these two orders of mammals? Why not other orders such as rodents, lagomorphs, shrews, and bats? In fact, there are plenty of extensive datasets for these (and other) orders. For example, see Mace et al (1981) J. Zool. 193:333-354, which presents brain size data for 261 species of terrestrial small mammals, and Hutcheon et al. (2002) Brain Behavior Evolution 60:165-180 for 63 species of bats. I would have thought that a comparative approach across the entire class Mammalia would have been more fruitful than simply presenting data on primates and (incongruously) carnivores. The authors make no attempt to justify their selection of mammalian orders.<br><br>Whilst we understand that brain data are available for more species than which were included within the manuscript, we wanted to run analyses on a complete dataset with all covariates available for all species, as this enabled more robust analyses, especially when conducting model comparisons. We could access all the required covariates for primates and carnivores, which governed our choice. In addition, in efforts to address the current confusion within the field regarding the proposed selection pressures responsible for increased brain size, we chose to use both primate and carnivore data as these two groups have received considerable attention, and thus by drawing clarity

Powered by Editorial Manager® and ProduXion Manager® from Aries Systems Corporation

within these two groups, further groups can be studied using more appropriate methods/procedures. We have added wording to emphasise our reasoning for this choice.

2) The literature cited is not representative of the field. A good deal of previous work has been omitted from this ms, including the two papers mentioned in (1) above, as well as Harvey et al. (1980) PNAS 77:4387-4389 (this paper explicitly deals with primate brain sizes). And there are many more papers that deal with ecological correlates of brain sizes that have not been mentioned.

Additional citations have been added.

3) Although the manuscript is generally well written, there are some sections that are difficult to interpret and/or to follow. This is particularly true for the Methods section, which is often ambiguous or at least incomplete. See below for where more detail is needed.

Wording has been rephrased for added clarity.

4) There is no definition of what is meant by the different brain volumes that are presented in the ms. For example, how was "endocranial brain volume" measured? And was it measured in the same way in the different papers where this information was extracted and collated? If not, then how can we be sure that we are comparing like with like?

5) The same comment applies to "neocortex" and "cerebellum" volumes.

Definitions have been added for endocranial and regional brain volumes. When sourcing all whole and regional brain volumes these measurement methods were considered to ensure the data was comparable. In terms of the ECV data, sources were checked for comparability and common measurement techniques were found between studies. We further tried to minimise the risk of this problem by sourcing data from whole datasets e.g., DeCasien et al., 2019 where the information has been weighted to account for multiple methods. However, this was more difficult with the carnivore data where regional brain volume data was tricky to source.

6) Again, how was social cohesion measured? I can see that it was scored on a point system of 1 to 4, but what does it mean for a species to have a social cohesion of 1? or 2? etc.

Definition revised for greater clarity.

7) I found the ecological data simplistic and not at all credible. The authors will need to justify exactly what they mean by each of the ecological variables. And then, they will need to convince the reader that the ecological data are actually meaningful. I am happy to include "diet" (although "frugivore" or "omnivore" are diet categories rather than strictly speaking diet itself (and the authors actually refer to diet categories, but they don't explicitly make the distinction). But what do they mean by diet breadth? According to their definition it is: "dietary breadth was also used, estimated using the total number of food sources used by a species". But what are these "food sources"? Are they the number of species of plants/animals taken? If so, an insectivorous species will by definition have a wider breadth than a carnivorous one (because there are more species of insects than vertebrates). If "sources" refers to something else, then what is it? And then, once the definition has been clearly stated, how can we be sure that the different studies have scored "number of food sources" in the same way?

Definitions of dietary categories and dietary breadth revised for greater clarity. All dietary breadth data was taken from one source: Wilman et al., (2014) and is referred to in the manuscript.

8) I have even more issue with the number of habitats used by a species. Wider ranging species will use a greater number of habitats, so why didn't the authors correct for this? Or simply use distributional range size instead of number of habitats?

Powered by Editorial Manager® and ProduXion Manager® from Aries Systems Corporation

Whilst we understand and appreciate this point, it does not always follow that wider ranging species will always use a greater number of habitats. One species may have a large home range size but may only move within the same habitat type. What we instead aim to look at here is whether the type of habitat matters, thus, do species which navigate and confront multiple habitat types, have larger brains than those which only move within one or two habitat types? Or vice versa? We also use home range size to proxy habitat use.

9) The authors do not mention where they get their home range sizes from in the ms (although these are clearly mentioned in the supplementary material). I find it hard to believe that the various range sizes compiled by numerous authors will be directly comparable due to differences in techniques used to estimate home range. Furthermore, there is enormous amount of variation in home range size, which is partly (and only partly) attributable to sex and age. Using a single metric is hardly informative or convincing.

We did not want to mention the citations specifically within the manuscript due to the high number of citations. We agree with this point about transferability of the methods used to measure home range size. We did our best to reduce the number of sources due to this problem, however, due to limited data availability, the only way to retrieve home range size for all species was to use data from multiple studies. To minimise the issue highlighted, we chose to use hectares to measure home range size as this was the most prevalent method found. We converted all home range data collected to this metric. We agree a single metric is not always useful, which is why we used both habitat variability and home range size to proxy habitat use.

10) Statistical analysis. This entire section (lines 218 to 239) needs to be reworked and more detail provided. And unambiguous statements rephrased. I will make just a few examples (but these are not the only problems).

Wording has been rephrased for clarity.

11) Lines 219-220 "using residuals from a regression line". Regression of what on what? And exactly using what regression? Simple linear regression e.g. lm()? On log transformed or untransformed data?

Phrase removed as this aspect has been moved to supplementary methods. This regression analysis is discussed in full within that document… “Phylogenetic generalised least-squares regression analysis (PGLS) was used to regress log brain volume against log body mass”.

12) What is the encephalisation quotient and how was it calculated? In fact, the equation is presented a bit further down, so perhaps the authors just need to refer to this e.g. say something like "see below for equation".

Definition revised for greater clarity. This aspect – as mentioned above – has been moved to the supplementary methods.

13) Line 220. "The former method is often preferred...". But you can't use "former" when there are three methods presented. "Former" and "latter" can only be used when comparing two things.

Thank you for highlighting. Phrase removed.

14) Line 226. "...therefore we considered it prudent to use both methods in the analyses...". Which two methods are being referred to? Because the authors have mentioned three methods (which have even been numbered).

Phrase removed.

15) Please provide a basic description of "Phylogenetic generalised least-squares regression analysis" and how it differs from typical GLMs.

Definition revised to provide greater clarity.

Powered by Editorial Manager® and ProduXion Manager® from Aries Systems Corporation

16) VIF was used to check for collinearity (which is good). But what does it mean "almost all scores" were below 5. Which variables were autocorrelated? And were any removed from the analyses, as a result of this?

Almost all VIF scores produced were below 5, however there were a few outliers. For example, body mass and weaning age produced scores of 7.25 and 5.93, when inputted into the primate endocranial model. Whilst moderately high, we chose to retain all variables within the statistical models, as the scores were only found in a few models and were still considerably low. Thus, no variables were removed from the analyses. VIF scores were also checked when rerunning analyses, specifically when using the 'rest of brain' regional volume technique, with no scores produced of concern.
This sentence has been updated to provide greater clarity.

17) Possible limitations. I find this paragraph difficult to accept. The authors are well aware that any models with AICs within 2 points are not "statistically different". Then how can they justify their approach? To me, this is the weakest aspect of the ms, because it affects all of their interpretations. There must be better ways of dealing with this. For example, list all competing models, and then count the number of times a particular variable (e.g. social cohesion) appears in the top models? This may make the results much more difficult to interpret, but this may be because there really is no simple and easy answer to the question that they are asking. Simplifying a complex problem with incorrect statistics is not acceptable.

We appreciate this comment. We agree this was a weak point in the analyses. To address this highlighted shortcoming, rather than just choosing the model with the absolute lowest score, we have now adopted the approach of presenting and discussing the results of all the 'best fit' models, which usually included a subset of models (simply, all the models within 2 points of the absolute lowest model). We have also rerun the analysis using BIC rather than AIC, in acknowledgement of this scoring system being more conservative.

Reviewer #2
•Line 33: See my comment in the Discussion section on the use of "counterbalancing".

Wording rephrased.

•There is a critical part currently missing this section, which is an explicit discussion of how this study is different from the many previous analyses of brain ~ socioecology relationships (e.g., inclusion of more variables, updated phylogeny, higher individual/species sample sizes)?

Thank you for this comment, we agree this was lacking in the manuscript. Introduction has been updated with this discussion.

•Line 75: The importance of pair-bondedness to brain size evolution was also discussed in other papers, which should be cited here (Schillaci 2006, 2008; MacLean et al. 2009).
•Line 83: This reference is only for carnivores – please add a reference for primates.

Citations added.

•Paragraph starting with Line 90:
oI think a discussion of issues with relative brain size measures is important, however, I don't think it warrants using measures that have been previously established as inappropriate (i.e., residuals, EQ).
•Lines 141-144: Again, it is unnecessary to include analyses using EQ or brain size residuals.
•Lines 218-220: Again, it is unnecessary to include analyses using EQ or brain size residuals.
•Paragraph starting with Line 467: As previously mentioned, previous studies have demonstrated that the use of EQ or residuals is inappropriate, so I think this paragraph and the relevant results are unnecessary and make the overall findings harder to

follow.

We appreciate that these methods have previously been suggested to be inappropriate for measuring the relationship between brain size and body mass. We feel it is necessary to further address this problem, however, especially considering we are using updated data, updated statistical analysis, more variables and an updated phylogenetic tree. After considering this point, we decided to move the results produced using the methods of concern (i.e., residuals, EQ) to the supplementary material and these will no longer be discussed in the main manuscript. This moves the focus away from those methods, but still allows the comparison between methods which may be useful to some readers.

oThe findings from the most recent study on brain ~ body size evolution (Smears et al. 2021) should be considered/discussed here.
oFreckleton's (2009) "seven deadly sins of comparative analysis" should be mentioned here, as it includes a discussion on why it is inappropriate to use residuals as outcome variables in regression models.
oLines 105-107 – Papers on lag between primate brain and body size should be mentioned here (e.g., Deaner and Nunn 1999).

Thank you. Citations added.

oLine 108: It is unclear what "over statistically controlled methods" means here.

Wording rephrased.

oLine 109: How and why does van Schaik et al. (2021) specifically demonstrate that EQ is inappropriate? The authors should elaborate a bit here.

Some elaboration has been added, as recommended.

•Paragraph starting with Line 111:
oHow would social and ecological variables specifically relate to neocortical and cerebellar functions?
•Increased brain size is the result of selection on specific abilities and related neural systems. Accordingly, at some point in this Introduction, I would appreciate a brief but explicit discussion of this (e.g., why might frugivory require greater visual information processing? Given that a large proportion of the brain is neocortex, and a large proportion of the neocortex is comprised of visual information processing areas, might this explain the link between something like frugivory and overall brain size?)

These points are now discussed.

oI think it would be appropriate to discuss Powell et al. (2019) here (currently only mentioned in the Discussion).

Powell et al., (2019) has been discussed further in the methods section.

•Line 126: What kind of "models"?

Sentence has been elaborated upon.

•Line 155: Please add sample sizes for the neocortex and cerebellum.

Sample sizes updated.

•Lines 157-161: This is Introduction material and should be removed from the Methods.
•Paragraph starting with Line 163: It might be useful to include some of this in the Introduction, since readers have any background surrounding issues with various "social complexity" measures.
•All descriptions of the links between socioecological variables and selection for cognitive abilities would be more appropriate in the Introduction.

These sections have been moved to the introduction.

Powered by Editorial Manager® and ProduXion Manager® from Aries Systems Corporation

•Lines 171-174: What were levels 2 and 3? How were pairbonded species or those that only sleep in pairs categorized? These levels need more explanation, especially since this “social cohesion” proxy was included in many best fit models in the Results.

Agreed. Definition revised for greater clarity.

•Lines 196-197: Diet imposes both temporal and spatial cognitive demands, so I suggest re-wording this.

•Lines 200-203: The authors appear to be suggesting that certain life history variables are drivers of evolutionary changes in brain size. I suggest altering the language here to mimic that in Lines 421-424.

Sentences rephrased for clarity.

•Paragraph staring with Line 200: This section is missing a discussion of ideas that the relationship between brain size and lifespan is driven by maternal investment and between specific brain regions and developmental periods (see e.g., Barton et al. 2011; Powell et al. 2019)

This point has been discussed.

•Lines 238-239: Why was body mass used as the covariate for the neocortex and cerebellum models? Many other papers have used brain size (with the brain region of interest removed) or medulla size as a covariate. This decision should be justified in the text or analyses should be re-run using a brain size measure.

Thank you for this comment, we agree that this method needed to be altered. Neocortex and cerebellum size were recalculated using endocranial volume minus the brain region of interest. Analyses were re-run using this brain size measure. The method (brain transformations) section has been updated to reflect this change.

•Model comparisons section:

oThis section as written is unclear – were the best fit models within Models 1-4 first identified, and then combined to make Model 5?

oIn any case, I do not think this approach is appropriate since it may, in some cases, force the inclusion of low information variables into the “combined” model. It would be more appropriate to create models that include all combinations of all predictor variables, compare these models using information criterion (I suggest using BIC since it is more conservative), and then select the best fit model or subset of models (e.g., all models with dBIC<2) to present detailed results.

Models one to four contained all combinations of the predictor variables, specifically looking at 1) social, 2) ecological, 3) social & ecological and 4) life history. Then usually models 3 and 4 were combined to determine whether incorporating the models together produced a better information criterion score. I say usually because sometimes incorporating social variables did not improve the score, therefore models 2 and 4 were combined instead. This combined model was also compared against a model including all variables together. We chose to use this ‘combined’ model because it would take too much time to try every combination of the 11 variables, therefore we thought by combining best fit models, this would bypass this problem and produce superior models. We appreciate your comment about the inclusion of low information variables, and it is definitely something we considered. After your suggestion, to better address the issue, the analyses have been re-run using BIC instead of AIC, due to the fact it is more conservative and would reduce the likelihood of low information variables being included. We also chose to present the results of the ‘best fit’ models, which was usually a subset of models (presenting all models within dBIC<2 of the absolute lowest model).

•Lines 260-261: The meaning of “presently, and subsequently” is unclear.

Phrase removed for clarity.

•This section is a bit difficult to follow as written. I suggest, within each section, more clearing separating/identifying the different groups of results. I think it would be most

appropriate to first discuss results using the information criterion (i.e., tell the readers which variables are included in the best fit models) and then the frequentist results (i.e., tell the readers which coefficient estimates within the best fit model are “significant” and the direction of the relationship)?

Thank you for this comment, we agree and the results section has been rewritten to allow greater clarity.

•Table 2: The diet category results (DFrug, DOmni) only demonstrate differences between these dietary groups (frugivory and omnivory) and folivory. This needs to be explicitly stated in the relevant areas of the results section. In addition, models should be run with the levels switched so that potential differences between frugivory and omnivory can also be tested.

Thank you for this comment, we agree that this needed highlighting. This has now been explicitly stated in the primate results section. In addition, as suggested, models were run with the levels switched, to identify any potential differences between frugivory and omnivory. This was checked on all ‘best fit’ models where diet was included, thus, on both the primate neocortex and cerebellum combined models. To do this, primate regional volume data was used, with linear regression models implemented, using the same combination of variables seen in the combined models (Neo ~ D + HR + ML + WA, Cere ~ D + HR + ML + WA).

Just included for your information…
Looking at primate neocortex data, when folivory was used as the baseline, negative significant associations were found with both omnivory and frugivory. However, when frugivory was used as the baseline, a positive association was found with folivory, whereas a negative association was found with omnivory. When omnivory was used as the baseline, positive associations were found with both frugivory and folivory. Thus, folivores appear to have larger neocortex volumes when compared to those with frugivorous and omnivorous diets, and this statement holds when the levels are switched (frugivorous and omnivorous species have smaller neocortex volumes when compared to those with a folivorous diet). However, frugivores appear to have larger neocortex volumes when compared to omnivores, and again, this statement holds when the levels are switched (omnivorous species have smaller neocortex sizes when compared to frugivorous species).

Looking at primate cerebellum data, the results are similar; both folivorous and frugivorous species appear to have larger cerebellar volumes when compared to those with an omnivorous diet, with this statement holding when the levels are switched (omnivorous species have smaller cerebellum volumes when compared to those with folivorous and frugivorous diets). However, there appears to be no discernible difference between folivorous and frugivorous species in terms of cerebellum volume.

•Lines 287-288 and 303-304: Table 2 includes results from best fit models only – it would be appropriate to also mention Table 1.

Table 1 has also been mentioned.

•Lines 288-289: Diet is not included in the best fit model for ECV in Table 1, so I am a bit confused about the claim that diet is positively associated with all brain measures.

What we meant by this sentence was that diet as a whole (dietary categories or dietary breadth) was associated with all brain measures. We agree this should have been better worded. This sentence has been removed, however, following the recommendation to no longer discuss the different brain measures in the main manuscript.

•Paragraph starting in Line 345: The home range results for the neocortex are not mentioned.

Thank you for pointing this out. We have now ensured all results are now appropriately discussed.

Powered by Editorial Manager® and ProduXion Manager® from Aries Systems Corporation

	•Lines 383-385: The finding that habitat variability is negatively correlated with relative brain size should be discussed in terms of previous work demonstrating a negative impact of seasonality on brain size (e.g., van Woerden et al. 2010).<br><br>This correlation is no longer found after rerunning statistical analyses so has been removed.<br><br>•Lines 409-410: This is not true. Powell et al. (2019) found correlations between specific brain regions (neocortex) and gestation length. Other regions were correlated with other developmental periods (e.g., cerebellum and juvenile period).<br><br>Sentence updated to reflect this point.<br><br>•Line 421: What does "counterbalance" mean? It sounds as if animals are actively participating in the evolution of these traits. Can the authors elaborate on how specific selection mechanisms would drive this "counterbalancing"?<br><br>Sentence updated to reflect this point.<br><br>•Lines 426-427: This sentence makes it seem that diet category is included in the best fit models for carnivores, which is not the case. I suggest removing the sentence.<br><br>Sentence removed as recommended.<br><br>•Lines 443-446: Sociality is not included in any of the best fit models of relative brain size, so this sentence is misleading as written.<br><br>Sentence changed following reanalysis of data.<br><br>•Lines 445-457: I would remove this sentence since the cerebellum is showing opposite trends across groups.<br><br>Sentence removed.
**Additional Information:**	
**Question**	**Response**
**Financial Disclosure**<br><br>Enter a financial disclosure statement that describes the sources of funding for the work included in this submission. Review the submission guidelines for detailed requirements. View published research articles from *PLOS ONE* for specific examples.<br><br>This statement is required for submission and **will appear in the published article** if the submission is accepted. Please make sure it is accurate.	The author(s) received no specific funding for this work.

Powered by Editorial Manager® and ProduXion Manager® from Aries Systems Corporation

**Unfunded studies**<br>Enter: *The author(s) received no specific funding for this work.*<br><br>**Funded studies**<br>Enter a statement with the following details:<br>• Initials of the authors who received each award<br>• Grant numbers awarded to each author<br>• The full name of each funder<br>• URL of each funder website<br>• Did the sponsors or funders play any role in the study design, data collection and analysis, decision to publish, or preparation of the manuscript?<br>• **NO** - Include this sentence at the end of your statement: *The funders had no role in study design, data collection and analysis, decision to publish, or preparation of the manuscript.*<br>• **YES** - Specify the role(s) played.<br><br>* typeset	
**Competing Interests**<br><br>Use the instructions below to enter a competing interest statement for this submission. On behalf of all authors, disclose any competing interests that could be perceived to bias this work—acknowledging all financial support and any other relevant financial or non-financial competing interests.<br><br>This statement is required for submission and **will appear in the published article** if the submission is accepted. Please make sure it is accurate and that any funding sources listed in your Funding Information later in the submission form are also declared in your Financial Disclosure statement.<br><br>View published research articles from *PLOS ONE* for specific examples.	The authors have declared that no competing interests exist.

**NO authors have competing interests**<br><br>Enter: *The authors have declared that no competing interests exist.*<br><br>**Authors with competing interests**<br><br>Enter competing interest details beginning with this statement:<br><br>*I have read the journal's policy and the authors of this manuscript have the following competing interests: [insert competing interests here]*<br><br>* typeset	
**Ethics Statement**<br><br>Enter an ethics statement for this submission. This statement is required if the study involved:<br><br>• Human participants<br>• Human specimens or tissue<br>• Vertebrate animals or cephalopods<br>• Vertebrate embryos or tissues<br>• Field research<br><br>Write "N/A" if the submission does not require an ethics statement.<br><br>General guidance is provided below. Consult the submission guidelines for detailed instructions. **Make sure that all information entered here is included in the Methods section of the manuscript.**	N/A

**Format for specific study types**<br><br>**Human Subject Research (involving human participants and/or tissue)**<br>• Give the name of the institutional review board or ethics committee that approved the study<br>• Include the approval number and/or a statement indicating approval of this research<br>• Indicate the form of consent obtained (written/oral) or the reason that consent was not obtained (e.g. the data were analyzed anonymously)<br><br>**Animal Research (involving vertebrate animals, embryos or tissues)**<br>• Provide the name of the Institutional Animal Care and Use Committee (IACUC) or other relevant ethics board that reviewed the study protocol, and indicate whether they approved this research or granted a formal waiver of ethical approval<br>• Include an approval number if one was obtained<br>• If the study involved *non-human primates*, add *additional details* about animal welfare and steps taken to ameliorate suffering<br>• If anesthesia, euthanasia, or any kind of animal sacrifice is part of the study, include briefly which substances and/or methods were applied<br><br>**Field Research**<br><br>Include the following details if this study involves the collection of plant, animal, or other materials from a natural setting:<br>• Field permit number<br>• Name of the institution or relevant body that granted permission	
**Data Availability**<br><br>Authors are required to make all data underlying the findings described fully available, without restriction, and from the time of publication. PLOS allows rare exceptions to address legal and ethical concerns. See the PLOS Data Policy and FAQ for detailed information.	Yes - all data are fully available without restriction

A Data Availability Statement describing where the data can be found is required at submission. Your answers to this question constitute the Data Availability Statement and **will be published in the article**, if accepted.<br><br>**Important:** Stating 'data available on request from the author' is not sufficient. If your data are only available upon request, select 'No' for the first question and explain your exceptional situation in the text box.<br><br>Do the authors confirm that all data underlying the findings described in their manuscript are fully available without restriction?	
**Describe where the data may be found in full sentences. If you are copying our sample text, replace any instances of XXX with the appropriate details.**<br><br>• If the data are **held or will be held in a public repository**, include URLs, accession numbers or DOIs. If this information will only be available after acceptance, indicate this by ticking the box below. For example: *All XXX files are available from the XXX database (accession number(s) XXX, XXX.*).<br>• If the data are all contained **within the manuscript and/or Supporting Information files**, enter the following: *All relevant data are within the manuscript and its Supporting Information files.*<br>• If neither of these applies but you are able to provide **details of access elsewhere**, with or without limitations, please do so. For example:<br><br>*Data cannot be shared publicly because of [XXX]. Data are available from the XXX Institutional Data Access / Ethics Committee (contact via XXX) for researchers who meet the criteria for access to confidential data.*<br><br>*The data underlying the results presented in the study are available from (include the name of the third party*	All relevant data are within the manuscript and its Supporting Information files.

and contact information or URL).<br>• This text is appropriate if the data are owned by a third party and authors do not have permission to share the data.<br><br>* typeset	
Additional data availability information:	

Powered by Editorial Manager® and ProduXion Manager® from Aries Systems Corporation

Manuscript
Click here to access/download;Manuscript;Manuscript_HRC_SOH_SH.docx

Why big brains? A comparison of models for both primate and carnivore brain size evolution

Helen R Chambers[1*], Sandra A Heldstab[2], Sean J O'Hara[1]

[1]School of Science, Engineering & Environment, University of Salford, Salford, Greater Manchester, United Kingdom

[2]Department of Anthropology, University of Zurich, Zurich, Switzerland

*Corresponding author

Email: h.r.chambers@edu.salford.ac.uk (HRC)

Abstract

Despite decades of research, much uncertainty remains regarding the selection pressures responsible for brain size variation. Whilst the influential social brain hypothesis once garnered extensive support, more recent studies have failed to find support for a link between brain size and sociality. Instead, it appears there is now substantial evidence suggesting ecology better predicts brain size in both primates and carnivores. Here, different models of brain evolution were tested, and the relative importance of social, ecological, and life-history traits were assessed on both overall encephalisation and specific brain regions. In primates, evidence is found for consistent associations between brain size and ecological factors, particularly diet; however, evidence was also found advocating sociality as a selection pressure driving brain size. In carnivores, evidence suggests ecological variables, most notably home range size, are influencing brain size; whereas, no support is found for the social brain hypothesis, perhaps reflecting the fact sociality appears to be limited to a select few taxa. Life-history associations reveal complex selection mechanisms to be counterbalancing the costs associated with expensive brain tissue through extended developmental periods, reduced fertility, and extended maximum lifespan. Future studies should give careful consideration of the methods chosen for measuring brain size, investigate both whole brain and specific brain regions where possible, and look to integrate multiple variables, thus fully capturing all of the potential factors influencing brain size.

Key words: brain size, primates, carnivores, sociality, diet, encephalisation

Introduction

Brain size varies considerably amongst mammals; substantial variation is seen among primates, where brain size varies almost a thousand-fold across the order [1]. The adaptive value of such variation has come under extensive scrutiny over the past few decades and yet despite considerable research effort, much uncertainty remains regarding the selection pressures responsible.

Frequently proposed to explain variation in brain size are factors related to the physical environment, such as diet and home range size, as well as factors related to the social environment, such as group size and pair-bondedness. Ecological hypotheses mainly involve investigating the cognitive demands associated with foraging [2-7], as foraging is considered mentally demanding due to the pressure of managing, processing and remembering spatial and temporal information about resource availability [8-12]. Additionally, differing home range size is of interest to researchers due to the supposed cognitive demands imposed by larger home ranges, such as processing requirements of navigating spatially-complex information, especially in terms of food availability, location and distribution [9, 13-15]. This has resulted in many studies investigating the cumulative effects of the physical environment on encephalisation, with a specific interest in diet [16-20], home range [13, 14], foraging techniques [12, 21-23] and behavioural responses in a fluctuating environment [24].

In contrast to ecological hypotheses, the social brain hypothesis (SBH) suggests sociality − specifically the cognitive demands of tracking, negotiating and maintaining social relationships − to be the main driving force behind variation in primate brain sizes [25-27]. The study of primates lends credence to this hypothesis, with brain size found to correlate

with many social proxies, such as social group size [28], tactical deception [29] and grooming clique size [30]. Evidence has since not been limited to studies of the primate lineage, with corroboration coming from research on spotted hyenas [31, 32] as well as other carnivorans [33-35], ungulates [36, 37], birds [38-40], and some fish species [41-43]. The focal point of much of the early work investigating sociality was social group size, due to the information-processing demands group of increasing sizes are thought to incur [26]. However, the use of this proxy for measuring social complexity has been criticised [44] and instead, focus has shifted to the consequences of varying levels of relationship complexity [45], and toward investigating the influence of pair-bondedness [27, 46-48]. This developed from the proposition that relationship quality [45, 49] connotes cognitive complexity.

Despite the hypothesis receiving considerable support in the past, more recent investigations have failed to find statistical support for a link between brain size and sociality [14, 19, 20, 50, 51]. Instead, it appears there is now substantial, strong, phylogenetically-corrected comparative data reinforcing the assertion that diet better predicts brain size in both primates and carnivores [14, 20, 52]. In addition, the obvious exceptions to the SBH, taxa ~~who~~ possess large brains but ~~who~~ are not considered social, suggest factors other than sociality may be influencing brain size [19, 53, 54]. For example, if sociality is to be accepted as the causal agent for increased encephalisation in mammals, it should be widespread across bears and musteloids, ~~who~~ show similar encephalisation increases to Canidae [55].

A further problem to have dogged comparative analyses of brain evolution is deciding on the correct brain measure. Whilst most studies tend to focus on whole brain size, even this can become an arduous task since there is little clarity in the literature regarding the most appropriate body size correction factor, making decisions on the correct method of choice

challenging. Typically, cognitive abilities are estimated using relative brain size, by taking residuals from a regression curve or calculating encephalisation quotients [56, 57]. This became the method of choice when brain and body size were found to be tightly coupled allometrically across vertebrates; therefore, accounting for this allometric relationship became of great importance [35, 58]. However, the use of relative brain size and encephalisation quotients is not without criticism; for example, using residuals as data points in regression models has been discouraged, as the estimates produced are thought to be biased, which influences subsequent analyses [59, 60]. Encephalisation quotients possibly reflect the result of recent decreases or increases in body size [61], evidence for such was uncovered by Swanson et al. [19]. They found carnivore brain size to lag behind body size over evolutionary time, therefore hinting that the use of brain estimates may be a poor representation of carnivore brain size. However, no evidence for a lag is found for primates [62], suggesting a taxonomic difference for this group. Alongside this, the prevalent use of relative brain size is thought to possibly hide other evolutionary pathways which may be influencing adaptations in body mass [63]. For example, a recent analysis of mammalian brain size found the brain-to-body relationship to uncover more than just selection on brain size, indicating relative brain size measures are not accurately capturing brain size variation [64]. Thus, van Schaik et al., [65] suggest the use of encephalisation quotients should be avoided in future studies, as EQs repeatedly fail to accurately predict brain size, and thus, varying levels of cognitive ability. For example, Deaner et al., [57] found absolute brain size measures, over statistically produced methods i.e., residuals, to be the best predictors of primate cognitive abilities.

Alongside the use of total brain size, particular emphasis has been put on specific brain regions in recent years. The social brain hypothesis suggests the neocortex is the brain

structure of interest, with primates' large brains thought to be mainly the consequence of a dramatic increase in neocortical volume [66-68]. The neocortex is thought foremost responsible for the processing of more demanding cognitive and social skills [69, 70] associated with intelligent and flexible behaviour [61]. Neocortical enlargement in primates is thought to be partly due to selection on visual mechanisms [71] which is important for frugivorous species, for example when needing to distinguish between fruits of different colours [72-74] or when manipulating small fruit and seeds that require fine motor coordination [75]. Alternatively, these visual mechanisms are thought to be important for processing complex and rapid social interactions, including understanding facial expressions, gaze direction and posture [76], suggesting that neocortical modifications associated with complex social lives primarily involve areas specialised for visual processing of social information [77]. In primates, the neocortex constitutes a substantial portion of the brain [66, 67] and a large proportion of the neocortex is comprised of visual information processing areas [71, 78, 79], which is thought to explain links found between frugivory and brain size (see [20]), as well as social group size and neocortex volume (see [1, 71]). Alongside research into the neocortex, attention is focused on the cerebellum and its importance. The cerebellum was found to co-evolve with the neocortex [61], with a significant correlation found between these two brain regions [80]. Increased cerebellar volume is suggested to allow increased processing capacity, in terms of enhanced motor abilities and manipulative abilities [81, 82]. For example, in primates positive correlations are found between cerebellum volume and extractive foraging techniques [1], as well as the presence of neural activation in the cerebellum during tool use in monkeys [83].This highlights the influential role played by the cerebellum in technical intelligence [84]. Alongside this, the cerebellum is thought to be important in social intelligence [1],

particularly in terms of the links between sensory-motor control and social interactions and understanding [85, 86]. Indeed, it is now thought the expansion of the cortico-cerebellar system is the primary driver of brain expansion in anthropoid primates [87], suggesting the increased behavioural complexity in mammals could be partly explained by selection on the cerebellum [88]. So much so, that Fernandes et al., [89] found residual cerebellar size to be the most appropriate proxy when compared to a measure of general intelligence; as cerebellar models produced the most similar model fit results when compared to those produced using a measure of general intelligence.

Here, using data aggregated from the literature the relative importance of social, ecological and life history traits are assessed on both overall encephalisation and specific brain regions, and different models of brain size evolution are tested. Considerable attention has been paid to primate brain evolution (e.g., [14, 20, 90, 91]) perhaps since there are substantial data available on this taxonomic group making comparative tests easy to implement. Likewise, carnivorans are also now receiving attention (e.g., [19, 88, 92, 93]) since variation in their brain and body size, and ranging social and physical environments, makes them excellent models for these tests too. Indeed, most of the literature surrounding brain size hypotheses is based on analyses of these two groups.

One aim here, therefore, is to provide greater clarity within these two groups. Integrating predictors into a framework which allow the assessment of multiple hypotheses simultaneously has become increasingly important for tests of brain evolution [94, 95]. Therefore, phylogenetically-corrected generalised least squares (PGLS) models are used here to account for shared evolutionary history, whilst assessing the potential variables influencing encephalisation. We use a recently updated phylogenetic tree to ensure phylogenetic relationships are contemporary. Further, the inclusion of multiple variables

allows the comparison of multiple hypotheses, as well as models of varying complexity. While brain data are available for more taxa than are included in our dataset, we found some limitations on the completeness of the necessary covariate data. We present here our analyses of two orders where complete datasets with all covariates are available for all species, ensuring the most robust model comparisons.

Methods

Data Collection

Brain data

Endocranial volume (ECV) and body mass data for primates (n = 83) and carnivores (n = 85) were compiled from multiple sources (see supplementary material). Volumes were matched for species composition and predictor variables, and whilst this resulted in smaller sample sizes when compared to available brain data, in doing so it provided a complete dataset with all covariates available for all species, better enabling robust analyses. ECV data were preferred over brain mass data since it is thought ECV provides a more reliable estimate of brain size, due to the influence of preservation techniques on brain mass [96]. The standard technique for estimation of ECV is through filling the cranium with beads (or similar), which is then measured using a graduated cylinder or by weighing the beads and converting the weight to volume [96]. Neocortex and cerebellum volumes were also collated, where available, for both primates (Neo = 52, Cere = 49) and carnivores (Neo = 44, Cere = 38). Regional brain volumes are commonly measured using one of two different techniques: virtual endocasts (e.g., [19]) or physical sectioning of the individual brain volumes using

paraffin and staining substances (e.g., [97]). When sourcing whole and regional brain volumes these measurement methods were considered to ensure the data were comparable; for example, all ECV data sources used common measurement techniques (as described above) making the whole brain data comparable across multiple studies.

Social data

Both social group size and social cohesion data were collected for primates and carnivores. Group size – based on the simple principle that as group size increases the information-processing demands [26] and corresponding internal structures [98, 99] should also increase − became perhaps the most commonly used proxy for social complexity. Despite this, the use of this proxy has been criticised as it is often considered crude, weak, and not always relevant [44]. Greater attention is now paid to differing levels of relationship complexity [45] often indicated through the presence of pair-bonds [27, 34, 100]. Therefore, to ensure the influence of sociality was fully captured, alongside group size, a social cohesion proxy was used: a categorisation system ranging from 1) being primarily solitary living aside from breeding seasons, 2) pair-living, 3) fission-fusion societies, to 4) being obligatorily social (e.g., [91, 101]). This index aims to better encapsulate sociality, rather than relying solely on group size numbers.

Ecological data

Four ecological variables were chosen for analysis: dietary categories, dietary breadth, habitat variability and home range size. Dietary categories were assigned following previous designations in the published literature (see supplementary material for sources) and included six different categories: carnivorous, herbivorous, piscivorous, folivorous, frugivorous and omnivorous. Alongside this traditional classification system, dietary breadth

was also used, estimated using the total number of food sources used by a species, with data taken from [102]. This included a total of 10 different food types: invertebrates, mammals and birds, reptiles, fish, unknown vertebrates, scavenge, fruit, nectar, seed or other plant material, marked either as absent (0) or present (1). For this dataset, this resulted in a dietary breadth scale of one to six. Habitat variability, another ecological measure, was formed using data from the IUCN Red List [103], based on the total number of habitat-types used by a species, following the same habitat classification system used in the IUCN Red List. Additionally, home range size data were collected. By including variables related both to diet and habitat, it allowed greater incorporation of possible variables within the physical environment affecting brain size.

Life-history data

Life-history variables have been found to be critical in counterbalancing the costs of increased brain size and facilitating the growth of large brains [104]. In fact, they appear to be influencing the potential adaptive pathways available to a species [94], for example in terms of balancing shifting developmental and maturation periods. Developmental costs are also thought to influence correlations between specific primate brain structures and life history variables, with the neocortex most strongly correlated with gestation length, and the cerebellum with juvenile period length, suggesting that these brain regions exhibit distinct life-history correlates which concur with their unique developmental trajectories [105]. Hence, it was necessary to include certain life history variables in the analysis to further understand how life-history characteristics potentially act as a filter [104, 106] for the production of large brains. Gestation length was chosen as it has received considerable attention and is thought to be of great importance in bypassing the constraints of

precociality in mammals and facilitating brain growth [107]. Maximum lifespan was included as there is substantial support that encephalisation is correlated with extended longevity [104], especially in primates [108, 109]. The relationship found between brain size and lifespan is thought to be driven primarily by maternal investment, with subsequent correlations found between specific brain regions and developmental periods, reflecting this brain size-lifespan association (see [105, 110]. Ultimately encephalisation has been found to correlate with expansion of most developmental life history stages, including an extended reproductive lifespan [111]. Therefore, data on age at first reproduction, weaning and fertility (measured as number of offspring per year) were added to our dataset (see supplementary material for sources).

Statistical Analyses

Brain transformations

Whole brain volumes were incorporated in analyses by simple incorporation of log ECV volume with log body mass included as a covariate. This method is often preferred over the use of residuals as variables in ecological datasets often covary thereby producing biased parameter estimates when calculating residuals [59]. Including body mass as a covariate in the model avoids this problem, controls for its effect on brain volume, as well as potentially controlling for any effects body mass may have on other variables included. Regional brain volumes were incorporated in analyses by simple incorporation of log ROB (rest of brain) volume. To calculate ROB volume for both the neocortex and cerebellum, a calculation was performed: whole brain volume minus the region volume of interest. This method has been previously implemented and proved useful in measuring relative regional brain volumes (e.g., [91]). Further analyses were also conducted in order to test how uniform results were

when using different brain size measures. The results of these analyses are displayed and discussed in the supplementary material.

PGLS analysis

All statistical analyses were performed using R 4.0.1, using the 'caper', 'ape' and 'geiger' packages. Phylogenetic generalised least-squares (PGLS) regression analysis was used to identify those variables influencing whole and regional brain evolution, whilst avoiding the problem of phylogenetic non-independence. This technique differs from standard generalised least squares analysis, as it uses knowledge of phylogenetic relationships or relatedness to produce estimates of the expected covariance across species [112]. Pagel's λ was estimated by maximum likelihood. The tree used for all phylogenetic analyses was that of Upham et al's [113]. All continuous variables, brain volumes and body mass were log transformed prior to analysis to satisfy the assumption of normality. Variance Inflation Factor (VIF) scores were used to check for the presence of multicollinearity, with almost all scores found to be below 5, and no scores above 7. There were no scores produced which highlighted concern, and thus, all socioecological and life-history variables were retained for analysis (see supplementary material).

Model comparisons

A series of PGLS models were implemented which varied in complexity, including 1) social, 2) ecological, 3) social and ecological, 4) life history and 5) variables of interest. Models one to four included all possible combinations of the selected variables; for example, the social model included i) group size, ii) social cohesion, iii) group size and social cohesion. BIC (Bayesian Information Criterion) values of each model were then compared [114]. As lower BIC values indicate the presence of better fitting, more parsimonious models, the model

with the lowest BIC value was deemed to best explain the data, therefore considered preferrable and retained. BIC values were preferred over Akaike Information Criterion values because BIC resolves the problem of overfitting, by using a more conservative penalty for additional variables. Model number five was constructed using all variables previously highlighted of interest within the social, ecological, and life history models. This allowed us to compare the importance of social versus ecological models, as well as construct models including those variables that best explained the data. Once computed, model five was compared alongside the previous models, and those found to have the lowest BIC value were then considered the '*best fit*' models, which in some cases represents a subset of models (simply, any model within dBIC<2 of the lowest model). This is because BIC values with a difference of between 2 and 6 indicate moderate evidence that the model with the lower BIC provides a relatively better model fit, whilst greater than 6 indicates strong evidence for improved fit.

Results

Primates

The results from PGLS analysis on the primate data are shown in Table 1. Almost all models were highly significant. For most models λ was close to one, indicative of a Brownian motion model of trait evolution; however, certain neocortex models stand in contrast to this, with λ equal to zero, implying the data have no phylogenetic structure [84]. Combined models were preferred when investigating both whole and regional brain volumes, with significantly improved (equal or greater than two BIC units lower than another) BIC scores when combining variables indicated to be of importance in previous model iterations. When

comparing the influence of ecology versus sociality, ecological models were found to be preferable to social models, evidenced by the presence of significantly improved BIC scores.

Table 1. Phylogenetic generalised least-squares (PGLS) regression analyses examining the effects of social, ecological and life-history variables* on primate whole and regional brain volumes.

**Brain input**	**Overall model**	**Preferred model**	**BIC score**	***P***
**Endocranial volume**	Social	ECV ~ Mass + SC	-184.199	<0.001
	Ecological	ECV ~ Mass + DB	-190.8458	<0.001
	Social & Ecological	ECV ~ Mass + SC + DB	-192.0528	<0.001
	Life History	ECV ~ Mass + GL + ML + WA	-201.2257	<0.001
	**Combined**	**ECV ~ Mass + GS + DB + GL + ML + WA**	**-208.5244**	**<0.001**
	All	ECV ~ Mass + GS + SC + D + DB + HV + HR + GL + ML + F + FR + WA	-183.9911	<0.001
**Neocortex**	Social	Neo ~ SC	36.43372	<0.05
	Ecological	Neo ~ D + HR	20.04	<0.001
	Social & Ecological	Neo ~ SC + D + HR	23.04369	<0.001
	Life History	Neo ~ ML + WA	-9.507772	<0.001
	**Combined**	**Neo ~ D + HR + ML + WA**	**-17.54041**	**<0.001**
	All	Neo ~ GS + SC + D + DB + HV + HR + GL + ML + F + FR + WA	9.397628	<0.001
**Cerebellum**	Social	Cere ~ SC	26.55957	<0.05
	Ecological	Cere ~ D + HR	0.2775847	<0.001
	Social & Ecological	Cere ~ SC + D + HR	3.144599	<0.001
	Life History	Cere ~ ML + WA	-17.40863	<0.001
	**Combined**	**Cere ~ D + HR + ML + WA**	**-25.9437**	**<0.001**
	All	Cere ~ GS + SC + D + DB + HV + HR + GL + ML + F + FR + WA	-10.45452	<0.001

**GS = Group size, SC = Social cohesion, D = Diet, DB = Dietary breadth, HV = Habitat variability, HR = Home range, GL = Gestation length, ML = Maximum longevity, F = Fertility, FR = Age at first reproduction, WA = Weaning age.*

Overall encephalisation

The results of PGLS analysis on endocranial volume data are presented in Table 1, with the '*best fit*' models presented in Table 2. The variables which were indicated to be of

importance and included in the '*best fit*' endocranial volume models were: group size, dietary breadth, gestation length, maximum lifespan and weaning age. Also present in the subset of '*best fit*' models were: social cohesion and home range. After accounting for phylogeny, both group size and social cohesion were found to be positively associated with ECV (P <0.05). Although, social cohesion failed to reach significance in certain model iterations (P = 0.06). In terms of the ecological variables, dietary breadth was consistently associated with ECV (P <0.001); however, home range size failed to reach significance (P = 0.11). Three of the life-history variables were significantly associated with ECV: gestation length, maximum lifespan and weaning age (P <0.01).

Table 2. Phylogenetic generalised least-squares (PGLS) regression analyses examining the effects of social, ecological and life-history variables* on primate whole and regional brain volumes.

**Brain input**	**Preferred models**	**BIC score**	**Predictor**	**T**	***P***
**Endocranial volume**	ECV ~ Mass + GS + DB + GL + ML + WA	-208.5244	Intercept	-6.6214	<0.001***
			LogMass	18.9909	<0.001***
			GS	2.1248	**<0.05***
			DB	3.2392	**<0.01****
			LogGL	2.8949	**<0.01****
			LogML	3.0356	**<0.01****
			LogWA	3.3570	**<0.01****
	ECV ~ Mass + SC + DB + GL + ML + WA	<2	Intercept	-6.5280	<0.001***
			LogMass	18.8287	<0.001***
			SC	2.0765	**<0.05***
			DB	3.5498	**<0.001*****
			LogGL	2.8406	**<0.01****
			LogML	2.7985	**<0.01****
			LogWA	3.2441	**<0.01****
	Mass + SC + DB + HR + GL + ML + WA	<2	Intercept	-6.6062	<0.001***
			LogMass	17.6895	<0.001***
			SC	1.9298	0.06
			DB	3.6480	**<0.001*****
			LogHR	1.6222	0.11
			LogGL	3.0146	**<0.01****

			LogML	2.7384	**<0.01****
			LogWA	3.0851	**<0.01****
**Neocortex**	Neo ~ D + HR + ML + WA	-17.54041	Intercept	6.0124	<0.001***
			DFrug	-2.1200	**<0.05***
			DOmni	-3.9187	**<0.001*****
			LogHR	3.2303	**<0.01****
			LogML	4.4548	**<0.001*****
			LogWA	6.4547	**<0.001*****
**Cerebellum**	Cere ~ D + HR + ML + WA	-25.9437	Intercept	7.4158	<0.001***
			DFrug	-1.5536	0.13
			DOmni	-3.0869	**<0.01****
			LogHR	4.2338	**<0.001*****
			LogML	3.0810	**<0.01****
			LogWA	5.8047	**<0.001*****
	Cere ~ D + HR + GL + ML + WA	<2	Intercept	1.2227	0.23
			DFrug	-1.0319	0.31
			DOmni	-2.7180	**<0.01****
			LogHR	4.4768	**<0.001*****
			LogGL	1.8597	0.07
			LogML	2.4562	**<0.05***
			LogWA	3.6953	**<0.001*****

*GS = Group size, SC = Social cohesion, D = Diet, DB = Dietary breadth, HV = Habitat variability, HR = Home range, GL = Gestation length, ML = Maximum longevity, F = Fertility, FR = Age at first reproduction, WA = Weaning age.

Regional brain volumes

The results of PGLS analysis on the neocortex and cerebellum data are presented in Table 1, with the '*best fit*' models presented in Table 2. The variables which were indicated to be of importance and included within the '*best fit*' neocortex model were: diet, home range size, maximum lifespan and weaning age. After accounting for phylogeny, diet, specifically frugivory and omnivory were found to be negatively associated with neocortex volume (P <0.05, P <0.001). This is the result produced when a folivorous diet is used as the baseline category, therefore the dietary category results produced here only demonstrates differences between these dietary groups (frugivory and omnivory) and folivory. Alongside these associations, home range size was positively correlated with neocortex volume (P

<0.01). Similar to whole brain models, both maximum lifespan and weaning age were significantly associated with neocortex volume (P <0.001).

The variables which were indicated to be of importance and included in the '*best fit*' cerebellum models were: diet, home range size, maximum lifespan and weaning age. Also present within the subset of '*best fit*' models was: gestation length. After accounting for phylogeny, diet, specifically omnivory was found to be negatively associated with cerebellum volume (P <0.01). Frugivory failed to be significant (P = 0.13, P = 0.31). As above, this results when folivorous diet is used as the baseline category. Home range size was positively associated with cerebellum volume (P <0.001). Similar to previous life-history results, maximum lifespan and weaning age were significantly associated with cerebellum volume (P <0.01, P <0.001). Gestation length was close to being significantly correlated with cerebellum volume (P = 0.07).

Carnivores

The results of PGLS analysis on the carnivore data are presented Table 3. Almost all models were highly significant. Lambda was not consistent between the models, ranging from one to zero across the dataset. In terms of the '*best fit*' models, those producing the lowest BIC score (or any score within dBIC<2 of the lowest model), there was no significant difference between life history and combined models, and thus the results of all these models are discussed below. When comparing the influence of ecology versus sociality, ecological models were found to be preferable to social models when investigating regional brain volumes, evidenced by the presence of significantly improved BIC scores. However, this was

not the case in whole brain models, where there was no significant difference between the preferred social and ecological models.

Table 3. Phylogenetic generalised least-squares (PGLS) regression analyses examining the effects of social, ecological and life-history variables* on carnivoran whole and regional brain volumes.

**Brain input**	**Overall model**	**Preferred model**	**BIC score**	***P***
**Endocranial volume**	Social	ECV ~ Mass + GS	-137.3671	<0.001
	Ecological	ECV ~ Mass + HV	-138.8228	<0.001
	Social & Ecological	ECV ~ Mass + GS + HV	-135.0748	<0.001
	**Life History**	**ECV ~ Mass + F**	**-140.9778**	**<0.001**
	Combined	ECV ~ Mass + DB + F	-140.4778	<0.001
	All	ECV ~ Mass + GS + SC + D + DB + HV + HR + GL + ML + F + FR + WA	-106.9128	<0.001
**Neocortex**	Social	Neo ~ GS	71.58854	0.06425
	Ecological	Neo ~ HR	68.10774	<0.01
	Social & Ecological	Neo ~ GS + HR	70.20444	<0.01
	**Life History**	**Neo ~ FR**	**58.64386**	**<0.001**
	Combined	Neo ~ HR + FR	59.78632	<0.001
	All	Neo ~ GS + SC + D + DB + HV + HR + GL + ML + F + FR + WA	87.42208	<0.001
**Cerebellum**	**Social**	Cere ~ GS	35.60386	0.07056
	Ecological	Cere ~ HR	20.3267	<0.001
	Social & Ecological	Cere ~ GS + HR	22.22221	<0.001
	Life History	Cere ~ GL + ML + FR	4.668459	<0.001
	**Combined**	**Cere ~ HR + GL + ML + FR**	**3.803654**	**<0.001**
	All	Cere ~ GS + SC + D + DB + HV + HR + GL + ML + F + FR + WA	28.10051	<0.001

*GS = Group size, SC = Social cohesion, D = Diet, DB = Dietary breadth, HV = Habitat variability, HR = Home range, GL = Gestation length, ML = Maximum longevity, F = Fertility, FR = Age at first reproduction, WA = Weaning age.

Overall encephalisation

The results of PGLS analysis on endocranial volume data are presented in Table 3, with the '*best fit*' models shown in Table 4. The variables which were indicated to be of importance

and included within the '*best fit*' endocranial volume models were: fertility, dietary breadth, maximum longevity and age at first reproduction. After accounting for phylogeny, fertility was found to be negatively associated with ECV (P <0.05), with this being the only variable significantly associated with endocranial volume. For example, dietary breadth was close to being negatively associated with ECV, but fell short of significance (P = 0.05). In addition, both maximum lifespan and age at first reproduction, failed to reach significance (P = 0.08, P = 0.10).

Table 4. Phylogenetic generalised least-squares (PGLS) regression analyses examining the effects of social, ecological and life-history variables* on carnivoran whole and regional brain volumes.

Brain input	Preferred models	BIC score	Predictor	T	*P*
**Endocranial volume**	ECV ~ Mass + F	-140.9778	Intercept	-5.3678	<0.001***
			LogMass	25.7757	<0.001***
			LogF	-2.0993	**<0.05***
	ECV ~ Mass + DB + F	<2	Intercept	-4.4263	<0.001***
			LogMass	25.6777	<0.001***
			DB	-1.9622	0.05
			LogF	-2.4784	**<0.05***
	ECV ~ Mass + ML	<2	Intercept	-7.0336	<0.001***
			LogMass	24.0699	<0.001***
			LogML	1.7925	0.08
	ECV ~ Mass + FR	<2	Intercept	-6.0877	<0.001***
			LogMass	21.5774	<0.001***
			LogFR	1.6682	0.1
**Neocortex**	Neo ~ FR	58.64386	Intercept	35.4993	<0.001***
			LogFR	5.6022	**<0.001*** **
	Neo ~ ML + FR	<2	Intercept	3.3575	<0.01**
			LogML	1.3334	0.19
			LogFR	2.6229	**<0.05***
	Neo ~ HR + FR	<2	Intercept	17.222	<0.01**
			LogHR	1.856	0.07
			LogFR	3.786	**<0.001*** **
**Cerebellum**	Cere ~ HR + GL + ML + FR	3.803654	Intercept	1.8971	0.066599
			LogHR	2.0374	**<0.05***

		LogGL	2.0974	**<0.05***
		LogML	2.7665	**<0.01****
		LogFR	2.1567	**<0.05***
Cere ~ GL + ML + FR	<2	Intercept	2.0734	<0.05*
		LogGL	1.8730	0.07
		LogML	2.8402	**<0.01****
		LogFR	3.8113	**<0.001*****
Cere ~ ML + FR	<2	Intercept	5.9931	<0.001***
		LogML	3.1178	**<0.01****
		LogFR	4.9662	**<0.001*****
Cere ~ HR + ML + FR	<2	Intercept	5.9347	<0.001***
		LogHR	1.8137	0.08
		LogML	3.0414	**<0.01****
		LogFR	3.1242	**<0.01****
Cere ~ ML + FR + WA	<2	Intercept	4.7991	<0.001***
		LogML	2.7130	**<0.05***
		LogFR	4.4666	**<0.001*****
		LogWA	1.6954	0.1

*GS = Group size, SC = Social cohesion, D = Diet, DB = Dietary breadth, HV = Habitat variability, HR = Home range, GL = Gestation length, ML = Maximum longevity, F = Fertility, FR = Age at first reproduction, WA = Weaning age.

Regional brain volumes

The results of PGLS analysis on the neocortex and cerebellum data are presented in Table 3, with the '*best fit*' models shown in Table 4. The variables which were indicated to be of importance and included in the '*best fit*' neocortex models were: age at first reproduction, maximum lifespan and home range size. After accounting for phylogeny, age at first reproduction was found to be positively associated with neocortex ($P < 0.001$), with this being the only variable significantly associated with neocortex volume. For example, home range size was close to being positively associated with neocortex volume, but fell short of significance ($P = 0.07$). In addition, maximum lifespan failed to reach significance ($P = 0.19$).

The variables which were indicated to be of importance and included within the '*best fit*' cerebellum models were: home range size, gestation length, maximum lifespan and age at first reproduction. Also present within the subset of '*best fit*' models were: different iterations of the previously mentioned variables and weaning age. After accounting for phylogeny, home range size was found to be significantly associated with cerebellum volume ($P < 0.05$). Three of the life-history variables were significantly associated with cerebellum volume: gestation length, maximum lifespan and age at first reproduction ($P < 0.05$, $P < 0.01$, $P < 0.001$). Although, home range size and gestation length failed to reach significance in certain model iterations ($P = 0.08$, $P = 0.07$). Weaning age also failed to reach significance ($P = 0.10$).

Discussion

Applying robust statistical analyses, a recently updated phylogenetic tree, a comprehensive dataset and models of varying complexity, the correlates of brain size in primates and carnivores were reconsidered. Consistent associations were found between brain size and ecological variables in primates, thus highlighting the influence of ecology on encephalisation. However, support was also found for the prominent social brain hypothesis, specifically revealing evidence for a link between whole brain volumes and two measures of sociality. In carnivores, data suggest ecological variables shape brain size, suggesting alternative evolutionary patterns influencing carnivoran encephalisation. In both groups, life history variables appear crucial in counterbalancing the costs of producing and maintaining increased brain size, through extended developmental periods, reduced fertility and increased maximum lifespan.

Primates

Here, consistent with current literature, robust correlations were found between brain size and ecological variables. The most prominent of these were diet related, with dietary categories or dietary breadth appearing in all '*best fit*' models, for both whole brain and regional brain data. These findings are similar to those of DeCasien et al., [20] and Powell et al. [14], who found stronger and more consistent associations with ecological variables than those related to the social environment. Akin to the result of DeCasien et al. [20], support was found for omnivory, as well as frugivory, as correlates of brain size. However, in contrast to the literature, here the correlations between regional brain volumes and dietary categories, were negatively correlated. This perhaps reflects both the need to sustain the energetic cost of brain tissue (highlighted by [115]; [116]), as well as meeting the cognitive foraging challenges imposed by omnivorous and frugivorous diets [3]. In addition to the dietary categories, dietary breadth was significantly (positively) correlated with whole brain volumes, further reinforcing the proposition that diet influences brain size, whilst highlighting how useful this proxy can be in understanding how availability and variety of food sources can be important in setting the cognitive challenge. For example, MacLean et al. [50] also suggested dietary breadth to be an important ecological correlate, with greater cognitive flexibility allowing individuals to explore and exploit new food sources, as well as deploy extractive foraging techniques. Evidence for associations between regional brain volumes and home range size were also found, supporting the view of Powell et al. [14] in that certain dietary categories, such as frugivory, may covary with home range. Similar results were also found by Graber et al. [117].

In the past considerable support indicated that sociality was the major driver of encephalisation in primates. More recent works, however, contest this long-held viewpoint, failing to find support for a link between brain size and sociality measures [14, 19, 20, 50, 51]. Our findings, however, confirm support for the social brain hypothesis. Here, our models revealed evidence of a link between brain size and sociality in primates. This association was present only in the whole brain '*best fit*' models, with both variables reaching significance, indicating both increasing social group size and varying levels of social cohesion are influencing brain size in primates. Interestingly, use of the social cohesion proxy was often preferred when comparing models, thereby suggesting the use of this proxy is superior when testing multiple ecological and social variables simultaneously. The inference too is that there may be greater importance in relationship quality, over quantity, as suggested by past research into primate sociality and pair-bonds [34, 45, 49, 95, 118]. It is important to note however, that whilst there was support for this hypothesis, ecological models were preferrable over social ones and ecological variables appear to be more robust correlates of brain size when compared to measures of sociality (see [117]).

Consistent with the literature, support was found for correlations between life-history variables and brain size. As suggested within the developmental cost [110] and maternal energy [119] hypotheses, relationships found possibly reflect the developmental costs associated with growing large brains, which appear to be bypassed through extended developmental periods and increased maternal investment [120, 121]. Similarly, Powell et al. [105] found correlations between neocortex volume and gestation length, as well as cerebellum volume and juvenile period. Whilst the associations found here differ in terms of the specific regions involved, this supports the theory as to why relatively large-brained mammals often exhibit slow maturation times and reduced fertility; thus, by increasing

developmental periods and maternal investment, primates possess these slow life histories which ultimately facilitates the production of big brains. This therefore makes the 'extended parenting' association critical to the evolution of cognition [90, 120, 122, 123]. However, one mystery still left to solve is the reasoning behind the association found here between brain size and maximum longevity. One proposition is that selection mechanisms work towards counterbalancing the costs of large brains in mammals with a longer reproductive lifespan [124], and thus, by extending the reproductive lifespan of a species, it counteracts the time and effort spent producing and maintaining large brains and aims to maximise the time species can spend producing young, which in turn have large brains. Whereas others propose the correlation is indirect and that a longer reproductive lifespan is a by-product of shifting developmental and maturation periods [105].

Carnivores

Akin to the primate results, for carnivores, support is found for a link between regional brain volumes and home range size. This relationship reached significance in the cerebellum models, concurring with research suggesting this region is important for spatial memory processing [1, 125, 126]. Simply, larger home range sizes are thought to require the use of complex information about food location and distribution [9], which for example in carnivores, may represent the challenges of locating travelling herds of herbivores. Alongside this association, indicating spatial demands influence brain size in carnivores, dietary breadth was another ecological variable included in the '*best fit*' endocranial volume models. However, in contrast to the results of MacLean et al. [50] and Swanson et al. [19], the relationship between dietary breadth and brain size is negatively directed, suggesting greater dietary breadth is actually associated with smaller brain size in carnivores. This

result could perhaps be a consequence of those species who are classified as obligate meat eaters, whose dietary breath is limited to one or two categories, thereby producing this negative correlation. Despite this, obligate meat-eating carnivores consume the highest caloric diet, which is thought to provide greater energy for producing large brains. This highlights how carnivores cannot simply be compared and likened to other mammalian orders, such as Primates, and suggests different evolutionary mechanisms at work in carnivoran lineages. It is important to note, however, that this association, whilst close to, failed to reach significance ($P = 0.05$), suggesting this relationship is not a strong influence on brain size in carnivores.

Whilst previous work has suggested sociality plays a role in the evolution of brain size in carnivoran lineages [31, 33-35], here, we find no support for a link between measures of sociality and brain size in carnivores. Similarly, MacLean et al. [50], Benson-Amram et al. [127], and Swanson et al. [19], found no support for the social brain hypothesis in mammals. The contrasting results present in the literature could be due to the fact that sociality appears to be limited to a select few carnivore taxa, specifically social species from the families Hyaenidae, Procyonidae and Felidae [128]. This is suggested in the findings of Finarelli & Flynn [55], who identified that support for the SBH in Carnivora was dependent on data from Canidae, without which, no association is found. Thus, whilst sociality evidently plays an important role in primates, leading to complex, multi-faceted societies, this is less common in carnivore species, and therefore does not hold the same importance.

Consistent with the previously discussed primate results, associations were found between life-history variables and brain size in carnivores. Age at first reproduction, gestation length and maximum lifespan were all found to positively correlate with regional brain volumes,

suggesting both an increase in developmental periods as well as an extension in reproductive lifespans. Additionally, findings are consistent with the expensive brain hypothesis [121], which proposes either an increase in energy turnover or a reduction in energy allocation is needed in order to meet the costs of increased brain size. This is seen here with a negative correlation between fertility and endocranial volume, suggesting a reduction in reproductive output. This, when paired with an increase in maternal investment and developmental periods, as suggested by the aforementioned results, bypasses the developmental constraints of producing a large brain through reduced fertility and slow maturation times.

Whole versus regional brain volumes

Our study highlights the benefit of investigating both whole brain and regional brain volumes. Whole brain volumes are often more readily available for species and thus by choosing to use this brain measure it increases sample sizes and commensurate statistical power. In addition, it has been argued the neocortex comprises a large proportion of whole brain volume, making the two brain volumes closely related [34, 95]. However, it is possible the inclusion of specific brain regions may uncover further associations that were not significant or present before. This was the case here, where for primates, the home range association only became significant in the neocortex and cerebellum models, having not reached significance in endocranial volume models. Additionally, in carnivores, many of the life-history associations, for example age at first reproduction, only reached significance in the regional brain volume models. Therefore, without investigating specific brain regions, the influence of these associations would have been missed. In addition to this, the use of whole brain size does not necessarily allow the study of the ways in which different selective

pressures act on different neural systems, as proposed by theories of mosaic evolution [5, 61]. This often makes it difficult to relate whole brain size to individual selection pressures [129]. By investigating specific brain regions, where brain data and the corresponding covariates are available, it allows the further analysis of how multiple functional systems can evolve in a mosaic fashion in response to different selection pressures.

Conclusion

To conclude, the evidence presented here supports the proposition that ecological variables hold greater influence in determining brain size in primate lineages. However, critical support is also found for the SBH in primates, confirming sociality does hold significance in encephalisation. Ecological variables, most notably home range size, appear to shape carnivoran brain size. Yet no support is found there for measures of sociality, indicating that sociality may not hold the same importance within that order. Life-history traits reveal evidence for the transition to slow life histories, which work toward facilitating the production of big brains and bypassing the cost of expensive brain tissue. Whilst data availability limits the application of comparative studies of brain evolution in many species, future studies should strive to integrate multiple variables, fully encompassing all the potential variables influencing brain size. In addition, where possible, researchers should investigate both whole brain and specific brain regions, as the inclusion of such may reveal further associations, capturing how different brain regions can evolve independently through varying selection pressures.

Acknowledgements

We thank Alex DeCasien for help regarding encephalisation quotients and model comparison analyses. We are grateful to Eli Swanson for valuable discussions regarding PGLS analysis and for providing additional data analysis resources. Our thanks also to F. Sayol, O. Bertrand, V. Weisbecker, N. Emery, M. Tucker, D. Hinchcliffe, M. Olalla-Tárraga, S. Shultz, J. Gundry, C. O'Hara and C. Fauvelle. We are further grateful to the reviewers, whose thorough examinations led to a range of helpful suggestions that greatly assisted us in improving our ms.

Data Accessibility: All data supporting the findings of this study are available in the paper and the supplementary information.

Author Contributions: conceptualisation and methodology, HRC and SOH; investigation and data curation, HRC; formal analysis, HRC, SAH; supervision, SOH; project administration, HRC; visualisation and original draft, HRC, SOH; review and editing, HRC, SOH, SAH.

Funding: This research received no external funding

Conflict of interest: Authors declare no competing interests

References

1. Barton RA. Embodied cognitive evolution and the cerebellum. Philos Trans R Soc B. 2012;367(1599): 2097-2107.

2. Parker ST, Gibson KR. Object manipulation, tool use and sensorimotor intelligence as feeding adaptations in cebus monkeys and great apes. J Hum Evol. 1977;6(7): 623-641.

3. Milton K. Distribution patterns of tropical plant foods as an evolutionary stimulus to primate mental development. Am Anthropol. 1981;83(3): 534-548.

4. Mace GM, Harvey PH, Clutton-Brock TH. Brain size and ecology in small mammals. J Zool. 1981;193(3): 333-354.

5. Barton RA, Purvis A, Harvey PH. Evolutionary radiation of visual and olfactory brain systems in primates, bats and insectivores. Philos Trans R Soc B. 1995;348(1326): 381-392.

6. Hutcheon JM, Kirsch JAW, Garland Jr T. A comparative analysis of brain size in relation to foraging ecology and phylogeny in the chiroptera. Brain Behav Evol. 2002;60(3): 165-180.

7. Winkler H, Leisler B, Bernroider G. Ecological constraints on the evolution of avian brains. J Ornithol. 2004;145(3): 238-244.

8. Harvey PH, Clutton-Brock TH, Mace GM. Brain size and ecology in small mammals and primates. PNAS. 1980;77(7): 4387-4389.

9. Clutton-Brock TH, Harvey PH. Primates, brains and ecology. J Zool. 1980;190(3): 309-323.

10. Bernard RTF, Nurton J. Ecological correlates of relative brain size in some south african rodents. S Afr J Zool. 1993;28(2): 95-98.

11. Barton RA. Primate brain evolution: cognitive demands of foraging or of social life? In: Boinski S, Garber PA, editors. On the move: how and why animals travel in groups. London: The University of Chicago Press; 2000. pp. 204-237.

12. Heldstab SA, Kosonen ZK, Koski SE, Burkart JM, van Schaik CP, Isler K. Manipulation complexity in primates coevolved with brain size and terrestriality. Sci Rep. 2016;6: 24528.

13. Parker ST. Re-evaluating the extractive foraging hypothesis. New Ideas Psychol. 2015;37: 1-12.

14. Powell LE, Isler K, Barton RA. Re-evaluating the link between brain size and behavioural ecology in primates. Proc R Soc B. 2017;284(1865): 20171765.

15. Milton K, May ML. Body weight, diet and home range area in primates. Nature. 1976;259(5543): 459-462.

16. Walker R, Burger O, Wagner J, Von Rueden CR. Evolution of brain size and juvenile periods in primates. J Hum Evol. 2006;51(5): 480-489.

17. Ratcliffe JM. Neuroecology and diet selection in phyllostomid bats. Behav Process. 2009;80(3): 247-251.

18. van Woerden JT, van Schaik CP, Isler K. Effects of seasonality on brain size evolution: evidence from strepsirrhine primates. Am Nat. 2010;176(6): 758-767.

19. Swanson EM, Holekamp KE, Lundrigan BL, Arsznov BM, Sakai ST. Multiple determinants of whole and regional brain volume among terrestrial carnivorans. PLoS One. 2012;7(6): e38447.

20. DeCasien AR, Williams SA, Higham JP. Primate brain size is predicted by diet but not sociality. Nat Ecol Evol. 2017;1(5): 0112.

21. Gibson KR. Cognition, brain size and the extraction of embedded food resources. In: Lee PC, Else JG, editors. Primate ontogeny, cognition and social behaviour. Cambridge: Cambridge University Press; 1986. pp. 93-103.

22. Reader SM, Hager Y, Laland KN. The evolution of primate general and cultural intelligence. Philos Trans R Soc B. 2011;366(1567): 1017-1027.

23. Plante S, Colchero F, Calmé S. Foraging strategy of a neotropical primate: how intrinsic and extrinsic factors influence destination and residence time. J Anim Ecol. 2014;83(1): 116-125.

24. Sol D, Duncan R, Blackburn T, Cassey P, Lefebvre L. Big brains, enhanced cognition, and response of birds to novel environments. PNAS. 2005;102: 5460-5465.

25. Whiten A, Byrne RW. Tactical deception in primates. Behav Brain Sci. 1988;11(2): 233-273.

26. Dunbar RIM. The social brain hypothesis. Evol Anthropol. 1998;6(5): 178-190.

27. Dunbar RIM. The social brain hypothesis and its implications for social evolution. Ann Hum Biol. 2009;36(5): 562-572.

28. Dunbar RIM. Neocortex size as a constraint on group size in primates. J Hum Evol. 1992;22(6): 469-493.

29. Byrne RW, Corp N. Neocortex size predicts deception rate in primates. Proc R Soc B. 2004;271(1549): 1693-1699.

30. Kudo H, Dunbar RIM. Neocortex size and social network size in primates. Anim Behav. 2001;62(4): 711-722.

31. Holekamp KE, Dantzer B, Stricker G, Shaw Yoshida KC, Benson-Amram S. Brains, brawn and sociality: a hyaena's tale. Anim Behav. 2015;103: 237-248.

32. Sakai ST, Arsznov BM, Lundrigan BL, Holekamp KE. Brain size and social complexity: a computed tomography study in hyaenidae. Brain Behav Evol. 2011;77(2): 91-104.

33. Dunbar RIM, Bever J. Neocortex size predicts group size in carnivores and some insectivores. Ethology. 1998;104(8): 695-708.

34. Shultz S, Dunbar RIM. The evolution of the social brain: anthropoid primates contrast with other vertebrates. Proc R Soc B. 2007;274(1624): 2429-2436.

35. Pérez-Barbería FJ, Shultz S, Dunbar RIM. Evidence for coevolution of sociality and relative brain size in three orders of mammals. Evolution. 2007;61(12): 2811-2821.

36. Perez-Barberia FJ, Gordon IJ. Gregariousness increases brain size in ungulates. Oecologia. 2005;145(1): 41-52.

37. Shultz S, Dunbar RIM. Both social and ecological factors predict ungulate brain size. Proc R Soc B. 2006;273(1583): 207-215.

38. Emery N, Seed A, von Bayern A, Clayton N. Cognitive adaptations of social bonding in birds. Philos Trans R Soc B. 2007;362: 489-505.

39. Scheiber IB, Weiß BM, Hirschenhauser K, Wascher CA, Nedelcu IT, Kotrschal K. Does 'relationship intelligence' make big brains in birds? Open Biol. 2008;1: 6-8.

40. Shultz S, Dunbar RIM. Social bonds in birds are associated with brain size and contingent on the correlated evolution of life-history and increased parental investment. Biol J Linn Soc. 2010;100(1): 111-123.

41. Gonzalez-Voyer A, Winberg S, Kolm N. Social fishes and single mothers: brain evolution in african cichlids. Proc R Soc B. 2009;276(1654): 161-167.

42. Bshary R. Machiavellian intelligence in fishes. In: Brown C, Laland KN, Krause J, editors. Fish cognition and behavior. Chichester: Blackwell Publishing Ltd; 2011. pp. 277-297.

43. Triki Z, Levorato E, McNeely W, Marshall J, Bshary R. Population densities predict forebrain size variation in the cleaner fish Labroides dimidiatus. Proc R Soc B. 2019;286(1915): 20192108.

44. Byrne RW, Bates LA. Brain evolution: when is a group not a group? Curr Biol. 2007;17(20): R883-R884.

45. Bergman TJ, Beehner JC. Measuring social complexity. Anim Behav. 2015;103: 203-209.

46. Schillaci MA. Sexual selection and the evolution of brain size in primates. PLoS One. 2006;1(1): e62.

47. Schillaci MA. Primate mating systems and the evolution of neocortex size. J Mammal. 2008;89(1): 58-63.

48. MacLean EL, Barrickman NL, Johnson EM, Wall CE. Sociality, ecology, and relative brain size in lemurs. J Hum Evol. 2009;56(5): 471-478.

49. Silk JB. The evolution of primate societies. In: Mitani JC, Call J, Kappeler PM, Palombit RA, Silk JB, editors. The adaptive value of sociality: University of Chicago Press; 2012. pp. 552-564.

50. MacLean EL, Hare B, Nunn CL, Addessi E, Amici F, Anderson RC, et al. The evolution of self-control. PNAS. 2014;111(20): E2140-2148.

51. van Schaik C, Graber SM, Schuppli C, Heldstab SA, Isler K. Brain size evolution in primates-testing effects of social vs. ecological complexity. Am J Phys Anthropol. 2016;159: 321-321.

52. Holekamp KE, Benson-Amram S. The evolution of intelligence in mammalian carnivores. Interface Focus. 2017;7(3): 20160108.

53. Holekamp KE. Questioning the social intelligence hypothesis. Trends Cogn Sci. 2007;11(2): 65-69.

54. van Schaik CP, Isler K, Burkart JM. Explaining brain size variation: from social to cultural brain. Trends Cogn Sci. 2012;16(5): 277-284.

55. Finarelli JA, Flynn JJ. Brain-size evolution and sociality in carnivora. PNAS. 2009;106(23): 9345-9349.

56. Jerison HJ. Evolution of the brain and intelligence. New York: Academic Press; 1973.

57. Deaner RO, Isler K, Burkart J, van Schaik C. Overall brain size, and not encephalization quotient, best predicts cognitive ability across non-human primates. Brain Behav Evol. 2007;70(2): 115-124.

58. Shultz S, Dunbar R. Encephalization is not a universal macroevolutionary phenomenon in mammals but is associated with sociality. PNAS. 2010;107(50): 21582-21586.

59. Freckleton RP. On the misuse of residuals in ecology: regression of residuals vs. multiple regression. J Anim Ecol. 2002;71(3): 542-545.

60. Freckleton RP. The seven deadly sins of comparative analysis. J Evol Biol. 2009;22(7): 1367-1375.

61. Barton RA, Harvey PH. Mosaic evolution of brain structure in mammals. Nature. 2000;405(6790): 1055-1058.

62. Deaner RO, Nunn CL. How quickly do brains catch up with bodies? A comparative method for detecting evolutionary lag. Proc R Soc B. 1999;266(1420): 687-694.

63. Smaers JB, Dechmann DKN, Goswami A, Soligo C, Safi K. Comparative analyses of evolutionary rates reveal different pathways to encephalization in bats, carnivorans, and primates. PNAS. 2012;109(44): 18006-18011.

64. Smaers JB, Rothman RS, Hudson DR, Balanoff AM, Beatty B, Dechmann DKN, et al. The evolution of mammalian brain size. Sci Adv. 2021;7(18): eabe2101.

65. van Schaik CP, Triki Z, Bshary R, Heldstab SA. A farewell to EQ: A new brain size measure for comparative primate cognition. bioRxiv. 2021: 2021.2002.2015.431238.

66. Finlay B, Darlington RB. Linked regularities in the development and evolution of mammalian brains. Science. 1995;268: 1578-1584.

67. Finlay BL, Darlington RB, Nicastro N. Developmental structure in brain evolution. Behav Brain Sci. 2001;24(2): 263-278.

68. Cantania K. Correlates and possible mechanisms of neocortical enlargement and diversification in mammals. Int J Comp Psychol. 2004;17(1).

69. Innocenti GM, Kaas JH. The cortex. Trends Neurosci. 1995;18(9): 371-372.

70. Kaas JH. The evolution of isocortex. Brain Behav Evol. 1995;46(4-5): 187-196.

71. Barton RA. Visual specialization and brain evolution in primates. Proc R Soc B. 1998;265(1409): 1933-1937.

72. Jacobs GH. The distribution and nature of colour vision among the mammals. Biol Rev. 1993;68(3): 413-471.

73. Jacobs GH. Variations in primate color vision: mechanisms and utility. Evol Anthropol. 1994;3(6): 196-205.

74. Jacobs GH. Primate photopigments and primate color vision. PNAS. 1996;93(2): 577-581.

75. Sussman RW. Primate origins and the evolution of angiosperms. Am J Primatol. 1991;23(4): 209-223.

76. Brothers L. The neural basis of primate social communication. Motiv Emot. 1990;14(2): 81-91.

77. Barton RA. Neocortex size and behavioural ecology in primates. Proc R Soc B. 1996;263(1367): 173-177.

78. Barton RA. Binocularity and brain evolution in primates. PNAS. 2004;101(27): 10113-10115.

79. Barton RA. Olfactory evolution and behavioral ecology in primates. Am J Primatol. 2006;68(6): 545-558.

80. Whiting BA, Barton RA. The evolution of the cortico-cerebellar complex in primates: anatomical connections predict patterns of correlated evolution. J Hum Evol. 2003;44(1): 3-10.

81. Butler AB, Hodos W. Comparative vertebrate neuroanatomy: evolution and adaptation. 2nd ed. New Jersey: John Wiley & Sons Inc; 2005.

82. Iwaniuk AN, Lefebvre L, Wylie DR. The comparative approach and brain-behaviour relationships: a tool for understanding tool use. Can J Exp Psychol. 2009;63(2): 150-159.

83. Obayashi S, Suhara T, Kawabe K, Okauchi T, Maeda J, Akine Y, et al. Functional brain mapping of monkey tool use. NeuroImage. 2001;14(4): 853-861.

84. Barton RA, Venditti C. Rapid evolution of the cerebellum in humans and other great apes. Curr Biol. 2014;24(20): 2440-2444.

85. Wolpert DM, Doya K, Kawato M. A unifying computational framework for motor control and social interaction. Philos Trans R Soc B. 2003;358(1431): 593-602.

86. Oztop E, Wolpert D, Kawato M. Mental state inference using visual control parameters. Cogn Brain Res. 2005;22(2): 129-151.

87. Smaers JB, Vanier DR. Brain size expansion in primates and humans is explained by a selective modular expansion of the cortico-cerebellar system. Cortex. 2019;118: 292-305.

88. Smaers JB, Turner AH, Gómez-Robles A, Sherwood CC. A cerebellar substrate for cognition evolved multiple times independently in mammals. eLife. 2018;7: e35696.

89. Fernandes HBF, Peñaherrera-Aguirre M, Woodley of Menie MA, Figueredo AJ. Macroevolutionary patterns and selection modes for general intelligence (G) and for commonly used neuroanatomical volume measures in primates. Intelligence. 2020;80: 101456.

90. Isler K, van Schaik CP. Allomaternal care, life history and brain size evolution in mammals. J Hum Evol. 2012;63(1): 52-63.

91. DeCasien AR, Higham JP. Primate mosaic brain evolution reflects selection on sensory and cognitive specialization. Nat Ecol Evol. 2019;3(10): 1483-1493.

92. Sakai ST, Arsznov BM, Hristova AE, Yoon EJ, Lundrigan BL. Big cat coalitions: a comparative analysis of regional brain volumes in felidae. Front Neuroanat. 2016;10: 99.

93. Heldstab S, Isler K. Environmental seasonality and mammalian brain size evolution: Wiley Online Library; 2019.

94. Dunbar RIM, Shultz S. Understanding primate brain evolution. Philos Trans R Soc B. 2007;362(1480): 649-658.

95. Dunbar RIM, Shultz S. Why are there so many explanations for primate brain evolution? Philos Trans R Soc B. 2017;372(1727): 20160244.

96. Isler K, Christopher Kirk E, Miller JM, Albrecht GA, Gelvin BR, Martin RD. Endocranial volumes of primate species: scaling analyses using a comprehensive and reliable data set. J Hum Evol. 2008;55(6): 967-978.

97. Stephan H, Frahm H, Baron G. New and revised data on volumes of brain structures in insectivores and primates. Folia Primatol. 1981;35(1): 1-29.

98. Sallet J, Mars RB, Noonan MP, Andersson JL, O'Reilly JX, Jbabdi S, et al. Social network size affects neural circuits in macaques. Science. 2011;334(6056): 697-700.

99. Powell J, Lewis PA, Roberts N, García-Fiñana M, Dunbar RIM. Orbital prefrontal cortex volume predicts social network size: an imaging study of individual differences in humans. Proc R Soc B. 2012;279(1736): 2157-2162.

100. Dunbar RIM, Shultz S. Evolution in the social brain. Science. 2007;317(5843): 1344-1347.

101. Stankowich T, Haverkamp PJ, Caro T. Ecological drivers of antipredator defenses in carnivores. Evolution. 2014;68(5): 1415-1425.

102. Wilman H, Belmaker J, Simpson J, de la Rosa C, Rivadeneira MM, Jetz W. EltonTraits 1.0: Species-level foraging attributes of the world's birds and mammals. Ecology. 2014;95(7): 2027-2027.

103. IUCN. The IUCN red list of threatened species [Internet]. United Kingdom: International Union for Conservation of Nature and Natural Resources; 2021 [updated 2021, cited Jul 2, 2020]. Available from: https://www.iucnredlist.org/.

104. Deaner RO, Barton RA, van Schaik C. Primate brains and life histories: renewing the connection. In: Kappeler PM, Pereira ME, editors. Primate life histories and socioecology. Chicago: The University of Chicago Press; 2003. pp. 233-265.

105. Powell LE, Barton RA, Street SE. Maternal investment, life histories and the evolution of brain structure in primates. Proc R Soc B. 2019;286(1911): 20191608.

106. Isler K, Van Schaik CP. How humans evolved large brains: comparative evidence. Evol Anthropol. 2014;23(2): 65-75.

107. Weisbecker V, Goswami A. Brain size, life history, and metabolism at the marsupial/placental dichotomy. PNAS. 2010;107(37): 16216-16221.

108. DeCasien AR, Thompson NA, Williams SA, Shattuck MR. Encephalization and longevity evolved in a correlated fashion in euarchontoglires but not in other mammals. Evolution. 2018;72(12): 2617-2631.

109. Street SE, Navarrete AF, Reader SM, Laland KN. Coevolution of cultural intelligence, extended life history, sociality, and brain size in primates. PNAS. 2017;114(30): 7908-7914.

110. Barton RA, Capellini I. Maternal investment, life histories, and the costs of brain growth in mammals. PNAS. 2011;108(15): 6169-6174.

111. Barrickman NL, Bastian ML, Isler K, van Schaik CP. Life history costs and benefits of encephalization: a comparative test using data from long-term studies of primates in the wild. J Hum Evol. 2008;54(5): 568-590.

112. Symonds MRE, Blomberg SP. A primer on phylogenetic generalised least squares. In: Garamszegi LZ, editor. Modern phylogenetic comparative methods and their application in evolutionary biology: concepts and practice. Berlin, Heidelberg: Springer Berlin Heidelberg; 2014. pp. 105-130.

113. Upham NS, Esselstyn JA, Jetz W. Inferring the mammal tree: species-level sets of phylogenies for questions in ecology, evolution, and conservation. PLoS Biol. 2019;17(12): e3000494.

114. Schwarz G. Estimating the dimension of a model. Ann Stat. 1978;6(2): 461-464, 464.

115. Aiello LC, Wheeler P. The expensive-tissue hypothesis: the brain and the digestive system in human and primate evolution. Curr Anthropol. 1995;36(2): 199-221.

116. Fish JL, Lockwood CA. Dietary constraints on encephalization in primates. Am J Phys Anthropol. 2003;120(2): 171-181.

117. Graber SM. Social and ecological aspects of brain size evolution: a comparative approach [PhD Thesis]. Zurich: University of Zurich; 2017.

118. Layton R, O'Hara S. Human social evolution: a comparison of hunter gather and chimpanzee social organization. In: Dunbar R, Gamble C, Gowlett J, editors. Social brain, distributed mind. Oxford: British Academy; 2010. pp. 85-115.

119. Martin RD. Scaling of the mammalian brain: the maternal energy hypothesis. Physiology. 1996;11(4): 149-156.

120. Heldstab SA, Isler K, Burkart JM, van Schaik CP. Allomaternal care, brains and fertility in mammals: who cares matters. Behav Ecol Sociobio. 2019;73(6): 71.

121. Isler K, van Schaik CP. The expensive brain: a framework for explaining evolutionary changes in brain size. J Hum Evol. 2009;57(4): 392-400.

122. Heldstab SA, Isler K, Schuppli C, van Schaik CP. When ontogeny recapitulates phylogeny: Fixed neurodevelopmental sequence of manipulative skills among primates. Sci Adv. 2020;6(30): eabb4685.

123. Uomini N, Fairlie J, Gray RD, Griesser M. Extended parenting and the evolution of cognition. Philos Trans R Soc B. 2020;375(1803): 20190495.

124. González-Lagos C, Sol D, Reader S. Large-brained mammals live longer. J Evol Biol. 2010;23: 1064-1074.

125. Leggio MG, Chiricozzi FR, Clausi S, Tedesco AM, Molinari M. The neuropsychological profile of cerebellar damage: the sequencing hypothesis. Cortex. 2011;47(1): 137-144.

126. Rochefort C, Arabo A, André M, Poucet B, Save E, Rondi-Reig L. Cerebellum shapes hippocampal spatial code. Science. 2011;334(6054): 385-389.

127. Benson-Amram S, Dantzer B, Stricker G, Swanson EM, Holekamp KE. Brain size predicts problem-solving ability in mammalian carnivores. PNAS. 2016;113(9): 2532-2537.

128. Sakai ST, Arsznov BM. Carnivoran brains: effects of sociality on inter- and intraspecific comparisons of regional brain volumes. In: Kaas JH, editor. Evolutionary neuroscience. 2nd ed. London: Academic Press; 2020. pp. 463-479.

129. Healy SD, Rowe C. A critique of comparative studies of brain size. Proc R Soc B. 2007;274(1609): 453-464.

Supporting information

S1 File. Supplementary analyses. This document includes information about the extra analyses conducted using different measures of brain size.

S2 File. Supplementary results tables. This document includes all the supplementary results tables associated with the supplementary analyses.

S3 File. Supplementary BIC scores. This excel file includes all the BIC scores used to conduct model comparisons during the main analyses.

S4 File. Additional BIC scores. This excel file includes all the BIC scores used to conduct model comparisons during the extra analyses.

S5 File. Supporting data. This excel file includes all the data used within the statistical analyses.

S6 File. VIF results. This document includes all the VIF score results.

S7 File. Data collection sources. This document includes all the data collection sources.

S8 File. R code. This text file contains the R script used to conduct the statistical analyses.

S9 File. Phylogenetic tree. This file is the phylogenetic tree used during statistical analyses.

S1 File

Click here to access/download
Supporting Information
S1 File_Supplementary analyses.docx

S2 File

Click here to access/download
Supporting Information
S2 File_Supplementary results tables.docx

S3 File

Click here to access/download
Supporting Information
S3 File_Supplementary BIC scores.xlsx

S4 File

Click here to access/download
Supporting Information
S4 File_Additional BIC scores.xlsx

S5 File

Click here to access/download
Supporting Information
S5 File_Supporting data.xlsx

S6 File

Click here to access/download
Supporting Information
S6 File_VIF results.docx

S7 File

Click here to access/download
Supporting Information
S7 File_Data collection sources.docx

S8 File

Click here to access/download
Supporting Information
S8 File_R code.txt

S9 File

Click here to access/download
Supporting Information
S9 File_Phylogenetic tree

Title: Why big brains? A comparison of models for both primate and carnivore brain size evolution

Running title: Why big brains?

Authors: Helen R Chambers[1],[3], Sandra A Heldstab[2], Sean J O'Hara[1]

[1]University of Salford, School of Science, Engineering & Environment, Peel Building, Salford, Greater Manchester, M5 4WT, United Kingdom

[2]Department of Anthropology, University of Zurich, Winterthurerstrasse 190, CH-8057 Zurich, Switzerland

[3]Correspondence: h.r.chambers@edu.salford.ac.uk

Orchid IDs:

https://orcid.org/0000-0001-7251-1166

https://orcid.org/0000-0002-8908-7522

https://orcid.org/0000-0002-5909-1870

Abstract

Despite decades of research, much uncertainty remains regarding the selection pressures responsible for brain size variation. Whilst the influential social brain hypothesis once garnered extensive support, more recent studies have failed to find support for a link between brain size and sociality~~ acting as an evolutionary driver~~. Instead, it appears there is now substantial evidence suggesting ecology better predicts brain size in both primates and carnivores. Here, different models of brain evolution were tested, and the relative importance of social, ecological, and life-history traits were assessed on both overall encephalisation and specific brain regions. In primates, evidence is found for consistent associations between brain size and ecological factors, particularly diet; however, evidence was also found advocating sociality as a selection pressure driving brain size. In carnivores, evidence suggests ecological variables, most notably home range size, are influencing brain size; whereas, no support is found for the social brain hypothesis, perhaps reflecting the fact sociality appears to be limited to a select few taxa. ~~In carnivores, evidence suggests multiple selection pressures, both ecological and social, are influencing brain size within the Carnivora order.~~ Life-history associations reveal complex selection mechanisms to be~~large-brained primates and carnivores are~~ counterbalancing the costs associated with expensive brain tissue through extended developmental periods, reduced fertility, and extended maximum lifespan. ~~Critically, the use of different body size correction factors, such as relative brain size or encephalisation quotient, yielded disparate results. This hinders modern research, as without clarity regarding what is the most suitable correction measure, there is little certainty concerning the 'true' correlates of brain size.~~ Future studies should give careful consideration of the ~~body size correction factors~~methods chosen for measuring

brain size~~ as outlined herein~~, investigate both whole brain and specific brain regions where possible, and look to integrate multiple variables, thus fully capturing all of the potential factors influencing brain size.

Key words: brain size, primates, carnivores, sociality, diet, encephalisation

Introduction

Brain size varies considerably amongst mammals; substantial variation is seen among~~st~~ primates, where brain size varies almost a thousand-fold across the order (1)~~(Barton, 2012)~~. The adaptive value of such variation has come under extensive scrutiny over the past few decades and yet despite considerable research effort, much uncertainty remains regarding the selection pressures responsible.

Frequently proposed to explain variation in brain size are factors related to the physical environment, such as diet and home range size, as well as factors related to the social environment, such as group size and pair-bondedness. Ecological hypotheses mainly involve investigating the cognitive demands associated with foraging (2-7)~~(Parker & Gibson, 1977; Milton, 1981; Barton, Purvis & Harvey, 1995)~~, as foraging is considered mentally demanding due to the pressure of managing, processing and remembering spatial and temporal information about resource availability (8-12)~~(Clutton-Brock & Harvey, 1980; Barton, 2000; Heldstab et al., 2016)~~. Additionally, differing home range size is ~~have been~~ of interest to researchers ~~for many years;~~ due to the supposed cognitive demands imposed by larger home ranges, such as processing requirements of navigating spatially-complex information,

especially in terms of food availability, location and distribution (9, 13-15). This has resulted in many studies investigating the cumulative effects of the physical environment on encephalisation, with a specific interest in diet (16-20)~~(Walker et al., 2006; (17)van Woerden et al., 2010;(18) Swanson et al., 2012; DeCasien et al., 2017)~~, home range (13, 14)~~(Parker, 2015; Powell et al., 2017)~~, foraging techniques (12, 21-23)~~(Gibson, 1986;[Reader, 2011 #220] Reader et al., 2011; Plante et al., 2013; Heldstab et al., 2016)~~ and behavioural responses in a fluctuating environment (24)~~(Sol et al., 2005)~~.

In contrast to ecological hypotheses, the social brain hypothesis (SBH) suggests sociality – specifically the cognitive demands of tracking, negotiating and maintaining social relationships – to be the main driving force behind variation in primate brain sizes (25-27)~~(Whiten & Bryne, 1988; Dunbar, 1998; Dunbar, 2009)~~. The study of primates lends credence to this hypothesis, with brain size found to correlate with many social proxies, such as social group size (28)~~(Dunbar, 1992)~~, tactical deception (29)~~(Bryne & Corp, 2004)~~ and grooming clique size (30)~~(Kudo & Dunbar, 2001)~~. Evidence has since not been limited to studies of the primate lineage, with corroboration coming from research on spotted hyenas (31, 32)~~(Holekamp et al., 2015; Sakai et al., 2011),~~ as well as other carnivorans (33-35)~~(Dunbar & Bever, 1998; Shultz & Dunbar, 2007; Pérez-Barbería et al., 2007;)~~, ungulates (36, 37),~~(Pérez-Barbería & Gordon, 2005; Shultz & Dunbar, 2006; Emery et al., 2007)~~ birds (38-40),~~(Scheiber et al., 2008; Shultz & Dunbar, 2010)~~ and some fish species (41-43)~~(Gonzalez-Voyer et al., 2008; Bshary, 2011; Triki et al., 2019)~~. The focal point of much of the early work investigating sociality was social group size, due to the information-processing demands group of increasing sizes are thought to incur (26). However, the use of this proxy for measuring social complexity has been criticised (44)~~,~~ and instead, focus has shifted to the consequences of varying levels of relationship complexity (45). ~~Additionally,~~

~~since being established, the SBH has further developed, moving away from the original focal point of group size,~~ and toward investigating the influence of pair-bondedness (27, 46-48)~~(Dunbar, 2009)~~. This developed from the proposition that relationship quality (45, 49)~~(Silk, 2012; Bergman & Beehner, 2015)~~ connotes cognitive complexity.

Despite the hypothesis receiving considerable support in the past, more recent investigations have failed to find statistical support for a link between brain size and sociality~~ (e.g.,~~ (14, 19, 20, 50, 51)~~Swanson et al., 2012; MacLean et al., 2014; van Schaik et al., 2016; Powell et al., 2017; DeCasien et al., 2017)~~. Instead, it appears there is now substantial, strong, phylogenetically-corrected comparative data reinforcing the assertion that diet better predicts brain size in both primates and carnivores (14, 20, 52)~~(Holekamp & Benson-Amram, 2017)~~. In addition, the obvious exceptions to the SBH, taxa who possess large brains but who are not considered social, suggest factors other than sociality may be influencing brain size (19, 53, 54)~~(Holekamp, 2007; Swanson et al., 2012; van Schaik et al., 2012)~~. For example, if sociality is to be accepted as the causal agent for increased encephalisation in mammals, it should be wide-spread across bears and musteloids, who show similar encephalisation increases to Canidae (55)~~(Finarelli & Flynn, 2009)~~.

A further problem to have dogged comparative analyses of brain evolution is deciding on the correct brain measure. Whilst most studies tend to focus on whole brain size, even this can become an arduous task since there is little clarity in the literature regarding the most appropriate body size correction factor, making decisions on the correct method of choice challenging. Typically, cognitive abilities are estimated using relative brain size, by taking residuals from a regression curve or calculating encephalisation quotients (56, 57)~~(van Schaik, Isler & Burkart, 2012)~~. This became the method of choice when brain and body size

were found to be tightly coupled allometrically across vertebrates; therefore, accounting for this allometric relationship became of great importance (35, 58)~~(Shultz & Dunbar, 2010)~~. ~~Thus, relative brain size is now favoured, as absolute brain size has been found to be “invariably strongly correlated with body size” in most taxa (Pérez-Barbería et al., 2007, p. 2812).~~ However, the use of relative brain size and encephalisation quotients is not without criticism;~~,~~ for example, using residuals as data points in regression models has been ~~heavily~~ discouraged, as the estimates produced are thought to be biased, which influences ~~any~~ subsequent analyses (59, 60). E~~with e~~ncephalisation quotients possibly reflect~~ing~~ the result of recent decreases or increases in body size (61)~~(Barton, 2000)~~, evidence ~~of which~~for such was uncovered by (19). They~~, who~~ found carnivore brain size to lag behind body size over evolutionary time, therefore hinting that the use of brain estimates may be a poor representation of carnivore brain size. H~~; h~~owever, no evidence for ~~this brain size~~a lag ~~hypothesis was~~is found for primates (62)~~(Deaner & Nunn, 1999)~~, suggesting ~~this may not be the case~~a taxonomic difference for this group~~ (Deaner & Nunn, 1999)~~. Alongside this, ~~-~~t~~and~~ ~~t~~he prevalent use of relative brain size is thought to possibly hide~~ing~~ other evolutionary pathways which may be influencing adaptations in body mass (63)~~(Smaers et al, 2012)~~. F~~, f~~or example, a recent analysis of mammalian brain size found the brain-to-body relationship to uncover more than just selection on brain size, indicating relative brain size measures are not accurately capturing brain size variation (64). Thus, (65) suggest~~s~~ the use of encephalisation quotients should be avoided in future studies, as EQs ~~have~~repeatedly fail~~ed~~ to accurately predict brain size, and thus, varying levels of cognitive ability. For example, (57)~~. Swanson et al. (2012) for example, found carnivore brain size to lag behind body size over evolutionary time, therefore hinting that the use of brain estimates may be a poor representation of carnivore brain size. Deaner et al. (2007)~~ found absolute brain size

measures, over statistically controlled produced methods i.e., residuals, to be the best predictors of primate cognitive abilities. In fact, van Schaik et al., (2021) suggest the use of encephalisation quotients should be avoided in future studies.

Alongside the use of total brain size, particular emphasis has been put on specific brain regions in recent years. The social brain hypothesis suggests the neocortex is the brain structure of interest, with primates' large brains thought to be mainly the consequence of a dramatic increase in neocortical volume (66-68)(Finlay & Darlington, 1995; Finlay et al., 2001; Cantania, 2004). The neocortex is thought foremost responsible for the processing of more demanding cognitive and social skills (69, 70)(Innocenti & Kaas, 1995; Kaas, 1995) associated with intelligent and flexible behaviour (61)(Barton & Harvey, 2000). Neocortical enlargement in primates is thought to be partly due to selection on visual mechanisms (71) which is important for frugivorous species, for example who havewhen needing to distinguish between fruits of different colours (72-74) or have towhen manipulatinge small fruit and seeds thatwhich require fine motor coordination (75). Alternatively, these visual mechanisms are thought to be important for processing complex and rapid social interactions, including understanding facial expressions, gaze direction and posture (76), suggesting that neocortical modifications associated with complex social lives primarily involve areas specialiszed for visual processing of social information (77). In primates, the neocortex constitutes a substantial portion of the brain (66, 67), and a large proportion of the neocortex is comprised of visual information processing areas (71, 78, 79), which is thought to explain links found between frugivory and brain size (see -(20)), as well as, social group size and neocortex volume (see (1, 71)).

Alongside research into the neocortex, attention has beenis focused on the cerebellum and its importance. The cerebellum was found to co-evolve with the neocortex (61)(Barton & Harvey, 2000), with a significant correlation found between these two brain regions (80)(Barton, 2002). Increased cerebellar volume is suggested to allow increased processing capacity, in terms of enhanced motor abilities and manipulative abilities (81, 82). F, for example, in primates positive correlations awere found between cerebellum volume and extractive foraging techniques (1), as well as the presence of neural activation in the cerebellum during tool use in monkeys (83)., suggesting the cerebellum is important This highlights the influential role played by the cerebellum in technical intelligence (84). Alongside this, the cerebellum is thought to be important in social intelligence (1), particularly in terms of the links between sensory-motor control and social interactions and understanding (85, 86). Indeed, it is now thought the expansion of the cortico-cerebellar system is the primary driver of brain expansion in anthropoid primates (87)(Smaers & Vanier, 2019), suggesting the increased behavioural complexity in mammals could be partly explained by selection on the cerebellum (88)(Smaers et al., 2018). So much so, that (89)Fernandes et al. (2020) found residual cerebellar size to be the most appropriate proxy when compared to a measure of general intelligence models; as cerebellar models produced the most similar model fit results when compared to those produced using a measure of general intelligence.

Here, using data collected aggregated from the literature the, the relative importance of social, ecological and life history traits awere assessed on both overall encephalisation and specific brain regions, and different models of brain size evolution awere tested. Considerable attention has been paid to primate brain evolution, perhaps since there are substantial data available on this taxonomic group (e.g., (14, 20, 90, 91)Isler & van Schaik,

2012; Powell et al., 2017; DeCasien et al., 2017; DeCasien & Higham, 2019) perhaps since there are substantial data available on this taxonomic group making comparative tests easy to implement. Likewise, cCarnivorans have are also begun now receiving attention (e.g., (19, 88, 92, 93)Swanson et al., 2012; Sakai et al., 2016; Smaers et al., 2018; Heldstab et al., 2019) with their since variation in their brain and body size, and ranging social and physical environments, makesing them excellent models for these tests too. Indeed, most of the literature surrounding brain size hypotheses is based on analyses of these two groups.

One aim here, therefore, is to provide Thus, by drawing greater clarity within these two groups, this will hopefully allow for more reliable and robust analyses of other taxonomic groups. Integrating predictors into a framework which allow the assessment of multiple hypotheses simultaneously has become increasingly important for tests of brain evolution (94, 95)(Dunbar & Shultz, 2007, 2017). Therefore, phylogenetically-corrected generalised least squares (PGLS) models awere used here to account for shared evolutionary history, whilst assessing the potential variables influencing encephalisation.

We use a recently updated phylogenetic tree to ensure phylogenetic relationships are contemporary. Further, the inclusion of multiple variables allows the comparison of multiple hypotheses, as well as models of varying complexity. While brain data are available for more taxa than are included in our dataset, we found some limitations on the completeness of the necessary covariate data. We present here our analyses of two orders where complete datasets with all covariates are available for all species, ensuring the most robust model comparisons. Ultimately, this study aimed to go beyond previous studies of brain size evolution, which are often restricted by small sample sizes and the method of addressing only one hypothesis at a time, to draw more robust and reliable results on the proposed

Formatted: Not Highlight

~~correlates of brain size within two well-studied taxonomic groups. To achieve this, we implemented the use of robust statistical analyses which accounted for the influence of relatedness, a recently updated phylogenetic tree to ensure phylogenetic relationships were contemporary, substantial sample sizes which surpassed previous studies and increased statistical power, and importantly, the inclusion of multiple variables which allowed the comparison of multiple hypotheses, as well as, models of varying complexity.~~ ~~Additionally, to further investigate the use and merits of different brain measures in comparative analyses, multiple measures of brain size were used, including the calculation of encephalisation quotients (EQ) and use of residuals as relative brain size (RBS).~~

Methods

Data Collection

Brain data

Endocranial volume (ECV) and body mass data for primates (n = 83) and carnivores (n = 85) were compiled from multiple sources (see supplementary material). Volumes were matched for species composition and predictor variables, and whilst this resulted in smaller sample sizes when compared to available brain data, in doing so it provided a complete dataset with all covariates available for all species, better enabling robust analyses. ECV data were preferred over brain mass data since it is thought ECV provides a more reliable estimate of brain size, due to the influence of preservation techniques on brain mass (96)~~(Isler et al., 2008)~~. ~~ECV is most frequently estimated~~The standard technique for estimation of E~~V~~CV is through~~by~~ filling the cranium with beads (or similar), which is then measured using a

graduated cylinder or by weighing the beads and converting the weight to volume (96). Neocortex and cerebellum volumes were also collated, where available, for both primates (Neo = 52, Cere = 49) and carnivores (Neo = 44, Cere = 38). These two brain measures were selected for use in the analyses since both have received much attention from researchers in recent years. Regional brain volumes are commonly measured using one of two different techniques:, virtual endocasts (e.g., (19)) or physical sectioning of the individual brain volumes using paraffin and staining substances (e.g., (97)). When sourcing allwhole and regional brain volumes these measurement methods were considered to ensure the data wereas comparable; for example, all ECV data sources used common measurement techniques (as described above) making the whole brain data comparable across multiple studies.These two brain measures were selected for use in the analyses since both have received much attention from researchers in recent years. The neocortical region became the focus of many comparative analyses, especially since the SBH was proposed (Dunbar, 2009). Attention, however, has now shifted to the cerebellum, as it is thought to play more of a substantial role in cognition than was previously assumed (Barton, 2012; Barton & Venditti, 2014).

Social data

Both social group size and social cohesion data were collected for primates and carnivores. Group size – based on the simple principle that as group size increases, the information-processing demands (26)(Dunbar, 1998) and corresponding internal structures (98, 99)(Sallet et al., 2011; Powell et al., 2012) should also increase – became perhaps the most commonly used proxy for social complexity. Despite this, the use of this proxy has been criticised as it is often considered crude, weak, and not always relevant (44)(Byrne & Bates,

~~2007)~~. Greater attention is now paid to differing levels of relationship complexity (45)~~(Bergman & Beehner, 2015)~~ often indicated through the presence of pair-bonds (27, 34, 100)~~(Shultz & Dunbar, 2007; Dunbar & Shultz, 2007; Dunbar, 2009)~~. Therefore, to ensure the influence of sociality was fully captured, alongside group size, a social cohesion proxy was used: a categorisation system ranging from 1) being primarily solitary living aside from breeding seasons, 2) pair-living, 3) fission-fusion societies, to 4) being obligatorily social~~(one to four)~~ (e.g., (91, 101)~~DeCasien & Higham, 2019; Stankowich et al., 2014~~). This index aims to better encapsulate sociality, rather than relying solely on group size numbers.

Ecological data

Four ecological variables were chosen for analysis: dietary categories, dietary breadth, habitat variability and home range size. Dietary categories were assigned following previous designations in the published literature (see supplementary material for sources) and included six different categories: carnivorous, herbivorous, piscivorous, folivorous, frugivorous and omnivorous. Alongside this traditional classification system, dietary breadth was also used, estimated using the total number of food sources used by a species, with data taken from ~~Wilman et al.~~ (102)~~(2014)~~. This included a total of 10 different food types: invertebrates, mammals and birds, reptiles, fish, unknown vertebrates, scavenge, fruit, nectar, seed~~-~~ or other plant material, marked either as absent (0) or present (1). For this dataset, this resulted in a dietary breadth scale of one to six. ~~By incorporating both of these dietary variables into analyses, this allows further investigation into the role diet has in brain evolution. For example, certain diets are thought to be more cognitively demanding, such as, frugivory, which is assumed to require greater spatial memory and food processing techniques, potentially leading to increased encephalisation (Milton, 1981; Parker & Gibson,~~

~~1997; Barton, 2000).~~ Habitat variability, another ecological measure, was formed using data from the IUCN Red List (103)~~(2020)~~, based on the total number of habitat-types used by a species, following the same habitat classification system used in the IUCN Red List. Additionally, home range size data were collected. ~~This variable has been of interest to researchers for many years; due to the supposed cognitive demands imposed by larger home ranges, such as processing requirements of navigating spatially-complex information, especially in terms of food availability, location and distribution (Clutton-Brock & Harvey, 1980; Parker, 2015; Powell et al., 2017).~~ By including variables related both to diet and habitat~~(i.e., imposing both temporal and spatial cognitive demands)~~, it allowed~~s~~ greater incorporation of possible variables within the physical environment affecting brain size.

Life-history data

Life-history variables have been found to be critical in counterbalancing the costs of increased brain size and facilitating ~~exert considerable influence on cognitive evolution and~~ the growth of large brains (104)~~(van Schaik and Deaner, 2003)~~. In fact, they appear ~~crucial~~ to be influencing~~in determining~~ the potential adaptive pathways available to a species (94)~~(Dunbar & Shultz, 2007)~~, for example in terms of balancing shifting developmental and maturation periods. Developmental costs are also thought to influence correlations between specific primate brain structures and life history variables, with the neocortex most strongly correlated with gestation length, and the cerebellum with juvenile period length, suggesting that these brain regions exhibit distinct life-history correlates which concur with their unique developmental trajectories (105). Hence, it ~~seemed~~ was necessary to include certain life history variables in the analysis to further understand how life-history characteristics potentially act as a filter (104, 106)~~(Isler & van Schaik, 2014; van Schaik and~~

~~Deaner, 2003)~~ for the production of large brains. Gestation length was chosen as it has received considerable attention and is thought to be of great importance in bypassing the constraints of precociality in mammals and facilitating brain growth (107)~~(Weisbecker & Goswami, 2010)~~. Maximum lifespan was included as there is substantial support that encephalisation is correlated with extended longevity (104)~~(van Schaik and Deaner, 2003)~~, especially in primates (108, 109)~~(DeCasien et al., 2018; Street et al., 2017)~~. The relationship found between brain size and lifespan is thought to be driven primarily by maternal investment, with subsequent correlations found between specific brain regions and developmental periods, reflecting this brain size-lifespan association (see (105, 110). Ultimately encephalisation has been found to correlate with expansion of most developmental life history stages, including an extended reproductive lifespan (111)~~(Barrickman et al., 2008)~~. ~~, t~~Therefore, data on age at first reproduction, weaning and fertility (measured as number of offspring per year) were added to our dataset (see supplementary material for sources).

Statistical Analyses

Brain transformations

Whole b~~B~~rain volumes were incorporated in analyses by~~using three different methods: (1)~~ simple incorporation of log ECV~~brain~~ volume with log body mass included as a covariate. ~~, (2) using residuals from a regression line, and (3) calculation of encephalisation quotients.~~ Thise ~~former~~ method is often preferred over the use of residuals as variables in ecological datasets often covary thereby producing biased parameter estimates when calculating residuals (59)~~(Freckleton, 2002)~~. Including body mass as a covariate in the model avoids this problem, controls for its effect on brain volume, as well as potentially controlling for any

effects body mass may have on other variables included. Despite criticism, the use of residuals is still present, therefore we considered it prudent to use both methods in the analyses for comparative purposes. Phylogenetic generalised least-squares regression analysis (PGLS) was used to regress log brain volume against log body mass, which produced residual estimates of relative brain size after accounting for body mass. This was repeated for neocortex and cerebellum volumes. Encephalisation quotients were also calculated as a further measure of relative brain size. EQs were derived from our dataset, using the allometric formula $E = kP\alpha$, where E = brain mass, P = body mass, k = y-intercept (proportionality constant) and α = allometric exponent. For the primate data this made the final equation: brain volume / (0.073 x body mass$^{0.80}$). For the carnivore data this made the final equation: brain volume / (0.145 x body mass$^{0.65}$). Regional brain volumes were incorporated in analyses by simple incorporation of log ROB (rest of brain) volume. To calculate ROB volume for both the neocortex and cerebellum, a simple calculation was performed: whole brain volume minus the region volume of interest. This method has been previously implemented and proved useful in measuring relative regional brain volumes (e.g., see (91)). Further analyses were also conducted in order to test how uniform results were when using different brain size measures. The results of these analyses are displayed and discussed in the supplementary material.

This allowed for seven different brain calculation inputs, (1) log ECV with log body mass, (2) relative brain size, (3) encephalisation quotients, (4) relative neocortex volume, (5) relative cerebellum volume, (6) log neocortex volume with log body mass, (7) log cerebellum volume with log body mass.

PGLS analysis

All statistical analyses were performed using R 4.0.1, using the 'caper', 'ape' and 'geiger' packages. Phylogenetic generalised least-squares (PGLS) regression analysis ~~(PGLS) analysis~~ was used to identify those variables influencing whole and regional brain evolution, whilst avoiding the problem of phylogenetic non-independence. This technique differs from standard generalised least squares analysis, as it uses knowledge of phylogenetic relationships or relatedness to produce estimates of the expected covariance across species (112). Pagel's λ was estimated by maximum likelihood. The tree used for all phylogenetic analyses was that of Upham et al's (113)~~(2019)~~. All continuous variables, brain volumes and body mass were log transformed prior to analysis to satisfy the assumption of normality. Variance Inflation Factor (VIF) scores were used to check for the presence of multicollinearity, with almost all scores found to be below 5, and no scores above 7. There were no scores produced which highlighted concern, and thus, all socioecological and life-history variables were retained for analysis ~~8~~ (see supplementary material).

Model comparisons

A series of PGLS models were implemented which varied in complexity, including 1) social, 2) ecological, 3) social and ecological, 4) life history and 5) variables of interest. Models one to four included all possible combinations of the selected variables; for example, the social model included i) group size, ii) social cohesion, iii) group size and social cohesion. BIC (Bayesian Information Criterion) values of each model were then compared (114). As lower BIC values indicate the presence of better fitting, more parsimonious models, the model with the lowest BIC value was deemed to best explain the data, therefore considered preferrable and retained. BIC values were preferred over Akaike Information Criterion values because BIC resolves the problem of overfitting, by using a more conservative

penalty for additional variables. AIC (Akaike Information Criterion) values of each model were then compared (Akaike, 1974). As lower AIC values indicate the presence of better fitting, more parsimonious models, the model with the lowest AIC value was deemed to best explain the data, therefore considered preferrable and retained. Thus, Mmodel number five was constructed using all variables previously highlighted of interest within the social, ecological, and life history models. This allowed us to compare the importance of social versus ecological models, as well as construct models including those variables that best explained the data. Once compuleted, model five was compared alongside the previous models, and those found to have the lowest BIC value were then considered the '*best fit*' models, which in some cases represents a subset of models (simply, any model within dBIC<2 of the lowest model). This is because BIC values with a differencet of between 2 and 6 indicate moderate evidence that the model with the lower BIC provides a relatively better model fit, whilst greater than 6 indicates strong evidence for improved fit. presently, and subsequently, brain size.

Formatted: Font: Italic

Possible Limitations

The *a priori* protocol followed here, that resulted in the model with the absolute lowest AIC score being considered preferrable and retained, does draw certain limitations. For example, there may be another model within two AIC units of the 'preferred' model, meaning there is no statistical difference between the two. Therefore, when compared, the 'preferred' model and the correlations found therein, are not considered superior or more reliable. However, this *a priori* protocol was chosen to ensure systematic uniformity in procedure and to allow the construction of models using variables of interest.

Results

Primates

The results from PGLS analysis on the primate data is shown in Table 1. Almost all models were highly significant. For most models λ was close to one, indicative of a Brownian motion model of trait evolution; however, certain neocortex models ~~both cerebellum models (relative cerebellum size and log cerebellum volume)~~ stand in contrast to this, with λ equal to zero, implying the data have no phylogenetic structure (84)~~(Barton & Venditti, 2014)~~. Combined models were preferred~~-~~ when investigating both whole and regional brain volumes~~when using all but one (EQ) brain inputs~~, with significantly improved (equal or greater than two ~~AIC~~ BIC units lower than another) ~~AIC~~ BIC scores when combining variables indicated to be of importance in previous model iterations. When comparing the influence of ecology versus sociality, ~~In contrast to this, within the EQ models, there was no significant difference between the ecological, social & ecological, and combined models. Despite this, when using all brain transformation methods, e~~ecological models were found to be preferable to social models, evidenced by the presence of significantly improved ~~AIC~~ BIC scores.

Table 1 about here

Overall encephalisation

The results of PGLS analysis on endocranial volume~~,~~ data ~~relative brain size and encephalisation quotient~~ are presented in Table 1, with the '*best fit*' models presented in Table 2. The variables which were indicated to be of importance and included ~~with~~in the

Formatted: Font: Italic

'*best fit*' endocranial volume models were: group size, dietary breadth, gestation length, maximum lifespan and weaning age. Also present ~~with~~in the subset of '*best fit*' models were: social cohesion and home range. After accounting for phylogeny, both group size and social cohesion were found to be positively associated with ECV (P <0.05). Although, social cohesion ~~did~~failed to reach~~find~~ significance in certain model iterations (P = 0.06). In terms of the ecological variables, dietary breadth was consistently associated with ECV (P <0.001);~~,~~ however, home range size failed to reach~~find~~ significance (P = 0.11). Three of the life-history variables were significantly associated with ECV: gestation length, maximum lifespan and weaning age (P <0.01). ~~After accounting for phylogeny, diet was found to be positively associated with all whole brain measures. Dietary breadth was positively associated with ECV (P <0.001), RBS (P <0.001) and EQ (P <0.01). In addition, omnivory was positively associated with RBS and EQ (P <0.01). As well as the dietary variables, habitat variability was negatively associated with EQ (P <0.05). Three of the life-history variables were significantly associated with ECV: gestation length, maximum lifespan and weaning age (P <0.01), however only gestation length was found to be associated with RBS (P <0.05). Social cohesion was close to being associated with ECV; however, this association fell short of significance (P = 0.06). Frugivory, habitat variability and weaning age were also close to being associated with RBS (P = 0.07, P = 0.09, P = 0.06 respectively). In addition, frugivory was close to being significantly correlated with EQ (P = 0.07).~~

Formatted: Font: Italic

Formatted: Font: Italic

Table 2 about here

Regional brain volumes

The results of PGLS analysis on the neocortex and cerebellum ~~models~~ data are ~~also~~ presented in Table 1, with the '*best fit*' models presented in Table 2~~2~~. The variables which were indicated to be of importance and included within the '*best fit*' neocortex model were: diet, home range size, maximum lifespan and weaning age. After accounting for phylogeny, diet, specifically frugivory and omnivory were found to be negatively associated with neocortex volume (P <0.05, P <0.001). This is the result produced when a folivorous diet is used as the baseline category, therefore the dietary category results produced here only demonstrates differences between these dietary groups (frugivory and omnivory) and folivory. Alongside these associations, home range size was positively correlated with neocortex volume (P <0.01). Similar to whole brain models, both maximum lifespan and weaning age were significantly associated with neocortex volume (P <0.001).

The variables which were indicated to be of importance and included ~~with~~in the '*best fit*' cerebellum models were: diet, home range size, maximum lifespan and weaning age. Also present within the subset of '*best fit*' models was: gestation length. After accounting for phylogeny, diet, specifically omnivory was found to be negatively associated with cerebellum volume (P <0.01). Frugivory failed to be significant (P = 0.13, P = 0.31). As above, this ~~is the~~ results ~~produced~~ when ~~a~~ folivorous diet is used as the baseline category. Home range size was positively associated with cerebellum volume (P <0.001). Similar to previous life-history results, maximum lifespan and weaning age were significantly associated with cerebellum volume (P <0.01, P <0.001). Gestation length was close to being significantly correlated with cerebellum volume (P = 0.07). ~~After taking phylogeny into account, diet was found to be positively associated with both brain regions. Frugivory and omnivory were positively associated with RNS (P <0.001, P <0.001), neocortex volume (P <0.01, P <0.001), as well as RCS (P <0.01, P <0.001) and cerebellum volume (P <0.001, P <0.001). Social~~

Formatted: Font: Italic

Formatted: Font: Italic

Formatted: Font: Italic

Formatted: Font: Italic

cohesion was found to be significantly correlated with cerebellum volume (P <0.05). Similarly, to whole brain measures, gestation length was associated with neocortex volume (P <0.05) and weaning age was correlated with cerebellum volume (P <0.05). Additionally, fertility was found to be negatively associated with relative cerebellum volume (P <0.05). Gestation length was close to being associated with relative neocortex size; however, this association fell short of significance (P = 0.06). Additionally, social cohesion and dietary breadth were close to being significantly correlated with neocortex volume (P = 0.06).

Carnivores

The results of PGLS analysis on the carnivore datas data are presented Table 3. Almost all models were highly significant. Lambda was not consistent between the models, ranging from one to zero across the dataset. In terms of the '*best fit*' models, those producing the lowest BIC score (or any score within dBIC<2 of the lowest model), there was no significant difference between life history and combined models, and thus the results of all these models are discussed below. When comparing the influence of ecology versus sociality, ecological models were found to be preferable to social models when investigating regional brain volumes, evidenced by the presence of significantly improved BIC scores. However, this was not the case in whole brain models, where there was no significant difference between the preferred social and ecological models. All models including body mass were highly significant; however, only seven models were found to be significant when using different body size transformation methods. Lambda was not consistent between the models, ranging from one to zero across the dataset. Whilst the combined models had the lowest AIC scores for most of the carnivore models, these scores were not significantly improved

Formatted: Font: Italic

~~upon, as the differences were not equal or greater than two AIC units lower than another. Similarly, within the EQ models, the life history model had the lowest AIC score, however this was not significantly different to the combined model (with a difference of 0.06). Interestingly, in both cerebellum inputs, the social models had the lowest AIC scores; however similarly to the other carnivore results, the scores were within two AIC units of another model. In contrast to the primate data, generally there is no significant difference between the ecological and social models, excluding the cerebellum models where social models have significantly improved AIC scores.~~

Table 3 about here

Overall encephalisation

The results of PGLS analysis on endocranial volume data are presented in Table 3, with the '*best fit*' models ~~presented~~shown in Table 4. The variables which were indicated to be of importance and included within the '*best fit*' endocranial volume models were: fertility, dietary breadth, maximum longevity and age at first reproduction. After accounting for phylogeny, fertility was found to be negatively associated with ECV (P <0.05), with this being the only variable significantly associated with endocranial volume. For example, dietary breadth was close to being negatively associated with ECV, but ~~this~~ fell short of significance (P = 0.05). In addition, both maximum lifespan and age at first reproduction, failed to ~~find~~reach significance (P = 0.08, P = 0.10).~~, relative brain size and encephalisation quotient~~

Formatted: Font: Italic

Formatted: Font: Italic

~~are shown in Table 4. After taking phylogeny into account, dietary breadth was found to be negatively associated with endocranial volume (P <0.05). Two life-history variables were also significantly correlated with whole brain measures; fertility was negatively correlated with ECV (P <0.05), RBS (P <0.01) and EQ (P <0.05), as well as weaning age which was negatively correlated with EQ (P <0.05). Dietary breadth was close to being significantly correlated with RBS; however, this association fell short of significance (P = 0.07).~~

Table 4 about here

Regional brain volumes

The results of PGLS analysis on the neocortex and cerebellum data are presented in Table 3, with the '*best fit*' models ~~presented~~shown in Table 4. The variables which were indicated to be of importance and included ~~with~~in the '*best fit*' neocortex models were: age at first reproduction, maximum lifespan and home range size. After accounting for phylogeny, age at first reproduction was found to be positively associated with neocortex (P <0.001), with this being the only variable significantly associated with neocortex volume. For example, home range size was close to being positively associated with neocortex volume, but~~ this~~ fell~~t~~ short of significance (P = 0.07). In addition, maximum lifespan failed to ~~find~~reach significance (P = 0.19). ~~The results of PGLS analysis on the neocortex and cerebellum models are also presented in Table 4. After taking phylogeny into account, social cohesion was found to be negatively correlated with both relative cerebellum and cerebellum volume (P~~

Formatted: Font: Italic

Formatted: Font: Italic

~~<0.05). Similar to whole brain measures, fertility and weaning age were found to be negatively associated with relative neocortex and neocortex volume (P <0.001, P <0.05 respectively), as well as age of first reproduction, which was also negatively correlated with RNS (P <0.05).~~

The variables which were indicated to be of importance and included within the '*best fit*' cerebellum models were: home range size, gestation length, maximum lifespan and age at first reproduction. Also present within the subset of '*best fit*' models were: different iterations of the previously mentioned variables and weaning age. After accounting for phylogeny, home range size was found to be significantly associated with cerebellum volume (P <0.05). Three of the life-history variables were significantly associated with cerebellum volume: gestation length, maximum lifespan and age at first reproduction (P <0.05, P <0.01, P <0.001). Although, home range size and gestation length ~~did~~ failed to ~~find~~reach significance in certain model iterations (P = 0.08, P = 0.07). Weaning age also failed to ~~find~~reach significance (P = 0.10).

Discussion

~~Using~~ Applying robust statistical analyses, a recently updated phylogenetic tree, ~~substantial sample sizes~~a comprehensive dataset~~,~~ and models of varying complexity, the correlates of brain size in primates and carnivores were reconsidered. Consistent associations were found between brain size and ecological variables in primates, thus highlighting the influence of ecology on encephalisation. However, support was also found for the prominent social brain

hypothesis~~, with the cerebellum appearing to be of importance for social intelligence~~, specifically revealing evidence for a link between whole brain volumes and two measures of sociality. ~~.~~ In carnivores, data suggest ~~both~~ ecological ~~and social~~ variables shape brain size, suggesting alternative evolutionary patterns influencing carnivoran encephalisation. In both groups, life history variables appear crucial in counterbalancing the costs of producing and maintaining increased brain size, through extended developmental periods, reduced fertility and increased maximum lifespan.

Primates

Here, consistent with current literature, robust correlations were found between brain size and ecological variables. The most prominent of these were diet related, ~~;~~ with dietary categories or dietary breadth appearing in all~~-~~ ‘*best fit*’ models, for both whole brain and regional brain data. ~~The most prominent of these were diet related; diet being the most consistent ecological correlate, with the relationship holding across multiple models, even when using different brain size calculations.~~ These findings are similar to those of DeCasien et al., (20)~~(2017)~~ and Powell et al., (14)~~(2017)~~ who ~~both~~ found stronger and more consistent associations with ecological variables than those related to the social environment. Akin to the result of DeCasien et al. (20)~~(2017)~~, ~~strong~~ support was found for~~-~~ omnivory~~frugivory~~, as well as ~~omnivory~~frugivory, as ~~key~~ correlates of brain size. However, in contrast ~~with~~to the literature, here the correlations between regional brain volumes and dietary categories, were negatively correlated. This perhaps reflects both the need to sustain the energetic cost of brain tissue (highlighted by ~~Aiello & Wheeler, 1995~~ (115); (116)~~Fish & Lockwood, 2003~~), as well as meeting the cognitive foraging challenges imposed by ~~frugivorous and~~ omnivorous and frugivorous diets (3)~~(Milton, 1981) which ultimately leads to increased~~

Formatted: Font: Italic

encephalisation. . In addition to the diet categories, dietary breadth was significantly (positively) correlated with all whole brain measureswhole brain volumes, further reinforcing the suggestion proposition that diet influences brain size, whilst highlighting how useful this proxy can be in understanding how availability and variety of food sources can be important in setting the cognitive challenge. For example, MacLean et al. (50)(2014) also suggested dietary breadth to be an important ecological correlate, with greater cognitive flexibility allowing individuals to explore and exploit new food sources, as well as use deploy extractive foraging techniques. Evidence for associations between regional brain size volumes and home range size were also found (see supplementary material), supporting the view of Powell et al. (2017)(14) in that certain dietary categories, such as frugivory, may covary with home range. Similar results were also found by Graber et al., (117)(2017). Interestingly, alongside the home range size associations found here, habitat variability was negatively correlated with brain size, possibly suggesting there is also importance in the habitat type used by a species.

In the past considerable support indicated that sociality was the major driver of encephalisation in primates. More recent works, however, conflict with contest this long-held viewpoint, failing to find support for a link between brain size and sociality measures (14, 19, 20, 50, 51)(Swanson et al., 2012; MacLean et al., 2014; van Schaik et al., 2016; Powell et al., 2017; DeCasien et al., 2017). Our findings, however, contrast with current research, confirming support for the social brain hypothesis. Here, our models revealed evidence of a link between brain size and sociality in primates. This association was present only in the whole brain '*best fit*' models, with both variables reaching significance, indicating both increasing social group size and varying levels of social cohesion are influencing brain size in primates. The most robust associations were those found in the cerebellum models,

Formatted: Font: Italic

~~which is consistent with research that highlights the importance of the cerebellum in social intelligence (Barton, 2012); however, further associations were also found (see supplementary material).~~ Interestingly, use of the social cohesion proxy was ~~mostly~~ often preferred when comparing models, thereby suggesting the use of this proxy is superior when testing multiple ecological and social variables simultaneously. The inference too is that there may be greater importance in relationship quality, over quantity, as suggested by past research into primate sociality and pair-bonds (34, 45, 49, 95, 118)~~(Shultz & Dunbar, 2007, 2017; Layton & O'Hara, 2010; Silk, 2012; Bergman & Beehner, 2015)~~. It is importan~~t~~~~ce~~ to note however, that whilst there was support for this hypothesis, ecological models were preferrable over social ones~~,~~ and~~,~~ ecological variables appear to be more robust correlates of brain size when compared to measures of sociality (see (117)).

Consistent with the literature, support was found for correlations between life-history variables and brain size. As suggested within the developmental cost (110)~~(Barton & Capellini, 2011)~~ and maternal energy (119)~~(Martin, 1996)~~ hypotheses, relationships found possibly reflect the developmental costs associated with growing large brains, which appear to be bypassed through extended developmental periods and increased maternal investment (120, 121)~~(Heldstab et al., 2019; Isler & van Schaik, 2009)~~. Similarly, Powell et al. (105)~~(2019)~~ found correlations between neocortex volume ~~brain volumes~~ and gestation length, as well as cerebellum volume and juvenile period. Whilst the associations found here differ in terms of the specific regions involved, ~~Additionally, findings are consistent with the expensive brain hypothesis (Isler & van Schaik, 2009), which proposes either an increase in energy turnover or a reduction in energy allocation is needed in order to meet the costs of increased brain size. This is seen here by the negative fertility correlation, suggesting a reduction in reproductive output.~~ t~~T~~his supports the theory as to why relatively large-

brained mammals often exhibit slow maturation times and reduced fertility; thus, by increasing developmental periods and maternal investment, primates possess these slow life histories which ultimately facilitates the production of big brains. This therefore makes the ‘extended parenting’ association critical to the evolution of cognition (90, 120, 122, 123)~~(Isler & van Schaik, 2012; Heldstab et al., 2019, 2020; Uomini et al., 2020)~~. However, one mystery still left to solve is the reasoning behind the association found here between brain size and maximum longevity. One proposition ~~suggests~~ is that selection mechanisms work towards counterbalancing ~~mammals counterbalance~~ the costs of large brains in mammals with a longer reproductive lifespan (124)~~(González-Lagos et al., 2010)~~, ~~-~~ and thus, by extending the reproductive lifespan of a species, it counteracts the time and effort spent producing and maintaining large brains~~,~~ and aims to maximise the time species can spend producing young, which in turn have large brains. Whereas others propose ~~whilst others propose~~ the correlation is indirect and that a longer reproductive lifespan is a by-product of shifting developmental and maturation periods (105)~~(Powell et al., 2019)~~.

Carnivores

Akin to the primate results, ~~here~~for carnivores, support is found for a link between regional brain volumes and home range size ~~in carnivores~~. This relationship reached significance in the cerebellum models, concurring with research suggesting this region is important for spatial memory processing (1, 125, 126). Simply, larger home range sizes are thought to require the use of complex information about food location and distribution (9), which for example in carnivores, may represent the challenges of locating travelling herds of herbivores. Alongside this association, indicating spatial demands influence brain size in carnivores, dietary breadth was another ecological variable included ~~with~~in the ‘*best fit*’

Formatted: Font: Italic

endocranial volume models. However, in contrast to the results of MacLean et al., (50) and Swanson et al., (19), the relationship between dietary breadth and brain size is negatively directed,~~;~~ suggesting greater dietary breadth is actually associated with smaller brain size in carnivores. ~~Affirming the contemporary viewpoint, here, support is found for a link between brain size and diet in carnivores. However, in contrast to the results of MacLean et al., (2014) and Swanson et al., (2012), the relationship between brain size and dietary breath is negatively correlated; contradicting the assertion that dietary generalists possess larger brains and superior skillsets than those more specialised or with more limited food variety. For example, dietary generalists in birds have been found to show more technical innovations and possess larger brains when compared to dietary specialists (Ducatez, Clavel & Lefebvre, 2014; Shultz et al., 2005). Yet, our findings indicate greater dietary breadth is actually associated with smaller brain size in carnivores.~~ This result could perhaps be a consequence of those species who are classified as obligate meat eaters, whose dietary breath is limited to one or two categories, thereby producing this negative correlation. Despite this, obligate meat-eating carnivores consume the highest caloric diet, which is thought to provide greater energy for producing large brains. This highlights how carnivores cannot simply be compared and likened to other mammalian orders, such as Primates, and suggests different evolutionary mechanisms at work in carnivoran lineages. It is important to note, however, that this association, whilst close to, failed to ~~find~~reach significance (P = 0.05), suggesting this relationship is not a strong influence on brain size in carnivores. ~~Additional ecological associations were found specifically related to habitat variables (see supplementary material), with those associations suggesting spatial demands also influence carnivoran brain size.~~

Whilst previous work has suggested sociality plays a role in the evolution of brain size in carnivoran lineages (31, 33-35)~~(Holekamp et al., 2015; Shultz & Dunbar, 2007; Pérez-Barbería et al., 2007; Dunbar & Bever, 1998)~~, here, we find ~~the relationship between brain size and sociality is negatively correlated~~no support for a link between measures of sociality and brain size in carnivores. Similarly, MacLean et al., (50)~~(2014)~~, Benson-Amram et al., (127)~~(2016)~~, and Swanson et al., ~~(2012)~~(19) found no support for the social brain hypothesis in mammals. The contrasting results present in the literature could be due to the fact that sociality appears to be limited to a select few carnivore taxa, specifically social species from the families Hyaenidae, Procyonidae and Felidae (128)~~(Sakai & Arsznov, 2020)~~. This is suggested in the findings of Finarelli & Flynn (55)~~(2009)~~, who identified that support for the SBH in Carnivora was dependent on data from Canidae, without which, no association is found. Thus, whilst sociality evidently plays an important role in primates, leading to complex, multi-faceted societies, this is less common in carnivore species, and therefore does not hold the same importance. ~~Interestingly, just as was found in the primate models, the influence of sociality was restricted to the cerebellum, further suggesting it is this brain region that is predominately underpinning the management of social interactions.~~

Consistent with the previously discussed primate results, associations were found between life-history variables and brain size in carnivores. Age at~~of~~ first reproduction, gestation length and maximum lifespan were all found to positively correlate with regional brain volumes, suggesting both an increase in developmental periods as well as an extension in reproductive lifespans. Additionally, findings are consistent with the expensive brain hypothesis (121), which proposes either an increase in energy turnover or a reduction in energy allocation is needed in order to meet the costs of increased brain size. This is seen here ~~by~~ with a negative ~~fertility~~ correlation ~~with~~between fertility and endocranial volume,

suggesting a reduction in reproductive output. ~~most specifically the expensive brain hypothesis (Isler & van Schaik, 2009), fertility, age at first reproduction and weaning age were found to be negatively associated with brain size. This result reinforces support for the aforementioned hypothesis, confirming the proposition that in order to develop and sustain a large brain, there must be a trade-off with another expensive function, which in this case is reproduction.~~ This, when paired with an increase in maternal investment and developmental periods, as suggested by ~~-~~ the aforementioned results~~the primate results~~, bypasses the developmental constraints of producing a large brain through reduced fertility and slow maturation times.

Whole versus regional brain volumes~~Brain size confusion~~

~~Choosing the suitable body size correction factor for use in studies of brain evolution has been highlighted as a complex problem (van Schaik et al., 2021; Swanson et al., 2012). The disparity in results of comparative analyses of brain evolution could be in part the result of the use of different correction measures. For example, earlier studies used residuals from regression of brain size on body mass, whereas more recent studies are often scaled using residuals from regression of brain size on another brain structure or are taken from ratios (Deaner et al., 2000). The body size correction methods here produced similar outputs. However, some differences were present; for example, in primates, the association between habitat variability and EQ, which failed to remain present when using other methods. This highlights numerous concerns. First, without the inclusion of this method, the association would have been missed. Second, it raises questions regarding the validity of the association. Without clarity regarding the most suitable correction measure, it is difficult to~~

~~ascertain which associations are 'true' correlates and which do not actually invoke influential change in brain evolution but are rather the consequence of inaccurate correction methods. This confusion regarding correction measures needs addressing, with the aim of determining the superior method, allowing greater clarity on past and current research whilst guiding future comparative analyses. For example, the fact that most carnivore models using residuals or other statistical calculations failed to be significant is noteworthy. Residuals appear to fail to appropriately account for body size in carnivores, or rather, as previously mentioned, brain estimates appear to be a poor representation of carnivoran brains due to the fact that carnivore brain size shows a lag relative to body size over evolutionary time (Swanson et al., 2012). Thus, inputting body size into a model as a covariate, rather than using any other brain estimate, appears most appropriate when designing comparative analyses of carnivoran brain evolution.~~

Our study highlights the benefit of investigating both whole brain and regional brain volumes. Whole brain volumes are often more readily available for species and thus by choosing to use this brain measure it increases sample sizes and commensurate statistical power. In addition, it has been argued the neocortex comprises a large proportion of whole brain volume, making the two brain volumes closely related (34, 95)~~(Shultz & Dunbar, 2007; Dunbar & Shultz, 2017)~~. However, it is possible the inclusion of specific brain regions may uncover further associations that were not significant or present before. This was the case here, where for primates, the home range association only became significant in the neocortex and cerebellum models, ~~and failed to~~having not reached significance~~t~~ in endocranial volume models. Additionally, in carnivores, many of the life-history associations, for example age at first reproduction, ~~were~~ only reached significance~~t~~ in the regional brain volume models. Therefore, without investigating specific brain regions, the

influence of these associations would have been missed. ~~This is the case here, where for primates, social cohesion was close to significance in whole brain volume models but only reached significance in the cerebellum models. Without including this brain region, the influence of this association would have been missed.~~ In addition to this, the use of whole brain size does not necessarily allow the study of the ways in which different selective pressures act on different neural systems, as proposed by theories of mosaic evolution (5, 61)~~(Barton & Harvey, 1995; Barton & Harvey, 2000)~~. This often makes it difficult to relate whole brain size to individual selection pressures (129)~~(Healy & Rowe, 2007)~~. ~~Therefore,~~ B~~b~~y investigating specific brain regions, where brain data and the corresponding covariates are available, it allows the further analysis of how multiple functional systems can evolve in a mosaic fashion in response to different selection pressures.

Conclusion

To conclude, the evidence presented here supports the proposition that ecological variables hold greater influence in determining brain size in primate lineages. However, critical support is also found for the SBH in primates, confirming sociality does hold significance in encephalisation. Ecological variables, most notably home range size, appear to shape~~ing~~ carnivoran brain size~~ in carnivores~~. Yet no support is found there for measures of sociality, indicating that sociality may not hold the same importance within that~~is~~ order. ~~Multiple variables appear to be shaping brain size in carnivores, including both ecological and social variables, which requires greater investigation to unpick.~~ Life-history traits reveal evidence for the transition to slow life histories, which work toward facilitating the production of big brains and bypassing the cost of expensive brain tissue.~~ The use of different body size correction methods is found to produce disparate results, which potentially hampers the~~

~~validity of correlates of brain size, as without any indication of the most appropriate measure, there is little clarity as to which associations reflect 'true' evolutionary influence.~~ Whilst data availability limits the application of comparative studies of brain evolution in many species, future studies should strive to integrate multiple variables, fully encompassing all the potential variables influencing brain size. In addition, where possible, researchers should investigate both whole brain and specific brain regions, as the inclusion of such may reveal further associations, capturing how different brain regions can evolve independently through varying selection pressures.

Acknowledgements

We thank Alex DeCasien for help regarding encephalisation quotients and model comparisons analyses. We are grateful to Eli Swanson for valuable discussions regarding PGLS analysis and for providing additional data analysis resources. Our thanks also to F. Sayol, O. Bertrand, V. Weisbecker, N. Emery, M. Tucker, D. Hinchcliffe, M. Olalla-Tárraga, S. Shultz, J. Gundry, C. O'Hara and C. Fauvelle. We are further grateful to the reviewers, whose thorough examinations led to a range of helpful suggestions that greatly assisted us in improving our ms.

Data Accessibility: All data supporting the findings of this study are available in the paper and the supplementary information.

Author Contributions: conceptualisation and methodology, HRC and SOH; investigation and data curation, HRC; formal analysis, HRC, SAH; supervision, SOH; project administration, HRC; visualisation and original draft, HRC, SOH; review and editing, HRC, SOH, SAH.

Funding: This research received no external funding

Conflict of interest: Authors declare no competing interests

References

~~Aiello, L.C., Wheeler, P. (1995). The expensive-tissue hypothesis: the brain and the digestive system in human and primate evolution. *Current Anthropology, 36*(2), 199-221. Doi: 10.1086/204350.~~

~~Akaike, H (1974). A new look at the statistical model identification. *IEEE Transactions on Automatic Control, 19*(6), 716-723. Doi: 10.1109/TAC.1974.1100705.~~

~~Barrickman, N.L., Bastian, M.L., Isler, K., van Schaik, C.P. (2008). Life history costs and benefits of encephalization: a comparative test using data from long-term studies of primates in the wild. *Journal of Human Evolution, 54*(5), 568-590. Doi: 10.1016/j.jhevol.2007.08.012.~~

~~Barton, R.A. (2000). Primate brain evolution: cognitive demands of foraging or of social life? In S. Boinski & P.A. Garber (Eds.), *On the Move: How and Why Animals Travel in Groups* (pp. 204-237). London: University of Chicago Press.~~

~~Barton, R.A. (2002). How did brains evolve? *Nature, 415*, 134-135. Doi: 10.1038/415134a.~~

Barton, R.A. (2012). Embodied cognitive evolution and the cerebellum. *Philosophical Transactions of the Royal Society B: Biological Sciences, 367*(1599), 2097-2107. Doi: 10.1098/rstb.2012.0112.

Barton, R.A., Capellini, I. (2011). Maternal investment, life histories, and the costs of brain growth in mammals. *PNAS, 108*(15), 6169-6174. Doi: 10.1073/pnas.1019140108.

Barton, R.A., Harvey, P. (2000). Mosaic evolution of brain structure in mammals. *Nature, 405*, 1055-1058. Doi: 10.1038/35016580.

Barton, R.A., Purvis, A., Harvey, P.H. (1995). Evolutionary radiation of visual and olfactory brain systems in primates, bats and insectivores. *Philosophical Transactions of The Royal Society B: Biological Science, 348*(1326), 381-392. Doi: 10.1098/rstb.1995.0076.

Barton, R.A., Venditti, C. (2014). Rapid evolution of the cerebellum in humans and other great apes. *Current Biology, 24*(20), 2440-2444. Doi: 10.1016/j.cub.2014.08.056.

Benson-Amram, S., Dantzer, B., Stricker, G., Swanson, E.M., Holekamp, K.E. (2016). Brain size predicts problem-solving ability in mammalian carnivores. *PNAS, 113*(9), 2532-2537. Doi: 10.1073/pnas.1505913113.

Bergman, T.J., Beehner, J.C. (2015). Measuring social complexity. *Animal Behaviour, 103*, 203-209. Doi: 10.1016/j.anbehav.2015.02.018.

Bshary, R. (2011). Machiavellian intelligence in fishes. In C. Brown, K. Laland & J. Krause (Eds.), *Fish Cognition and Behavior* (pp. 277-297). Chichester: Blackwell Publishing Ltd.

Byrne, R.W., Bates, L.A. (2007). Brain evolution: when is a group not a group? *Current Biology, 17*(20), 883-884. Doi: 10.1016/j.cub.2007.08.018.

Byrne, R.W., Corp, N. (2004). Neocortex size predicts deception rate in primates. *Proceedings of the Royal Society B: Biological Sciences, 271*(1549), 1693-1699. Doi: 10.1098/rspb.2004.2780.

Byrne, R.W., Whiten, A. (1988). *Machiavellian intelligence: social expertise and the evolution of intellect in monkeys, apes, and humans*. New York: Oxford University Press.

Cantania, K.C. (2004). Correlates and possible mechanisms of neocortical enlargement and diversification in mammals. *International Journal of Comparative Psychology, 17*(1), 71-91.

Clutton-Brock, T.H., Harvey, P.H. (1980). Primates, brains and ecology. *Journal of Zoology, 190*(3), 309-323. Doi: 10.1111/j.1469-7998.1980.tb01430.x.

Deaner, R.O., Isler, K., Burkart, J., van Schaik, C.P. (2007). Overall brain size, and not encephalization quotient, best predicts cognitive ability across non-human primates. *Brain, Behavior and Evolution, 70*(2), 115-124. Doi: 10.1159/000102973.

Deaner, R.O., Nunn, C.L., van Schaik, C.P. (2000). Comparative tests of primate cognition: different scaling methods produce different results. *Brain, Behavior and Evolution, 55*(1), 44-52. Doi: 10.1159/000006641.

DeCasien, A.R., Higham, J.P. (2019). Primate mosaic brain evolution reflects selection on sensory and cognitive specialization. *Nature Ecology & Evolution, 3*(10), 1483-1493. Doi: 10.1038/s41559-019-0969-0.

DeCasien, A.R., Thompson, N.A., Williams, S.A., Shattuck, M.R. (2018). Encephalization and longevity evolved in a correlated fashion in Euarchontoglires but not in other mammals. *Evolution, 72*(12), 2617-2631. Doi: 10.1111/evo.13633.

DeCasien, A.R., Williams, S.A., Higham, J.P. (2017). Primate brain size is predicted by diet but not sociality. *Nature Ecology & Evolution, 1*(0112), 1-7. Doi: 10.1038/s41559-017-0112.

Ducatez, S., Clavel, J., Lefebvre, L. (2014). Ecological generalism and behavioural innovation in birds: technical intelligence or the simple incorporation of new foods? *Journal of Animal Ecology, 84*(1), 79-89. Doi: 10.1111/1365-2656.12255.

Dunbar, R.I.M. (1992). Neocortex size as a constraint on group size in primates. *Journal of Human Evolution, 22*(6), 469-493. Doi: 10.1016/0047-2484(92)90081-J.

Dunbar, R.I.M. (1998). The social brain hypothesis. *Evolutionary Anthropology, 6*(5), 178-190. Doi: 10.1002/(SICI)1520-6505(1998)6:5<178::AID-EVAN5>3.0.CO;2-8.

Dunbar, R.I.M. (2009). The social brain hypothesis and its implications for social evolution. *Annals of Human Biology, 36*(5), 562-572. Doi: 10.1080/03014460902960289.

Dunbar, R.I.M., Bever, J. (1998). Neocortex size predicts group size in carnivores and some insectivores. *Ethology, 104*(8), 695-708. Doi: 10.1111/j.1439-0310.1998.tb00103.x.

Dunbar, R.I.M., Shultz, S. (2007a). Evolution in the social brain. *Science, 317*(5843), 1344-1347. Doi: 10.1126/science.1145463.

Dunbar, R.I.M., Shultz, S. (2007b). Understanding primate brain evolution. *Philosophical Transactions of the Royal Society B: Biological Sciences, 362*(1480), 649-658. Doi: 10.1098/rstb.2006.2001.

Dunbar, R.I.M., Shultz, S. (2017). Why are there so many explanations for primate brain evolution? *Philosophical Transactions of the Royal Society B: Biological Sciences, 372*(1727), 20160244. Doi: 10.1098/rstb.2016.0244.

Emery, N.J., Seed, A.M., von Bayern, A.M.P., Clayton, N.S. (2007). Cognitive adaptations of social bonding in birds. *Philosophical Transactions of the Royal Society B: Biological Sciences, 362*(1480), 489-505. Doi: 10.1098/rstb.2006.1991.

Fernandes, H.B.F., Peñaherrera-Aguirre, M., Woodley of Menie, M.A., Figueredo, A.J. (2020). Macroevolutionary patterns and selection modes for general intelligence (G) and for commonly used neuroanatomical volume measures in primates. *Intelligence, 80*, 101456. Doi: 10.1016/j.intell.2020.101456.

Finarelli, J.A., Flynn, J.J. (2009). Brain-size evolution and sociality in Carnivora. *PNAS, 106*(23), 9345-9349. Doi: 10.1073/pnas.0901780106.

Finlay, B.L., Darlington, R.B. (1995). Linked regularities in the development and evolution of mammalian brains. *Science, 268*(5217), 1578-1584. Doi: 10.1126/science.7777856.

Finlay, B.L., Darlington, R.B., Nicastro, N. (2001). Developmental structure in brain evolution. *Behavioral and Brain Sciences, 24*(2), 263-278. Doi: 10.1017/S0140525X01003958.

Fish, J.L., Lockwood, C.A. (2003). Dietary constraints on encephalization in primates. *American Journal of Physical Anthropology, 120*(2), 171-181. Doi: 10.1002/ajpa.10136.

Freckleton, R.P. (2002). On the misuse of residuals in ecology: regression of residuals vs. multiple regression. *Journal of Animal Ecology, 71*(3), 542-545. Doi: 10.1046/j.1365-2656.2002.00618.x.

Gibson, K.R. (1986). Cognition, brain size and the extraction of embedded food resources. In J.G. Else & P.C. Lee (Eds.), *Primate ontogeny, cognition and social behaviour* (pp. 93-103). Cambridge: Cambridge University Press.

González-Lagos, C., Sol, D., Reader, S.M. (2010). Large-brained mammals live longer. *Journal of Evolutionary Biology, 23*(5), 1064-1074. Doi: 10.1111/j.1420-9101.2010.01976.x.

Gonzalez-Voyer, A., Winberg, S., Kolm, N. (2008). Social fishes and single mothers: brain evolution in African cichlids. *Proceedings of the Royal Society B: Biological Sciences, 276*(1654), 161-167. Doi: 10.1098/rspb.2008.0979.

Graber, M.S. (2017). *Social and ecological aspects of brain size evolution: a comparative approach* (PhD Thesis). University of Zurich, Zurich. Retrieved from: https://www.zora.uzh.ch/id/eprint/146749/.

Healy, S.D., Rowe, C. (2007). A critique of comparative studies of brain size. *Proceedings of the Royal Society B: Biological Sciences, 274*(1609), 453-464. Doi: 10.1098/rspb.2006.3748.

Heldstab, S.A., Isler, K., Burkart, J.M., van Schaik, C.P. (2019). Allomaternal care, brains and fertility in mammals: who cares matters. *Behavioural Ecology and Sociobiology, 73*(6), 71. Doi: 10.1007/s00265-019-2684-x.

Heldstab, S.A., Isler, K., Schuppli, C., van Schaik, C.P. (2020). When ontogeny recapitulates phylogeny: Fixed neurodevelopmental sequence of manipulative skills among primates. *Science Advances, 6*(30), eabb4685. Doi: 10.1126/sciadv.abb4685.

Heldstab, S.A., Kosonen, Z.K., Koski, S.E., Burkart, J.M., van Schaik, C.P., Isler, K. (2016). Manipulation complexity in primates coevolved with brain size and terrestriality. *Scientific Reports, 6*, 24528. Doi: 10.1038/srep24528.

Holekamp, K.E. (2007). Questioning the social intelligence hypothesis. *Trends in Cognitive Sciences, 11*(2), 65-69. Doi: 10.1016/j.tics.2006.11.003.

Holekamp, K.E., Benson-Amram, S. (2017). The evolution of intelligence in mammalian carnivores. *Interface Focus, 7*(3), 20160108. Doi: 10.1098/rsfs.2016.0108.

Holekamp, K.E., Dantzer, B., Stricker, G., Shaw Yoshida, K.C., Benson-Amram, S. (2015). Brains, brawn and sociality: a hyaena's tale. *Animal Behaviour, 103*, 237-248. Doi: 10.1016/j.anbehav.2015.01.023.

Innocenti, G.M., Kaas, J.H. (1995). The cortex. *Trends in Neurosciences, 18*, 371-372.

Isler, K., van Schaik, C.P. (2014). How humans evolved large brains: comparative evidence. *Evolutionary Anthropology, 23*(2), 65-75. Doi: 10.1002/evan.21403.

Isler, K., Kirk, C.E., Miller, J.M.A., Albrecht, G.A., Gelvin, B.R., Martin, R.D. (2008). Endocranial volumes of primate species: scaling analyses using a comprehensive and reliable data set. *Journal of Human Evolution, 55*(6), 967-978. Doi: 10.1016/j.jhevol.2008.08.004.

Isler, K., van Schaik, C.P. (2009). The expensive brain: a framework for explaining evolutionary changes in brain size. *Journal of Human Evolution, 57*(4), 392-400. Doi: 10.1016/j.jhevol.2009.04.009.

Isler, K., van Schaik, C.P. (2012). Allomaternal care, life history and brain size evolution in mammals. *Journal of Human Evolution, 63*(1), 52-63. Doi: 10.1016/j.jhevol.2012.03.009.

IUCN Red List. (2020). The IUCN Red List of Threatened Species. Retrieved from: https://www.iucnredlist.org/.

Jerison, H.J. (1973). *Evolution of the brain and intelligence*. Academic Press: New York.

Kaas, J.H. (1995). The evolution of isocortex. *Brain, Behavior and Evolution, 46*, 187-196. Doi: 10.1159/000113273.

Kudo, H., Dunbar, R.I.M. (2001). Neocortex size and social network size in primates. *Animal Behaviour, 62*(4), 711-722. Doi: 10.1006/anbe.2001.1808.

Layton, R.A., O'Hara, S.J. (2010). Human social evolution: a comparison of hunter-gatherer and chimpanzee social organisation. In R. Dunbar, C. Gamble & J. Gowlett (Eds.), *Social brain, distributed mind* (pp. 85-115). Oxford: British Academy.

MacLean, E.L., Hare, B., Nunn, C.L., Addessi, E., Amici, F., Anderson, R.C. … Zhao, Y. (2014). The evolution of self-control. *PNAS, 111*(20), E2140-E2148. Doi: 10.1073/pnas.1323533111.

Martin, R.D. (1996). Scaling of the mammalian brain: the maternal energy hypothesis. *Physiology, 11*(4), 149-156. Doi: 10.1152/physiologyonline.1996.11.4.149.

Milton, K. (1981). Distribution patterns of tropical plant foods as an evolutionary stimulus to primate mental development. *American Anthropologist, 83*(3), 534-548. Doi: 10.1525/aa.1981.83.3.02a00020.

Parker, S.T. (2015). Re-evaluating the extractive foraging hypothesis. *New Ideas in Psychology, 37*, 1-12. Doi: 10.1016/j.newideapsych.2014.11.001.

Parker, S.T., Gibson, K.R. (1977). Object manipulation, tool use and sensorimotor intelligence as feeding adaptations in cebus monkeys and great apes. *Journal of Human Evolution, 6*(7), 623-641. Doi: 10.1016/S0047-2484(77)80135-8.

Pérez-Barbería, F.J. Gordon, I.J. (2005). Gregariousness increases brain size in ungulates. *Oecologia, 145*(1), 41-52. Doi: 10.1007/s00442-005-0067-7.

Pérez-Barbería, F.J., Shultz, S., Dunbar, R.I.M. (2007). Evidence for coevolution of sociality and relative brain size in three orders of mammals. *Evolution, 61*(12), 2811-2821. Doi: 10.1111/j.1558-5646.2007.00229.x.

Plante, S., Colchero, F., Calme, S. (2014). Foraging strategy of a neotropical primate: how intrinsic and extrinsic factors influence destination and residence time. *Journal of Animal Ecology, 83*, 116-125. Doi: 10.1111/1365-2656.12119.

Powell, J., Lewis, P.A., Roberts, N., García-Fiñana, M., Dunbar, R.I.M. (2012). Orbital prefrontal cortex volume predicts social network size: an imaging study of individual differences in humans. *Proceedings of the Royal Society B: Biological Sciences, 279*(1736), 2157-2162. Doi: 10.1098/rspb.2011.2574.

Powell, L.E., Barton, R.A., Street, S.E. (2019). Maternal investment, life histories and the evolution of brain structure in primates. *Proceedings of the Royal Society B: Biological Sciences, 286*(1911), 20191608. Doi: 10.1098/rspb.2019.1608.

Powell, L.E., Isler, K., Barton, R.A. (2017). Re-evaluating the link between brain size and behavioural ecology in primates. *Proceedings of the Royal Society B: Biological Sciences, 284*(1865), 20171765. Doi: 10.1098/rspb.2017.1765.

Reader, S.M., Hager, Y., Laland, K.N. (2011). The evolution of primate general and cultural intelligence. *Philosophical Transactions of the Royal Society B: Biological Sciences, 366*(1567), 1017-1027. Doi: 10.1098/rstb.2010.0342.

Sakai, S.T., Arsznov, B.M. (2020). Carnivoran brains: effects of sociality on inter and intraspecific comparisons of regional brain volumes. In J.H. Kaas (Ed.), *Evolutionary Neuroscience* (pp. 463-479). London: Academic Press.

Sakai, S.T., Arsznov, B.M., Hristova, A.E., Yoon, E.J., Lundrigan, B.L. (2016). Big cat coalitions: a comparative analysis of regional brain volumes in felidae. *Frontiers in Neuroanatomy, 10*, 99. Doi: 10.3389/fnana.2016.00099.

Sakai, S.T., Arsznov, B.M., Lundrigan, B.L., Holekamp, K.E. (2011). Brain size and social complexity: a computed tomography study in hyaenidae. *Brain, Behavior and Evolution, 77*(2), 91-104. Doi: 10.1159/000323849.

Sallet, J., Mars, R.B., Noonan, M.P., Andersson, J.L., O'Reilly, J.X., Jbabdi, S., … Rushworth, M.F.S. (2011). Social network size affects neural circuits in macaques. *Science, 334*(6056), 697-700. Doi: 10.1126/science.1210027.

Scheiber, I.B.R., Weib, B.M., Hirschenhauser, K., Wascher, C.A.F., Nedelcu, I.T., Kotrschal, K. (2008). Does 'relationship intelligence' make big brains in birds? *The Open Biology Journal, 1*, 6-8. Doi: 10.2174/1874196700801010006.

Shultz, S. Dunbar, R.I.M. (2006). Both social and ecological factors predict ungulate brain size. *Proceedings of the Royal Society B: Biological Sciences, 273*(1583), 207-215. Doi: 10.1098/rspb.2005.3283.

Shultz, S. Dunbar, R.I.M. (2010). Encephalization is not a universal macroevolutionary phenomenon in mammals but is associated with sociality. *PNAS, 107*(50), 21582-21586. Doi: 10.1073/pnas.1005246107.

Shultz, S., Bradbury, R.B., Evans, K.L., Gregory, R.D., Blackburn, T.M. (2005). Brain size and resource specialization predict long-term population trends in British birds. *Proceedings of*

the Royal Society B: Biological Sciences, 272(1578), 2305-2311. Doi: 10.1098/rspb.2005.3250.

Shultz, S., Dunbar, R.I.M. (2007). The evolution of the social brain: anthropoid primates contrast with other vertebrates. *Proceedings of the Royal Society B: Biological Sciences, 274*, 2429-2436. Doi: 10.1098/rspb.2007.0693.

Shultz, S., Dunbar, R.I.M. (2010). Social bonds in birds are associated with brain size and contingent on the correlated evolution of life-history and increased parental investment. *Biological Journal of the Linnean Society, 100*(1), 111-123. Doi: 10.1111/j.1095-8312.2010.01427.x.

Silk, J.B. (2012). The adaptive value of sociality. In J. C. Mitani et al. (Eds.), *The Evolution of Primate Societies* (pp. 552-564). Chicago: The University of Chicago Press.

Smaers, J.B., Dechmann, D.K.N., Goswami, A., Soligo, C., Safi, K. (2012). Comparative analyses of evolutionary rates reveal different pathways to encephalization in bats, carnivorans, and primates. *PNAS, 109*(44), 18006-18011. Doi: 10.1073/pnas.1212181109.

Smaers, J.B., Turner, A.H., Gomez-Robles, A., Sherwood, C.C. (2018). A cerebellar substrate for cognition evolved multiple times independently in mammals. *eLife, 7*, e35696. Doi: 10.7554/eLife.35696.

Smaers, J.B., Vanier, D.R. (2019). Brain size expansion in primates and humans is explained by a selective modular expansion of the cortico-cerebellar system. *Cortex, 118*, 292-305. Doi: 10.1016/j.cortex.2019.04.023.

Sol, D., Duncan, R.P., Blackburn, T.M., Cassey, P., Lefebvre, L. (2005). Big brains, enhanced cognition, and response of birds to novel environments. *PNAS, 105*(15), 5460-5465. Doi: 10.1073/pnas.0408145102.

Stankowich, T., Haverkamp, P.J., Caro, T. (2014). Ecological drivers of antipredator defences in carnivores. *Evolution, 68*(5), 1415-1425. Doi: 10.1111/evo.12356.

Street, S.E., Navarrete, A.F., Reader, S.M., Laland, K.N. (2017). Coevolution of cultural intelligence, extended life history, sociality, and brain size in primates. *PNAS, 114*(30), 7908-7914. Doi: 10.1073/pnas.1620734114.

Swanson, E.M., Holekamp, K.E., Lundrigan, B.L., Arsznov, B.M., Sakai, S.T. (2012). Multiple determinants of whole and regional brain volume among terrestrial carnivorans. *PLoS ONE, 7*(6), e38447. Doi: 10.1371/journal.pone.0038447.

Triki, Z., Levorato, E., McNeely, W., Marshall, J., Bshary, R. (2019). Population densities predict forebrain size variation in the cleaner fish Labroides dimidiatus. *Proceedings of the Royal Society B: Biological Sciences, 286*(1915), 20192108.

Uomini, N., Fairlie, J. Gray, R.D., Griesser, M. (2020). Extended parenting and the evolution of cognition. *Philosophical Transactions of the Royal Society B: Biological Sciences, 375*(1803), 20190495. Doi: 10.1098/rstb.2019.0495.

Upham, N.S., Esselstyn, J.A., Jetz, W. (2019). Inferring the mammal tree: species-level sets of phylogenies for questions in ecology, evolution, and conservation. *PLoS Biology, 17*(12), e3000494. Doi: 10.1371/journal.pbio.3000494.

van Schaik, C.P., Triki, Z., Bshary, R., Heldstab, S.A. (2021). A farewell to EQ: A new brain size measure for comparative primate cognition. *bioRxiv*. Doi: 10.1101/2021.02.15.431238.

van Schaik, C.P., Deaner, R.O. (2003). Life history and cognitive evolution in primates. In F. de Waal & P.L. Tyack (Eds.), *Animal Social Complexity: Intelligence, Culture, and Individualized Societies* (pp. 5-25). Cambridge: Harvard University Press.

van Schaik, C.P., Graber, S.M., Schuppli, C., Heldstab, S.A., Isler, K. (2016). Brain size evolution in primates - testing effects of social vs. ecological complexity. *American Journal of Physical Anthropology, 159*, 321-321.

van Schaik, C.P., Isler, K., Burkart, J.M. (2012). Explaining brain size variation: from social to cultural brain. *Trends in Cognitive Sciences, 16*(5), 277-284. Doi: 10.1016/j.tics.2012.04.004.

van Woerden, J.T., van Schaik, C.P., Isler, K. (2010). Effects of seasonality on brain size evolution: evidence from strepsirrhine primates. *The American Naturalist, 176*(6), 758-767. Doi: 10.1086/657045.

Walker, R., Burger, O., Wagner, J., Von Rueden, C.R. (2006). Evolution of brain size and juvenile periods in primates. *Journal of Human Evolution, 51*(5), 480-489. Doi: 10.1016/j.jhevol.2006.06.002.

Weisbecker, V., Goswami, A. (2010). Brain size, life history, and metabolism at the marsupial/placental dichotomy. *PNAS, 107*(37), 16216-16221. Doi: 10.1073/pnas.0906486107.

Wilman, H., Belmarker, J., Simpson, J., de la Rosa, C., Rivadeneira, M.M., Jetz, W. (2014). EltonTraits 1.0: species-level foraging attributes of the world's birds and mammals. *Ecology, 95*(7), 2027. Doi: 10.1890/13-1917.1.

1. Barton RA. Embodied cognitive evolution and the cerebellum. Philos Trans R Soc B. 2012;367(1599):2097-107.

2. Parker ST, Gibson KR. Object manipulation, tool use and sensorimotor intelligence as feeding adaptations in cebus monkeys and great apes. J Hum Evol. 1977;6(7):623-41.

Formatted: Font: 12 pt

Formatted: Font: (Default) +Body (Calibri), 12 pt

Formatted: Line spacing: Double

3. Milton K. Distribution patterns of tropical plant foods as an evolutionary stimulus to primate mental development. Am Anthropol. 1981;83(3):534-48.

4. Mace GM, Harvey PH, Clutton-Brock TH. Brain size and ecology in small mammals. J Zool. 1981;193(3):333-54.

5. Barton RA, Purvis A, Harvey PH. Evolutionary radiation of visual and olfactory brain systems in primates, bats and insectivores. Philos Trans R Soc B. 1995;348(1326):381-92.

6. Hutcheon JM, Kirsch JAW, Garland Jr T. A comparative analysis of brain size in relation to foraging ecology and phylogeny in the chiroptera. Brain Behav Evol. 2002;60(3):165-80.

7. Winkler H, Leisler B, Bernroider G. Ecological constraints on the evolution of avian brains. J Ornithol. 2004;145(3):238-44.

8. Harvey PH, Clutton-Brock TH, Mace GM. Brain size and ecology in small mammals and primates. PNAS. 1980;77(7):4387-9.

9. Clutton-Brock TH, Harvey PH. Primates, brains and ecology. J Zool. 1980;190(3):309-23.

10. Bernard RTF, Nurton J. Ecological correlates of relative brain size in some south african rodents. S Afr J Zool. 1993;28(2):95-8.

11. Barton RA. Primate brain evolution: cognitive demands of foraging or of social life? In: Boinski S, Garber PA, editors. On the move: how and why animals travel in groups. London: The University of Chicago Press; 2000. p. 204-37.

12. Heldstab SA, Kosonen ZK, Koski SE, Burkart JM, van Schaik CP, Isler K. Manipulation complexity in primates coevolved with brain size and terrestriality. Sci Rep. 2016;6:24528.

13. Parker ST. Re-evaluating the extractive foraging hypothesis. New Ideas Psychol. 2015;37:1-12.

14. Powell LE, Isler K, Barton RA. Re-evaluating the link between brain size and behavioural ecology in primates. Proc R Soc B. 2017;284(1865):20171765.

15. Milton K, May ML. Body weight, diet and home range area in primates. Nature. 1976;259(5543):459-62.

16. Walker R, Burger O, Wagner J, Von Rueden CR. Evolution of brain size and juvenile periods in primates. J Hum Evol. 2006;51(5):480-9.

17. Ratcliffe JM. Neuroecology and diet selection in phyllostomid bats. Behav Process. 2009;80(3):247-51.

18. van Woerden JT, van Schaik CP, Isler K. Effects of seasonality on brain size evolution: evidence from strepsirrhine primates. Am Nat. 2010;176(6):758-67.

19. Swanson EM, Holekamp KE, Lundrigan BL, Arsznov BM, Sakai ST. Multiple determinants of whole and regional brain volume among terrestrial carnivorans. PLoS One. 2012;7(6):e38447.

20. DeCasien AR, Williams SA, Higham JP. Primate brain size is predicted by diet but not sociality. Nat Ecol Evol. 2017;1(5):0112.

21. Gibson KR. Cognition, brain size and the extraction of embedded food resources. In: Lee PC, Else JG, editors. Primate ontogeny, cognition and social behaviour. Cambridge: Cambridge University Press; 1986. p. 93-103.

22. Reader SM, Hager Y, Laland KN. The evolution of primate general and cultural intelligence. Philos Trans R Soc B. 2011;366(1567):1017-27.

23. Plante S, Colchero F, Calmé S. Foraging strategy of a neotropical primate: how intrinsic and extrinsic factors influence destination and residence time. J Anim Ecol. 2014;83(1):116-25.

24. Sol D, Duncan R, Blackburn T, Cassey P, Lefebvre L. Big brains, enhanced cognition, and response of birds to novel environments. PNAS. 2005;102:5460-5.

25. Whiten A, Byrne RW. Tactical deception in primates. Behav Brain Sci. 1988;11(2):233-73.

26. Dunbar RIM. The social brain hypothesis. Evol Anthropol. 1998;6(5):178-90.

27. Dunbar RIM. The social brain hypothesis and its implications for social evolution. Ann Hum Biol. 2009;36(5):562-72.

28. Dunbar RIM. Neocortex size as a constraint on group size in primates. J Hum Evol. 1992;22(6):469-93.

29. Byrne RW, Corp N. Neocortex size predicts deception rate in primates. Proc R Soc B. 2004;271(1549):1693-9.

30. Kudo H, Dunbar RIM. Neocortex size and social network size in primates. Anim Behav. 2001;62(4):711-22.

31. Holekamp KE, Dantzer B, Stricker G, Shaw Yoshida KC, Benson-Amram S. Brains, brawn and sociality: a hyaena's tale. Anim Behav. 2015;103:237-48.

32. Sakai ST, Arsznov BM, Lundrigan BL, Holekamp KE. Brain size and social complexity: a computed tomography study in hyaenidae. Brain Behav Evol. 2011;77(2):91-104.

33. Dunbar RIM, Bever J. Neocortex size predicts group size in carnivores and some insectivores. Ethology. 1998;104(8):695-708.

34. Shultz S, Dunbar RIM. The evolution of the social brain: anthropoid primates contrast with other vertebrates. Proc R Soc B. 2007;274(1624):2429-36.

35. Pérez-Barbería FJ, Shultz S, Dunbar RIM. Evidence for coevolution of sociality and relative brain size in three orders of mammals. Evolution. 2007;61(12):2811-21.

36. Perez-Barberia FJ, Gordon IJ. Gregariousness increases brain size in ungulates. Oecologia. 2005;145(1):41-52.

37. Shultz S, Dunbar RIM. Both social and ecological factors predict ungulate brain size. Proc R Soc B. 2006;273(1583):207-15.

38. Emery N, Seed A, von Bayern A, Clayton N. Cognitive adaptations of social bonding in birds. Philos Trans R Soc B. 2007;362:489-505.

39. Scheiber IB, Weiß BM, Hirschenhauser K, Wascher CA, Nedelcu IT, Kotrschal K. Does 'relationship intelligence' make big brains in birds? Open Biol. 2008;1:6-8.

40. Shultz S, Dunbar RIM. Social bonds in birds are associated with brain size and contingent on the correlated evolution of life-history and increased parental investment. Biol J Linn Soc. 2010;100(1):111-23.

41. Gonzalez-Voyer A, Winberg S, Kolm N. Social fishes and single mothers: brain evolution in african cichlids. Proc R Soc B. 2009;276(1654):161-7.

42. Bshary R. Machiavellian intelligence in fishes. In: Brown C, Laland KN, Krause J, editors. Fish cognition and behavior. Chichester: Blackwell Publishing Ltd; 2011.

43. Triki Z, Levorato E, McNeely W, Marshall J, Bshary R. Population densities predict forebrain size variation in the cleaner fish Labroides dimidiatus. Proc R Soc B. 2019;286(1915):20192108.

44. Byrne RW, Bates Lucy A. Brain evolution: when is a group not a group? Curr Biol. 2007;17(20):R883-R4.

45. Bergman TJ, Beehner JC. Measuring social complexity. Anim Behav. 2015;103:203-9.

46. Schillaci MA. Sexual selection and the evolution of brain size in primates. PLoS One. 2006;1(1):e62.

47. Schillaci MA. Primate mating systems and the evolution of neocortex size. J Mammal. 2008;89(1):58-63.

48. MacLean EL, Barrickman NL, Johnson EM, Wall CE. Sociality, ecology, and relative brain size in lemurs. J Hum Evol. 2009;56(5):471-8.

49. Silk JB. The evolution of primate societies. In: John CM, Josep C, Peter MK, Ryne AP, Joan BS, editors. The adaptive value of sociality: University of Chicago Press; 2012. p. 552-64.

50. MacLean EL, Hare B, Nunn CL, Addessi E, Amici F, Anderson RC, et al. The evolution of self-control. PNAS. 2014;111(20):E2140-8.

51. van Schaik C, Graber SM, Schuppli C, Heldstab SA, Isler K. Brain size evolution in primates-testing effects of social vs. ecological complexity. Am J Phys Anthropol. 2016;159:321-.

52. Holekamp KE, Benson-Amram S. The evolution of intelligence in mammalian carnivores. Interface Focus. 2017;7(3):20160108.

53. Holekamp KE. Questioning the social intelligence hypothesis. Trends Cogn Sci. 2007;11(2):65-9.

54. van Schaik CP, Isler K, Burkart JM. Explaining brain size variation: from social to cultural brain. Trends Cogn Sci. 2012;16(5):277-84.

55. Finarelli JA, Flynn JJ. Brain-size evolution and sociality in carnivora. PNAS. 2009;106(23):9345-9.

56. Jerison HJ. Evolution of the brain and intelligence. New York: Academic Press; 1973.

57. Deaner RO, Isler K, Burkart J, van Schaik C. Overall brain size, and not encephalization quotient, best predicts cognitive ability across non-human primates. Brain Behav Evol. 2007;70(2):115-24.

58. Shultz S, Dunbar R. Encephalization is not a universal macroevolutionary phenomenon in mammals but is associated with sociality. PNAS. 2010;107(50):21582-6.

59. Freckleton RP. On the misuse of residuals in ecology: regression of residuals vs. multiple regression. J Anim Ecol. 2002;71(3):542-5.

60. Freckleton RP. The seven deadly sins of comparative analysis. J Evol Biol. 2009;22(7):1367-75.

61. Barton RA, Harvey PH. Mosaic evolution of brain structure in mammals. Nature. 2000;405(6790):1055-8.

62. Deaner RO, Nunn CL. How quickly do brains catch up with bodies? A comparative method for detecting evolutionary lag. Proc R Soc B. 1999;266(1420):687-94.

63. Smaers JB, Dechmann DKN, Goswami A, Soligo C, Safi K. Comparative analyses of evolutionary rates reveal different pathways to encephalization in bats, carnivorans, and primates. PNAS. 2012;109(44):18006-11.

64. Smaers JB, Rothman RS, Hudson DR, Balanoff AM, Beatty B, Dechmann DKN, et al. The evolution of mammalian brain size. Sci Adv. 2021;7(18):eabe2101.

65. van Schaik CP, Triki Z, Bshary R, Heldstab SA. A farewell to EQ: A new brain size measure for comparative primate cognition. bioRxiv. 2021:2021.02.15.431238.

66. Finlay B, Darlington RB. Linked regularities in the development and evolution of mammalian brains. Science. 1995;268:1578-84.

67. Finlay BL, Darlington RB, Nicastro N. Developmental structure in brain evolution. Behav Brain Sci. 2001;24(2):263-78.

68. Cantania K. Correlates and possible mechanisms of neocortical enlargement and diversification in mammals. Int J Comp Psychol. 2004;17(1).

69. Innocenti GM, Kaas JH. The cortex. Trends Neurosci. 1995;18(9):371-2.

70. Kaas JH. The evolution of isocortex. Brain Behav Evol. 1995;46(4-5):187-96.

71. Barton RA. Visual specialization and brain evolution in primates. Proc R Soc B. 1998;265(1409):1933-7.

72. Jacobs GH. The distribution and nature of colour vision among the mammals. Biol Rev. 1993;68(3):413-71.

73. Jacobs GH. Variations in primate color vision: mechanisms and utility. Evol Anthropol. 1994;3(6):196-205.

74. Jacobs GH. Primate photopigments and primate color vision. PNAS. 1996;93(2):577-81.

75. Sussman RW. Primate origins and the evolution of angiosperms. Am J Primatol. 1991;23(4):209-23.

76. Brothers L. The neural basis of primate social communication. Motiv Emot. 1990;14(2):81-91.

77. Barton RA. Neocortex size and behavioural ecology in primates. Proc R Soc B. 1996;263(1367):173-7.

78. Barton RA. Binocularity and brain evolution in primates. PNAS. 2004;101(27):10113-5.

79. Barton RA. Olfactory evolution and behavioral ecology in primates. Am J Primatol. 2006;68(6):545-58.

80. Whiting BA, Barton RA. The evolution of the cortico-cerebellar complex in primates: anatomical connections predict patterns of correlated evolution. J Hum Evol. 2003;44(1):3-10.

81. Butler AB, Hodos W. Comparative vertebrate neuroanatomy: evolution and adaptation. 2nd ed. New Jersey: John Wiley & Sons Inc; 2005.

82. Iwaniuk AN, Lefebvre L, Wylie DR. The comparative approach and brain-behaviour relationships: a tool for understanding tool use. Can J Exp Psychol. 2009;63(2):150-9.

83. Obayashi S, Suhara T, Kawabe K, Okauchi T, Maeda J, Akine Y, et al. Functional brain mapping of monkey tool use. NeuroImage. 2001;14(4):853-61.

84. Barton Robert A, Venditti C. Rapid evolution of the cerebellum in humans and other great apes. Curr Biol. 2014;24(20):2440-4.

85. Wolpert DM, Doya K, Kawato M. A unifying computational framework for motor control and social interaction. Philos Trans R Soc B. 2003;358(1431):593-602.

86. Oztop E, Wolpert D, Kawato M. Mental state inference using visual control parameters. Cogn Brain Res. 2005;22(2):129-51.

87. Smaers JB, Vanier DR. Brain size expansion in primates and humans is explained by a selective modular expansion of the cortico-cerebellar system. Cortex. 2019;118:292-305.

88. Smaers JB, Turner AH, Gómez-Robles A, Sherwood CC. A cerebellar substrate for cognition evolved multiple times independently in mammals. eLife. 2018;7:e35696.

89. Fernandes HBF, Peñaherrera-Aguirre M, Woodley of Menie MA, Figueredo AJ. Macroevolutionary patterns and selection modes for general intelligence (G) and for commonly used neuroanatomical volume measures in primates. Intelligence. 2020;80:101456.

90. Isler K, van Schaik CP. Allomaternal care, life history and brain size evolution in mammals. J Hum Evol. 2012;63(1):52-63.

91. DeCasien AR, Higham JP. Primate mosaic brain evolution reflects selection on sensory and cognitive specialization. Nat Ecol Evol. 2019;3(10):1483-93.

92. Sakai ST, Arsznov BM, Hristova AE, Yoon EJ, Lundrigan BL. Big cat coalitions: a comparative analysis of regional brain volumes in felidae. Front Neuroanat. 2016;10:99.

93. Heldstab S, Isler K. Environmental seasonality and mammalian brain size evolution: Wiley Online Library; 2019.

94. Dunbar RIM, Shultz S. Understanding primate brain evolution. Philos Trans R Soc B. 2007;362(1480):649-58.

95. Dunbar RIM, Shultz S. Why are there so many explanations for primate brain evolution? Philos Trans R Soc B. 2017;372(1727):20160244.

96. Isler K, Christopher Kirk E, Miller JM, Albrecht GA, Gelvin BR, Martin RD. Endocranial volumes of primate species: scaling analyses using a comprehensive and reliable data set. J Hum Evol. 2008;55(6):967-78.

97. Stephan H, Frahm H, Baron G. New and revised data on volumes of brain structures in insectivores and primates. Folia Primatol. 1981;35(1):1-29.

98. Sallet J, Mars RB, Noonan MP, Andersson JL, O'Reilly JX, Jbabdi S, et al. Social network size affects neural circuits in macaques. Science. 2011;334(6056):697-700.

99. Powell J, Lewis PA, Roberts N, García-Fiñana M, Dunbar RIM. Orbital prefrontal cortex volume predicts social network size: an imaging study of individual differences in humans. Proc R Soc B. 2012;279(1736):2157-62.

100. Dunbar RIM, Shultz S. Evolution in the social brain. Science. 2007;317(5843):1344-7.

101. Stankowich T, Haverkamp PJ, Caro T. Ecological drivers of antipredator defenses in carnivores. Evolution. 2014;68(5):1415-25.

102. Wilman H, Belmaker J, Simpson J, de la Rosa C, Rivadeneira MM, Jetz W. EltonTraits 1.0: Species-level foraging attributes of the world's birds and mammals. Ecology. 2014;95(7):2027-.

103. IUCN. The IUCN red list of threatened species [Internet]. United Kingdom: International Union for Conservation of Nature and Natural Resources; 2021 [updated 2021, cited Jul 2, 2020]. Available from: https://www.iucnredlist.org/.

104. Deaner RO, Barton RA, van Schaik C. Primate brains and life histories: renewing the connection. In: Kappeler PM, Pereira ME, editors. Primate life histories and socioecology. Chicago: The University of Chicago Press; 2003. p. 233-65.

105. Powell LE, Barton RA, Street SE. Maternal investment, life histories and the evolution of brain structure in primates. Proc R Soc B. 2019;286(1911):20191608.

106. Isler K, Van Schaik CP. How humans evolved large brains: comparative evidence. Evol Anthropol. 2014;23(2):65-75.

107. Weisbecker V, Goswami A. Brain size, life history, and metabolism at the marsupial/placental dichotomy. PNAS. 2010;107(37):16216-21.

108. DeCasien AR, Thompson NA, Williams SA, Shattuck MR. Encephalization and longevity evolved in a correlated fashion in euarchontoglires but not in other mammals. Evolution. 2018;72(12):2617-31.

109. Street SE, Navarrete AF, Reader SM, Laland KN. Coevolution of cultural intelligence, extended life history, sociality, and brain size in primates. PNAS. 2017;114(30):7908-14.

110. Barton RA, Capellini I. Maternal investment, life histories, and the costs of brain growth in mammals. PNAS. 2011;108(15):6169-74.

111. Barrickman NL, Bastian ML, Isler K, van Schaik CP. Life history costs and benefits of encephalization: a comparative test using data from long-term studies of primates in the wild. J Hum Evol. 2008;54(5):568-90.

112. Symonds MRE, Blomberg SP. A primer on phylogenetic generalised least squares. In: Garamszegi LZ, editor. Modern phylogenetic comparative methods and their application in

evolutionary biology: concepts and practice. Berlin, Heidelberg: Springer Berlin Heidelberg; 2014. p. 105-30.

113. Upham NS, Esselstyn JA, Jetz W. Inferring the mammal tree: species-level sets of phylogenies for questions in ecology, evolution, and conservation. PLoS Biol. 2019;17(12):e3000494.

114. Schwarz G. Estimating the dimension of a model. Ann Stat. 1978;6(2):461-4, 4.

115. Aiello LC, Wheeler P. The expensive-tissue hypothesis: the brain and the digestive system in human and primate evolution. Curr Anthropol. 1995;36(2):199-221.

116. Fish JL, Lockwood CA. Dietary constraints on encephalization in primates. Am J Phys Anthropol. 2003;120(2):171-81.

117. Graber SM. Social and ecological aspects of brain size evolution: a comparative approach [PhD Thesis]. Zurich: University of Zurich; 2017.

118. Layton R, O'Hara S. Human social evolution: a comparison of hunter gather and chimpanzee social organization. In: Dunbar R, Gamble C, Gowlett J, editors. Social brain, distributed mind. Oxford: British Academy; 2010. p. 85-115.

119. Martin RD. Scaling of the mammalian brain: the maternal energy hypothesis. Physiology. 1996;11(4):149-56.

120. Heldstab SA, Isler K, Burkart JM, van Schaik CP. Allomaternal care, brains and fertility in mammals: who cares matters. Behav Ecol Sociobio. 2019;73(6):71.

121. Isler K, van Schaik CP. The expensive brain: a framework for explaining evolutionary changes in brain size. J Hum Evol. 2009;57(4):392-400.

122. Heldstab SA, Isler K, Schuppli C, van Schaik CP. When ontogeny recapitulates phylogeny: Fixed neurodevelopmental sequence of manipulative skills among primates. Sci Adv. 2020;6(30):eabb4685.

123. Uomini N, Fairlie J, Gray RD, Griesser M. Extended parenting and the evolution of cognition. Philos Trans R Soc B. 2020;375(1803):20190495.

124. González-Lagos C, Sol D, Reader S. Large-brained mammals live longer. J Evol Biol. 2010;23:1064-74.

125. Leggio MG, Chiricozzi FR, Clausi S, Tedesco AM, Molinari M. The neuropsychological profile of cerebellar damage: the sequencing hypothesis. Cortex. 2011;47(1):137-44.

126. Rochefort C, Arabo A, André M, Poucet B, Save E, Rondi-Reig L. Cerebellum shapes hippocampal spatial code. Science. 2011;334(6054):385-9.

127. Benson-Amram S, Dantzer B, Stricker G, Swanson EM, Holekamp KE. Brain size predicts problem-solving ability in mammalian carnivores. PNAS. 2016;113(9):2532-7.

128. Sakai ST, Arsznov BM. Carnivoran brains: effects of sociality on inter- and intraspecific comparisons of regional brain volumes. In: Kaas JH, editor. Evolutionary neuroscience. 2nd ed. London: Academic Press; 2020. p. 463-79.

129. Healy SD, Rowe C. A critique of comparative studies of brain size. Proc R Soc B. 2007;274(1609):453-64.

Reviewer #1

1) I am not sure why this manuscript deals with just primates and carnivores. Why these two orders of mammals? Why not other orders such as rodents, lagomorphs, shrews, and bats? In fact, there are plenty of extensive datasets for these (and other) orders. For example, see Mace et al (1981) J. Zool. 193:333-354, which presents brain size data for 261 species of terrestrial small mammals, and Hutcheon et al. (2002) Brain Behavior Evolution 60:165-180 for 63 species of bats. I would have thought that a comparative approach across the entire class Mammalia would have been more fruitful than simply presenting data on primates and (incongruously) carnivores. The authors make no attempt to justify their selection of mammalian orders.

Whilst we understand that brain data are available for more species than which were included within the manuscript, we wanted to run analyses on a complete dataset with all covariates available for all species, as this enabled more robust analyses, especially when conducting model comparisons. We could access all the required covariates for primates and carnivores, which governed our choice. In addition, in efforts to address the current confusion within the field regarding the proposed selection pressures responsible for increased brain size, we chose to use both primate and carnivore data as these two groups have received considerable attention, and thus by drawing clarity within these two groups, further groups can be studied using more appropriate methods/procedures. **We have added wording to emphasise our reasoning for this choice.**

2) The literature cited is not representative of the field. A good deal of previous work has been omitted from this ms, including the two papers mentioned in (1) above, as well as Harvey et al. (1980) PNAS 77:4387-4389 (this paper explicitly deals with primate brain sizes). And there are many more papers that deal with ecological correlates of brain sizes that have not been mentioned.

Additional citations have been added.

3) Although the manuscript is generally well written, there are some sections that are difficult to interpret and/or to follow. This is particularly true for the Methods section, which is often ambiguous or at least incomplete. See below for where more detail is needed.

Wording has been rephrased for added clarity.

4) There is no definition of what is meant by the different brain volumes that are presented in the ms. For example, how was "endocranial brain volume" measured? And was it measured in the same way in the different papers where this information was extracted and collated? If not, then how can we be sure that we are comparing like with like?

5) The same comment applies to "neocortex" and "cerebellum" volumes.

Definitions have been added for endocranial and regional brain volumes. When sourcing all whole and regional brain volumes these measurement methods were considered to ensure the data was comparable. In terms of the ECV data, sources were checked for comparability and common measurement techniques were found between studies. We further tried to minimise the risk of this problem by sourcing data from whole datasets e.g., DeCasien et al., 2019 where the information has been weighted to account for multiple methods. However, this was more difficult with the carnivore data where regional brain volume data was tricky to source.

6) Again, how was social cohesion measured? I can see that it was scored on a point system of 1 to 4, but what does it mean for a species to have a social cohesion of 1? or 2? etc.

Definition revised for greater clarity.

7) I found the ecological data simplistic and not at all credible. The authors will need to justify exactly what they mean by each of the ecological variables. And then, they will need to convince the reader that the ecological data are actually meaningful. I am happy to include "diet" (although "frugivore" or "omnivore" are diet categories rather than strictly speaking diet itself (and the authors actually refer to diet categories, but they don't explicitly make the distinction). But what do they mean by diet breadth? According to their definition it is: "dietary breadth was also used, estimated using the total number of food sources used by a species". But what are these "food sources"? Are they the number of species of plants/animals taken? If so, an insectivorous species will by definition have a wider breadth than a carnivorous one (because there are more species of insects than vertebrates). If "sources" refers to something else, then what is it? And then, once the definition has been clearly stated, how can we be sure that the different studies have scored "number of food sources" in the same way?

Definitions of dietary categories and dietary breadth revised for greater clarity. All dietary breadth data was taken from one source: Wilman et al., (2014) and is referred to in the manuscript.

8) I have even more issue with the number of habitats used by a species. Wider ranging species will use a greater number of habitats, so why didn't the authors correct for this? Or simply use distributional range size instead of number of habitats?

Whilst we understand and appreciate this point, it does not always follow that wider ranging species will always use a greater number of habitats. One species may have a large home range size but may only move within the same habitat type. What we instead aim to look at here is whether the type of habitat matters, thus, do species which navigate and confront multiple habitat types, have larger brains than those which only move within one or two habitat types? Or vice versa? We also use home range size to proxy habitat use.

9) The authors do not mention where they get their home range sizes from in the ms (although these are clearly mentioned in the supplementary material). I find it hard to believe that the various range sizes compiled by numerous authors will be directly comparable due to differences in techniques used to estimate home range. Furthermore, there is enormous amount of variation in home range size, which is partly (and only partly) attributable to sex and age. Using a single metric is hardly informative or convincing.

We did not want to mention the citations specifically within the manuscript due to the high number of citations. We agree with this point about transferability of the methods used to measure home range size. We did our best to reduce the number of sources due to this problem, however, due to limited data availability, the only way to retrieve home range size for all species was to use data from multiple studies. To minimise the issue highlighted, we chose to use hectares to measure home range size as this was the most prevalent method found. We converted all home range data collected to this metric. We agree a single metric is not always useful, which is why we used both habitat variability and home range size to proxy habitat use.

10) Statistical analysis. This entire section (lines 218 to 239) needs to be reworked and more detail provided. And unambiguous statements rephrased. I will make just a few examples (but these are not the only problems).

Wording has been rephrased for clarity.

11) Lines 219-220 "using residuals from a regression line". Regression of what on what? And exactly using what regression? Simple linear regression e.g. lm()? On log transformed or untransformed data?

Phrase removed as this aspect has been moved to supplementary methods. This regression analysis is discussed in full within that document… “*Phylogenetic generalised least-squares regression analysis (PGLS) was used to regress log brain volume against log body mass*”.

12) What is the encephalisation quotient and how was it calculated? In fact, the equation is presented a bit further down, so perhaps the authors just need to refer to this e.g. say something like "see below for equation".

Definition revised for greater clarity. This aspect – as mentioned above – has been moved to the supplementary methods.

13) Line 220. "The former method is often preferred...". But you can't use "former" when there are three methods presented. "Former" and "latter" can only be used when comparing two things.

Thank you for highlighting. Phrase removed.

14) Line 226. "...therefore we considered it prudent to use both methods in the analyses...". Which two methods are being referred to? Because the authors have mentioned three methods (which have even been numbered).

Phrase removed.

15) Please provide a basic description of "Phylogenetic generalised least-squares regression analysis" and how it differs from typical GLMs.

Definition revised to provide greater clarity.

16) VIF was used to check for collinearity (which is good). But what does it mean "almost all scores" were below 5. Which variables were autocorrelated? And were any removed from the analyses, as a result of this?

Almost all VIF scores produced were below 5, however there were a few outliers. For example, body mass and weaning age produced scores of 7.25 and 5.93, when inputted into the primate endocranial model. Whilst moderately high, we chose to retain all variables within the statistical models, as the scores were only found in a few models and were still considerably low. Thus, no variables were removed from the analyses. VIF scores were also checked when rerunning analyses, specifically when using the 'rest of brain' regional volume technique, with no scores produced of concern.

This sentence has been updated to provide greater clarity.

17) Possible limitations. I find this paragraph difficult to accept. The authors are well aware that any models with AICs within 2 points are not "statistically different". Then how can they justify their approach? To me, this is the weakest aspect of the ms, because it affects all of their interpretations. There must be better ways of dealing with this. For example, list all competing models, and then count the number of times a particular variable (e.g. social cohesion) appears in the top models? This may make the results much more difficult to interpret, but this may be because there really is no simple and easy answer to the question that they are asking. Simplifying a complex problem with incorrect statistics is not acceptable.

We appreciate this comment. We agree this was a weak point in the analyses. To address this highlighted shortcoming, rather than just choosing the model with the absolute lowest score, we have now adopted the approach of presenting and discussing the results of all the 'best fit' models, which usually included a subset of models (simply, all the models within 2 points of the absolute lowest model). We have also rerun the analysis using BIC rather than AIC, in acknowledgement of this scoring system being more conservative.

Reviewer #2

- Line 33: See my comment in the Discussion section on the use of "counterbalancing".

Wording rephrased.

- There is a critical part currently missing this section, which is an explicit discussion of how this study is different from the many previous analyses of brain ~ socioecology relationships (e.g., inclusion of more variables, updated phylogeny, higher individual/species sample sizes)?

Thank you for this comment, we agree this was lacking in the manuscript. **Introduction has been updated with this discussion.**

- Line 75: The importance of pair-bondedness to brain size evolution was also discussed in other papers, which should be cited here (Schillaci 2006, 2008; MacLean et al. 2009).
- Line 83: This reference is only for carnivores – please add a reference for primates.

Citations added.

- Paragraph starting with Line 90:
  - I think a discussion of issues with relative brain size measures is important, however, I don't think it warrants using measures that have been previously established as inappropriate (i.e., residuals, EQ).
- Lines 141-144: Again, it is unnecessary to include analyses using EQ or brain size residuals.
- Lines 218-220: Again, it is unnecessary to include analyses using EQ or brain size residuals.
- Paragraph starting with Line 467: As previously mentioned, previous studies have demonstrated that the use of EQ or residuals is inappropriate, so I think this paragraph and the relevant results are unnecessary and make the overall findings harder to follow.

We appreciate that these methods have previously been suggested to be inappropriate for measuring the relationship between brain size and body mass. We feel it is necessary to further address this problem, however, especially considering we are using updated data, updated statistical analysis, more variables and an updated phylogenetic tree. **After considering this point, we decided to move the results produced using the methods of concern (i.e., residuals, EQ) to the supplementary material and these will no longer be discussed in the main manuscript.** This moves the focus away from those methods, but still allows the comparison between methods which may be useful to some readers.

  - The findings from the most recent study on brain ~ body size evolution (Smears et al. 2021) should be considered/discussed here.
  - Freckleton's (2009) "seven deadly sins of comparative analysis" should be mentioned here, as it includes a discussion on why it is inappropriate to use residuals as outcome variables in regression models.
  - Lines 105-107 – Papers on lag between primate brain and body size should be mentioned here (e.g., Deaner and Nunn 1999).

Thank you. Citations added.

  - Line 108: It is unclear what "over statistically controlled methods" means here.

Wording rephrased.

  - Line 109: How and why does van Schaik et al. (2021) specifically demonstrate that EQ is inappropriate? The authors should elaborate a bit here.

Some elaboration has been added, as recommended.

- Paragraph starting with Line 111:
    - How would social and ecological variables specifically relate to neocortical and cerebellar functions?
- Increased brain size is the result of selection on specific abilities and related neural systems. Accordingly, at some point in this Introduction, I would appreciate a brief but explicit discussion of this (e.g., why might frugivory require greater visual information processing? Given that a large proportion of the brain is neocortex, and a large proportion of the neocortex is comprised of visual information processing areas, might this explain the link between something like frugivory and overall brain size?)

These points are now discussed.

- I think it would be appropriate to discuss Powell et al. (2019) here (currently only mentioned in the Discussion).

Powell et al., (2019) has been discussed further in the methods section.

- Line 126: What kind of "models"?

Sentence has been elaborated upon.

- Line 155: Please add sample sizes for the neocortex and cerebellum.

Sample sizes updated.

- Lines 157-161: This is Introduction material and should be removed from the Methods.
- Paragraph starting with Line 163: It might be useful to include some of this in the Introduction, since readers have any background surrounding issues with various "social complexity" measures.
- All descriptions of the links between socioecological variables and selection for cognitive abilities would be more appropriate in the Introduction.

These sections have been moved to the introduction.

- Lines 171-174: What were levels 2 and 3? How were pairbonded species or those that only sleep in pairs categorized? These levels need more explanation, especially since this "social cohesion" proxy was included in many best fit models in the Results.

Agreed. Definition revised for greater clarity.

- Lines 196-197: Diet imposes both temporal and spatial cognitive demands, so I suggest re-wording this.
- Lines 200-203: The authors appear to be suggesting that certain life history variables are drivers of evolutionary changes in brain size. I suggest altering the language here to mimic that in Lines 421-424.

Sentences rephrased for clarity.

- Paragraph staring with Line 200: This section is missing a discussion of ideas that the relationship between brain size and lifespan is driven by maternal investment and between specific brain regions and developmental periods (see e.g., Barton et al. 2011; Powell et al. 2019)

This point has been discussed.

- Lines 238-239: Why was body mass used as the covariate for the neocortex and cerebellum models? Many other papers have used brain size (with the brain region of interest removed) or medulla size as a covariate. This decision should be justified in the text or analyses should be re-run using a brain size measure.

Thank you for this comment, we agree that this method needed to be altered. **Neocortex and cerebellum size were recalculated using endocranial volume minus the brain region of interest. Analyses were re-run using this brain size measure.** The method (brain transformations) section has been updated to reflect this change.

- Model comparisons section:
    - This section as written is unclear – were the best fit models within Models 1-4 first identified, and then combined to make Model 5?
    - In any case, I do not think this approach is appropriate since it may, in some cases, force the inclusion of low information variables into the "combined" model. It would be more appropriate to create models that include all combinations of all predictor variables, compare these models using information criterion (I suggest using BIC since it is more conservative), and then select the best fit model or subset of models (e.g., all models with dBIC<2) to present detailed results.

Models one to four contained all combinations of the predictor variables, specifically looking at 1) social, 2) ecological, 3) social & ecological and 4) life history. Then usually models 3 and 4 were combined to determine whether incorporating the models together produced a better information criterion score. I say usually because sometimes incorporating social variables did not improve the score, therefore models 2 and 4 were combined instead. This combined model was also compared against a model including all variables together. We chose to use this 'combined' model because it would take too much time to try every combination of the 11 variables, therefore we thought by combining best fit models, this would bypass this problem and produce superior models. We appreciate your comment about the inclusion of low information variables, and it is definitely something we considered. **After your suggestion, to better address the issue, the analyses have been re-run using BIC instead of AIC, due to the fact it is more conservative and would reduce the likelihood of low information variables being included. We also chose to present the results of the 'best fit' models, which was usually a subset of models (presenting all models within dBIC<2 of the absolute lowest model).**

- Lines 260-261: The meaning of "presently, and subsequently" is unclear.

Phrase removed for clarity.

- This section is a bit difficult to follow as written. I suggest, within each section, more clearing separating/identifying the different groups of results. I think it would be most appropriate to first discuss results using the information criterion (i.e., tell the readers which variables are

included in the best fit models) and then the frequentist results (i.e., tell the readers which coefficient estimates within the best fit model are "significant" and the direction of the relationship)?

Thank you for this comment, we agree and the results section has been rewritten to allow greater clarity.

- Table 2: The diet category results (DFrug, DOmni) only demonstrate differences between these dietary groups (frugivory and omnivory) and folivory. This needs to be explicitly stated in the relevant areas of the results section. In addition, models should be run with the levels switched so that potential differences between frugivory and omnivory can also be tested.

Thank you for this comment, we agree that this needed highlighting. This has now been explicitly stated in the primate results section. In addition, as suggested, models were run with the levels switched, to identify any potential differences between frugivory and omnivory. This was checked on all 'best fit' models where diet was included, thus, on both the primate neocortex and cerebellum combined models. To do this, primate regional volume data was used, with linear regression models implemented, using the same combination of variables seen in the combined models (Neo ~ D + HR + ML + WA, Cere ~ D + HR + ML + WA).

Just included for your information…
Looking at primate neocortex data, when folivory was used as the baseline, negative significant associations were found with both omnivory and frugivory. However, when frugivory was used as the baseline, a positive association was found with folivory, whereas a negative association was found with omnivory. When omnivory was used as the baseline, positive associations were found with both frugivory and folivory. Thus, folivores appear to have larger neocortex volumes when compared to those with frugivorous and omnivorous diets, and this statement holds when the levels are switched (frugivorous and omnivorous species have smaller neocortex volumes when compared to those with a folivorous diet). However, frugivores appear to have larger neocortex volumes when compared to omnivores, and again, this statement holds when the levels are switched (omnivorous species have smaller neocortex sizes when compared to frugivorous species).

Looking at primate cerebellum data, the results are similar; both folivorous and frugivorous species appear to have larger cerebellar volumes when compared to those with an omnivorous diet, with this statement holding when the levels are switched (omnivorous species have smaller cerebellum volumes when compared to those with folivorous and frugivorous diets). However, there appears to be no discernible difference between folivorous and frugivorous species in terms of cerebellum volume.

- Lines 287-288 and 303-304: Table 2 includes results from *best fit models only* – it would be appropriate to also mention Table 1.

Table 1 has also been mentioned.

- Lines 288-289: Diet is not included in the best fit model for ECV in Table 1, so I am a bit confused about the claim that diet is positively associated with all brain measures.

What we meant by this sentence was that diet as a whole (dietary categories or dietary breadth) was associated with all brain measures. We agree this should have been better worded. This sentence has been removed, however, following the recommendation to no longer discuss the different brain measures in the main manuscript.

- Paragraph starting in Line 345: The home range results for the neocortex are not mentioned.

Thank you for pointing this out. We have now ensured all results are now appropriately discussed.

- Lines 383-385: The finding that habitat variability is negatively correlated with relative brain size should be discussed in terms of previous work demonstrating a negative impact of seasonality on brain size (e.g., van Woerden et al. 2010).

This correlation is no longer found after rerunning statistical analyses so has been removed.

- Lines 409-410: This is not true. Powell et al. (2019) found correlations between specific brain regions (neocortex) and gestation length. Other regions were correlated with other developmental periods (e.g., cerebellum and juvenile period).

Sentence updated to reflect this point.

- Line 421: What does “counterbalance” mean? It sounds as if animals are actively participating in the evolution of these traits. Can the authors elaborate on how specific selection mechanisms would drive this “counterbalancing”?

Sentence updated to reflect this point.

- Lines 426-427: This sentence makes it seem that diet category is included in the best fit models for carnivores, which is not the case. I suggest removing the sentence.

Sentence removed as recommended.

- Lines 443-446: Sociality is not included in any of the best fit models of relative brain size, so this sentence is misleading as written.

Sentence changed following reanalysis of data.

- Lines 445-457: I would remove this sentence since the cerebellum is showing opposite trends across groups.

Sentence removed.
