# Supplementary material for: Why big brains? A comparison of models for both primate and carnivore brain size evolution
Source: PLoS One. 2021 Dec 21;16(12):e0261185. doi: 10.1371/journal.pone.0261185 (PMC8691615; doi:10.1371/journal.pone.0261185)
Supplement: Attachment — Submitted filename: Comments to the Author_PONE-D-21-12399.docx [file pone.0261185.s010.docx]

**Comments to the Author**

**Review of “Why big brains? A comparison of models for both primate and carnivore brain size evolution” (PONE-D-21-12399)**

This is an interesting manuscript that addresses a well-studied but still controversial topic – which socioecological and life history factors primarily drive species differences in relative brain size. The goal of this manuscript is to test which social, ecological, and life history factors best predict relative brain size in primates and carnivores, after controlling for body size and phylogeny. To accomplish this, they gather an impressive amount of data and perform phylogenetic regression analyses. Throughout, they describe their analyses in detail, utilize up-to-date data and methods, and provide useful background information about relevant studies. This manuscript has a lot of potential – I recommend major revisions, due to some missing critical literature sources, the limitations of the model comparisons approach used, the organization of the results section, and the inclusion of unnecessary/inappropriate outcome variables in some models.

Abstract

- Line 33: See my comment in the Discussion section on the use of “counterbalancing”.

Introduction

- There is a critical part currently missing this section, which is an explicit discussion of how this study is different from the many previous analyses of brain ~ socioecology relationships (e.g., inclusion of more variables, updated phylogeny, higher individual/species sample sizes)?
- Line 75: The importance of pair-bondedness to brain size evolution was also discussed in other papers, which should be cited here (Schillaci 2006, 2008; MacLean et al. 2009).
- Line 83: This reference is only for carnivores – please add a reference for primates.
- Paragraph starting with Line 90:
  - I think a discussion of issues with relative brain size measures is important, however, I don’t think it warrants using measures that have been previously established as inappropriate (i.e., residuals, EQ).
  - The findings from the most recent study on brain ~ body size evolution (Smears et al. 2021) should be considered/discussed here.
  - Freckleton’s (2009) “seven deadly sins of comparative analysis” should be mentioned here, as it includes a discussion on why it is inappropriate to use residuals as outcome variables in regression models.
  - Lines 105-107 – Papers on lag between primate brain and body size should be mentioned here (e.g., Deaner and Nunn 1999).
  - Line 108: It is unclear what “over statistically controlled methods” means here.
  - Line 109: How and why does van Schaik et al. (2021) specifically demonstrate that EQ is inappropriate? The authors should elaborate a bit here.
- Paragraph starting with Line 111:
  - How would social and ecological variables specifically relate to neocortical and cerebellar functions?
  - I think it would be appropriate to discuss Powell et al. (2019) here (currently only mentioned in the Discussion).
- Line 126: What kind of “models”?
- Lines 141-144: Again, it is unnecessary to include analyses using EQ or brain size residuals.
- Increased brain size is the result of selection on specific abilities and related neural systems. Accordingly, at some point in this Introduction, I would appreciate a brief but explicit discussion of this (e.g., why might frugivory require greater visual information processing? Given that a large proportion of the brain is neocortex, and a large proportion of the neocortex is comprised of visual information processing areas, might this explain the link between something like frugivory and overall brain size?)

Methods

- Line 155: Please add sample sizes for the neocortex and cerebellum.
- Lines 157-161: This is Introduction material and should be removed from the Methods.
- Paragraph starting with Line 163: It might be useful to include some of this in the Introduction, since readers have any background surrounding issues with various “social complexity” measures.
- Lines 171-174: What were levels 2 and 3? How were pairbonded species or those that only sleep in pairs categorized? These levels need more explanation, especially since this “social cohesion” proxy was included in many best fit models in the Results.
- All descriptions of the links between socioecological variables and selection for cognitive abilities would be more appropriate in the Introduction.
- Lines 196-197: Diet imposes both temporal and spatial cognitive demands, so I suggest re-wording this.
- Lines 200-203: The authors appear to be suggesting that certain life history variables are drivers of evolutionary changes in brain size. I suggest altering the language here to mimic that in Lines 421-424.
- Paragraph staring with Line 200: This section is missing a discussion of ideas that the relationship between brain size and lifespan is driven by maternal investment and between specific brain regions and developmental periods (see e.g., Barton et al. 2011; Powell et al. 2019)
- Lines 218-220: Again, it is unnecessary to include analyses using EQ or brain size residuals.
- Lines 238-239: Why was body mass used as the covariate for the neocortex and cerebellum models? Many other papers have used brain size (with the brain region of interest removed) or medulla size as a covariate. This decision should be justified in the text or analyses should be re-run using a brain size measure.
- Model comparisons section:
  - This section as written is unclear – were the best fit models within Models 1-4 first identified, and then combined to make Model 5?
  - In any case, I do not think this approach is appropriate since it may, in some cases, force the inclusion of low information variables into the “combined” model. It would be more appropriate to create models that include all combinations of all predictor variables, compare these models using information criterion (I suggest using BIC since it is more conservative), and then select the best fit model or subset of models (e.g., all models with dBIC<2) to present detailed results.
- Lines 260-261: The meaning of “presently, and subsequently” is unclear.

Results

- This section is a bit difficult to follow as written. I suggest, within each section, more clearing separating/identifying the different groups of results. I think it would be most appropriate to first discuss results using the information criterion (i.e., tell the readers which variables are included in the best fit models) and then the frequentist results (i.e., tell the readers which coefficient estimates within the best fit model are “significant” and the direction of the relationship)?
- Tables 1-4 are very clear and well organized.
- Table 2: The diet category results (DFrug, DOmni) only demonstrate differences between these dietary groups (frugivory and omnivory) and folivory. This needs to be explicitly stated in the relevant areas of the results section. In addition, models should be run with the levels switched so that potential differences between frugivory and omnivory can also be tested.
- Lines 287-288 and 303-304: Table 2 includes results from *best fit models only* – it would be appropriate to also mention Table 1.
- Lines 288-289: Diet is not included in the best fit model for ECV in Table 1, so I am a bit confused about the claim that diet is positively associated with all brain measures.
- Paragraph starting in Line 345: The home range results for the neocortex are not mentioned.

Discussion

- Lines 383-385: The finding that habitat variability is negatively correlated with relative brain size should be discussed in terms of previous work demonstrating a negative impact of seasonality on brain size (e.g., van Woerden et al. 2010).
- Lines 409-410: This is not true. Powell et al. (2019) found correlations between specific brain regions (neocortex) and gestation length. Other regions were correlated with other developmental periods (e.g., cerebellum and juvenile period).
- Line 421: What does “counterbalance” mean? It sounds as if animals are actively participating in the evolution of these traits. Can the authors elaborate on how specific selection mechanisms would drive this “counterbalancing”?
- Lines 426-427: This sentence makes it seem that diet category is included in the best fit models for carnivores, which is not the case. I suggest removing the sentence.
- Lines 443-446: Sociality is not included in any of the best fit models of relative brain size, so this sentence is misleading as written.
- Lines 445-457: I would remove this sentence since the cerebellum is showing opposite trends across groups.
- Paragraph starting with Line 467: As previously mentioned, previous studies have demonstrated that the use of EQ or residuals is inappropriate, so I think this paragraph and the relevant results are unnecessary and make the overall findings harder to follow.

References not included in manuscript

Van Woerden, J. T., Van Schaik, C. P., & Isler, K. (2010). Effects of seasonality on brain size evolution: evidence from strepsirrhine primates. *The American Naturalist*, 176(6), 758-767.

Smaers, J. B., Rothman, R. S., Hudson, D. R., Balanoff, A. M., Beatty, B., Dechmann, D. K., ... & Safi, K. (2021). The evolution of mammalian brain size. *Science Advances*, *7*(18), eabe2101.

Schillaci, M. A. (2006). Sexual selection and the evolution of brain size in primates. *PLoS One*, *1*(1), e62.

Schillaci, M. A. (2008). Primate mating systems and the evolution of neocortex size. *Journal of Mammalogy*, *89*(1), 58-63.

MacLean, E. L., Barrickman, N. L., Johnson, E. M., & Wall, C. E. (2009). Sociality, ecology, and relative brain size in lemurs. *Journal of Human Evolution*, *56*(5), 471-478.

Freckleton, R. P. (2009). The seven deadly sins of comparative analysis. *Journal of evolutionary biology*, *22*(7), 1367-1375.

Deaner, R. O., & Nunn, C. L. (1999). How quickly do brains catch up with bodies? A comparative method for detecting evolutionary lag. *Proceedings of the Royal Society of London. Series B: Biological Sciences*, *266*(1420), 687-694.

This manuscript has a lot of potential and I hope to see it published after revisions are made.

I hope that these comments are useful in revising your manuscript.

Sincerely,

Alex DeCasien
