# Supplementary material for: Why big brains? A comparison of models for both primate and carnivore brain size evolution
Source: PLoS One. 2021 Dec 21;16(12):e0261185. doi: 10.1371/journal.pone.0261185 (PMC8691615; doi:10.1371/journal.pone.0261185)
Supplement: Attachment — Submitted filename: Comments to the Author_PONE-D-21-12399_R1.docx [file pone.0261185.s013.docx]

**Comments to the Author**

**Review of “Why big brains? A comparison of models for both primate and carnivore brain size evolution” (PONE-D-21-12399_R1)**

The authors have thoughtfully addressed my previous comments. Accordingly, I suggest this manuscript is published following minor revisions (outlined by section below).

Introduction

- Lines 100-105: The discussion of Smaers and colleagues’ work on different evolutionary paths to relative brain size needs a bit of reorienting. As written, it sounds as if their work represents justification against using EQ and towards using another measure of relative brain size. However, they interpret their findings to mean that relative brain size is not likely to always reflect selection on cognition, and that comparisons of this measure across species with different evolutionary histories do not address this.
- Line 147: I would add that research focus on primate evolution has also resulted from anthropocentrism.

Methods

- It might be useful to note that the different groups models run separate proximate (developmental) versus ultimate (ecological, social) causes of brain size evolution.
- VIF values of 5 correspond to R^2^ = 0.8 (which seems high) – were there many models approaching this VIF value?

Discussion

- Reasons why some of the results presented here contradict those from other recent studies (e.g., neocortex size predicted by gestation length in Powell et al. 2019) should be elaborated upon here.

Tables

- The legends should be more descriptive/comprehensive (e.g., only best fit models shown, how combinations derived, etc).
- I suggest removing the asterisks denoting level of significance in Table 2.
- Please note in the legends that boldness indicates p<0.05.
  - Accordingly, the intercept and mass values should also be in bold.
- Where is ROB for the neocortex and cerebellum models? Was it not included (in contradiction to the methods) or was it accidentally omitted from the table)?

Supplement

- Table S3: It is unclear why the combined models do not represent all combinations for the social/ecological/life history best fit models (dBIC<2) – are only the combined models with dBIC<2 shown? This needs to be clarified somewhere.

I hope that these comments are useful in revising your manuscript.

Sincerely,

Alex DeCasien
