# Supplementary material for: Why big brains? A comparison of models for both primate and carnivore brain size evolution
Source: PLoS One. 2021 Dec 21;16(12):e0261185. doi: 10.1371/journal.pone.0261185 (PMC8691615; doi:10.1371/journal.pone.0261185)
Supplement: Attachment — Submitted filename: Response to Reviewers_HRC_SOH.docx [file pone.0261185.s014.docx]

**Additional Editor Comments (if provided):**

The previous reviewers have gone through the updated draft and both recognize your extensive revisions in this version. This next batch of comments are relatively minor though I do strongly agree with them that you need to spend more time detailing what is shown in your tables. I'd also advocate for including the model coefficients rather than just t-values and p-values.

**Thank you for your comment about model coefficients; we agree that this inclusion could add to our tables. Model coefficient estimates have now been added to all appropriate tables.**

**Reviewer #1:**

**Sentence altered.**

**Thank you for highlighting this, we agree this was something that needed acknowledging. A sentence has been added highlighting this and briefly justifying our use of these techniques.**

**Thank you for this comment but, as you acknowledge, it’s not necessary to refer the reader here as it’s covered elsewhere.**

**To provide greater clarity this has been altered from P to P-value in all tables.**

**Thank you for highlighting this. Table 1 and 3 have been discussed more fully in the results text.**

**Thank you for highlighting this, you are correct. The bold row represents the model with the lowest BIC score. For all carnivore results both life history and combined models should have been highlighted (dBIC <2), this has been clarified.**

**Reviewer #2:**

Introduction

- Lines 100-105: The discussion of Smaers and colleagues’ work on different evolutionary paths to relative brain size needs a bit of reorienting. As written, it sounds as if their work represents justification against using EQ and towards using another measure of relative brain size. However, they interpret their findings to mean that relative brain size is not likely to always reflect selection on cognition, and that comparisons of this measure across species with different evolutionary histories do not address this.

**Thank you for this comment, we agree the phrasing needed altering. The discussion of Smaers and colleagues’ work has been now been reorientated.**

- Line 147: I would add that research focus on primate evolution has also resulted from anthropocentrism.

**Comment added.**

Methods

- It might be useful to note that the different groups models run separate proximate (developmental) versus ultimate (ecological, social) causes of brain size evolution.

**Thank you for highlighting this, it is definitely something we should have mentioned. A sentence has been added.**

- VIF values of 5 correspond to R^2^ = 0.8 (which seems high) – were there many models approaching this VIF value?

**We only had three occurrences of scores around 5. Two in the primate endocranial volume model, with body mass producing 6.5 and weaning age producing 6, and the last in the carnivore cerebellum model, with age at first reproduction producing 5. All other scores were below 3 (see supplementary material for all VIF scores). Whilst we appreciate these scores are of slight concern, we felt it necessary and appropriate to retain all variables for further analysis because the (relatively) high scores were still only present in a few models.**

Discussion

- Reasons why some of the results presented here contradict those from other recent studies (e.g., neocortex size predicted by gestation length in Powell et al. 2019) should be elaborated upon here.

**Discussions on the potential reasoning behind the contrasting results have been added. Just to note, in order to incorporate this discussion, it was necessary to re-work the sentences and structure of the paragraph.**

Tables

- The legends should be more descriptive/comprehensive (e.g., only best fit models shown, how combinations derived, etc).

**Thank you for highlighting this, we agree the legends could benefit from having more extensive descriptions. All table legends have been updated.**

- I suggest removing the asterisks denoting level of significance in Table 2.
- Please note in the legends that boldness indicates p<0.05.
  - Accordingly, the intercept and mass values should also be in bold.

**All points here have been completed.**

- Where is ROB for the neocortex and cerebellum models? Was it not included (in contradiction to the methods) or was it accidentally omitted from the table)?

**The neocortex and cerebellum sections in the tables represent the results produced when using the ROB technique. We appreciate this was not obvious, so this has been highlighted in tables 1 and 3.**

Supplement

- Table S3: It is unclear why the combined models do not represent all combinations for the social/ecological/life history best fit models (dBIC<2) – are only the combined models with dBIC<2 shown? This needs to be clarified somewhere.

**Thank you for highlighting this, we had neglected to include all the combined models. We have now rectified this, and combined models now display all combinations of the social/ecological/life history best fit models (seen in the BIC score excel files).**
